# Single‐Stranded Interruptions in the T7 Promoter Non‐Template Strand Exhibit Unexpected Transcription Behavior

**DOI:** 10.1002/anie.2229119

**Published:** 2026-09-15

**Authors:** Michael W. Haydell, Viktoria Steuper, Michael Famulok

**Affiliations:** ^1^ LIMES Chemical Biology Unit Universität Bonn Bonn Germany

**Keywords:** DNA structures, RNA recognition, transcription regulation, T7 RNA polymerase

## Abstract

T7 RNA polymerase (T7RNAP) is widely used for in vitro transcription due to its high specificity and efficiency. Its ability to initiate transcription from a promoter containing single‐stranded interruptions, however, remains largely unexplored even though such an ability might provide a potential mechanism for transcriptional regulation in vitro. Here, we systematically introduce single‐stranded gaps of varying sizes and positions within the non‐template strand of the T7 promoter to investigate their effects on transcription, hypothesizing that small gaps would essentially be tolerated while large gaps would be inhibitory. Surprisingly, single‐nucleotide gaps in the promoter's specificity region (−11 to −8) prevented transcription and larger gaps allowed transcription. Fluorescence and gel‐based assays confirmed these effects. Structurally, small gaps may disrupt essential hydrogen bonding while larger gaps may increase promoter interaction with T7RNAP, thereby improving binding and initiation while hindering promoter release. These insights reveal new aspects of T7RNAP function and suggest novel strategies for DNA‐based transcriptional control in bio‐hybrid systems, which we demonstrate by introducing an RNA‐dependent transcription‐based molecular switch.

## Graphical abstract

DNA structures with short single‐strand gaps in the non‐template strand of the T7 promoter prevent transcription by T7 RNA polymerase. Contrary to intuition, however, transcriptional activity can be observed again when the size of the gap increases toward the transcription start site, thereby reducing the proportion of double‐stranded DNA in the promoter region. This unexpected effect can be used to engineer an RNA‐dependent molecular switch.

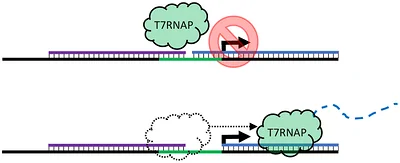

T7 RNA polymerase (T7RNAP) is widely used for in vitro transcription (IVT) in synthetic biology [1], molecular biology and biotechnology [2, 3], and in DNA nanotechnology [4, 5, 6, 7]. Its behavior [8], mechanics [9], promoter interactions [10, 11, 12, 13], and limitations [14, 15] have been extensively studied. Atypical structures such as branches [15, 16], nicks [15, 16, 17], and single‐stranded regions [8, 14, 15, 16, 18] affect T7RNAP's ability to transcribe a DNA substrate, but most work has focused on modifications to the template (T) strand and/or outside the promoter. These studies showed that interruptions in the T strand initiation region reduce the number of abortive starts [16] but also impair RNA displacement and that T7RNAP can bypass gaps [18] or even switch template strands [15]. Few studies have altered the non‐template (NT) strand promoter, and those changes were comparatively minor, such as a nick [16], a gap [19], or simple 5’ [8] or 3’ end [8, 14] shortening. These studies found that T7RNAP transcription decreases [8, 14] the more single‐stranded the promoter becomes. Base pair substitutions inside the promoter are tolerated at either position −12 or −6, but detrimental between −11 and −7 [13]. Studies adjusting promoter length upstream [20] and downstream [21] of the +1 transcription start site can tune initiation, but relied on hairpins or were aimed at enzyme optimization and used fully duplexed promoters.

Understanding how single‐stranded interruptions (henceforce referred to as “gaps”) in the promoter NT strand affect IVT can lead to useful insights for transcription‐based systems such as regulation [5, 22], improving efficiency in vitro [21, 23], or as control systems for T7RNAP‐driven bio‐hybrid DNA machines [4, 6, 24]. As mentioned, a systematic understanding of how gaps in the promoter NT strand affect transcription is missing. Based on previous studies, we hypothesized that T7RNAP can tolerate smaller gaps [13, 15, 18] in the promoter region but larger gaps [8, 14, 20] would be detrimental to transcription. To test this hypothesis, we systematically introduced single‐nucleotide (nt) gaps at each position within the promoter NT strand then increased the gap size by one nt until position +1. Transcription substrates (Figures 1a and S1–S13) were constructed from synthetic DNA (Table S1) and included a T strand with three regions: a handle region, the promoter region, and a molecular beacon (MB) target region. The T strand hybridizes to two NT strands: a 5’ coding strand pairing from −40 to −18+#, where # is how many base pairs (bp) the promoter is duplexed, and a 3’ coding strand hybridizing to the rest of the promoter and the MB region. Such structures are called # bp promoters. Transcription of the MB target region produces an RNA strand complementary to a 2’ OMe RNA hairpin MB (Figures 1b and S14) bearing a 5’ fluorescein (FAM) and a 3’ DABCYL (4‐((4‐(dimethylamino)phenyl)azo)benzoic acid) quencher, present in solution. Hybridization with the transcribed RNA opens the MB, increasing FAM fluorescence and enabling real‐time transcription monitoring.

**FIGURE 1:**
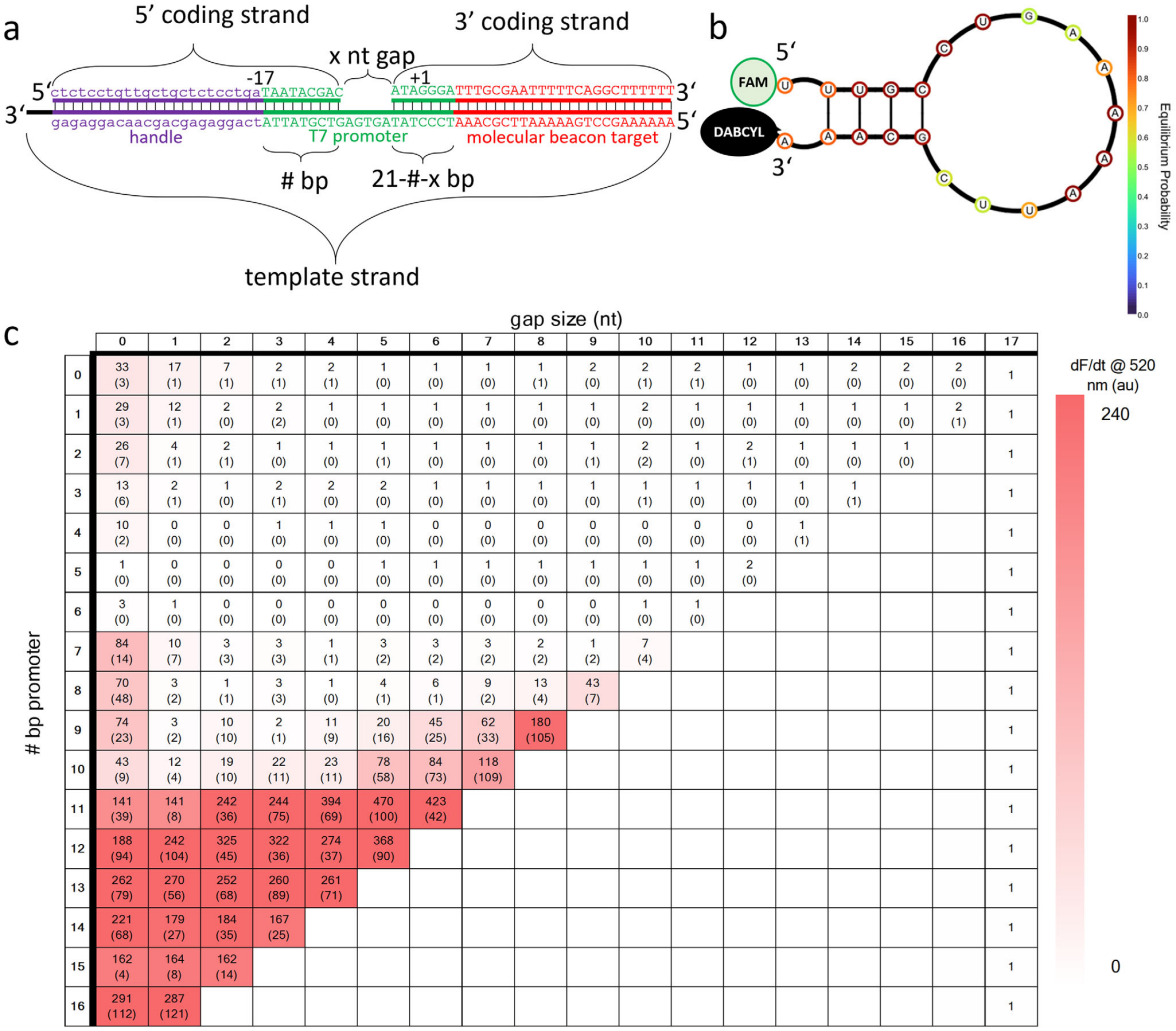
Single‐stranded gaps in the promoter region NT strand of DNA substrates affect T7RNAP transcription rate. (a) Schematic explaining the naming convention for a # bp promoter with an x nt gap transcription substrate. The example shown is a 9 bp promoter with a 5 nt gap. (b) Nupack [25, 26, 27] schematic showing a 2’OMe RNA, 5’ fluorescein (FAM), 3’ (4‐((4‐(dimethylamino)phenyl)azo)benzoic acid) (DABCYL), molecular beacon (MB) used as a reporter for tracking the (c) T7RNAP transcription rates for structures with indicated lengths of double‐stranded DNA in the promoter region (# bp promoter, see Figures S1–S13) interrupted by gaps of indicated sizes, arranged in a heat map. Values displayed are the mean slope of FAM fluorescence increase during the linear phase [dFI/dt (au @ 520 nm)], normalized to the 17 nt gap negative control (17), *n* = 3. Values in parentheses are s.d, discussed in Supporting Text ST1.1. Raw transcription data available in Figures S15–S31. Base pair colorings in (b) are equilibrium probabilities calculated by Nupack for 2’Ome‐RNA at 30°C in 120 mM NaCl.

Contrary to our hypothesis, Figure 1c shows that T7RNAP cannot tolerate even a 1 nt gap in the promoter region when fewer than 10 bp from the 5’ end are hybridized. Specifically, the transcription rate during the linear phase for 8 or 9 bp promoter substrates with a 1 nt gap is essentially indistinguishable from the fully single‐stranded promoter negative control (17 nt gap). Additionally, a 1 nt gap markedly reduces the transcription rate for a 10 bp promoter. Unexpectedly, 9 bp promoters exhibit a progressive increase in initial transcription rate for gap sizes larger than 4 nt, reaching levels comparable to a nicked (0 nt gap) substrate for 7 nt gaps. 9 bp promoters with 8 nt gaps initially transcribe faster than the nicked substrate.

These observations contradict the hypothesis that longer single‐stranded regions within the NT‐promoter would be detrimental to the transcription rate [8, 14, 20]. 8 bp promoters with large gaps do not recover transcription as effectively as the 9 bp promoter, likely due to insufficient base pairing to stabilize the promoter structure. Substrates with 7 bp promoters and below cannot support transcription for gap sizes larger than 0 nt, with the exception of 0, 1, and 2 bp promoters, discussed in Supporting Text ST1 (Figures S15–S17), while those with 11 bp promoters and larger can tolerate any gap size. These findings reveal a nuanced dependence of transcription on gap size and position within the NT strand promoter.

We also compared how a nick or 1 nt gap affects transcription as it moves throughout the promoter against a fully double‐stranded positive control. The results, discussed in Supporting Text ST2 (Figures S32–S35), show that the transcription rate of a substrate with a nick or gap at or downstream of position ‐5 greatly exceeds that of the fully double‐stranded positive control, and nick or gaps at other positions behave in agreement with the results of Figure 1c. Furthermore, we recognize that this discovery is counterintuitive to expectations based on previous studies [8, 13, 14, 15, 18, 20]. We therefore performed independent verification experiments employing more direct transcription tracking methods, namely pepper fluorogenic aptamer [28] transcription and polyacrylamide gel electrophoresis (PAGE).

We selected the 9 bp promoter for verification experiments because it showed the most pronounced difference in transcription rates between small and large gaps. Three substrates were tested: one with 0, 1, and 8 nt gap. The MB region was replaced with a pepper fluorogenic aptamer sequence (Figure S36) which fluoresces upon binding to the dye [4‐((2‐hydroxyethyl)(methyl)amino)‐benzylidene]‐cyanophenylacetonitrile] (HBC). The transcription rate was monitored at 617 nm upon excitation at 578 nm. The substrate with a 0 nt gap (Figure 2a, 0) transcribed similarly to the fully double‐stranded positive control (P) while that with the 1 nt gap (1) transcribed far slower than either the positive control, the nicked sample (0), or the substrate with an 8 nt gap (8) during the linear phase (Figure 2a). The raw data for these tests (Figure S37) reveal that the 8 nt gap substrate plateaus early, likely due to reduced promoter release thereby preventing transition to elongation [19], discussed in Supporting Text ST3 (Figures S37–S39). To further validate these results, the substrates also were transcribed and analyzed by HBC‐stained PAGE. The annealed substrates were split in half and one aliquot transcribed (Figure 2b T) and the other not. As shown in Figure 2b (additional gels: Figure S38), RNA bands of the expected length (R, synthetic pepper aptamer marker) are visible in the transcribed lanes for the positive control (P), 0 nt gap lanes (0), and less intensely in the 8 nt gap substrate lane (8). Furthermore, only very faint bands are present in the negative control (N) or 1 nt gap (1) lanes. The low‐mobility band is likely the pepper aptamer interacting with the T7RNAP [29], since that band is present in the R+T7 lane but not in the R‐T7 lane. We also ruled out incomplete hybridization and the results are discussed in Supporting Text ST4 (Figures S40–S49). Lastly, we explored T7RNAP binding strength to various DNA substrates with pull‐down assays using the polymerase's His_6_‐tag to immobilize it on Ni‐NTA beads and the results are discussed in Supplementary Text ST5 and Figures S50–S53. Together, the pepper aptamer, gel‐based transcription, hybridization, and pull‐down assays demonstrate that small gaps in a 9 bp promoter severely impair transcription initiation, whereas larger gaps restore initiation‐associated transcriptional activity, although productive synthesis of full‐length RNA remains reduced, consistent with reduced promoter release.

**FIGURE 2:**
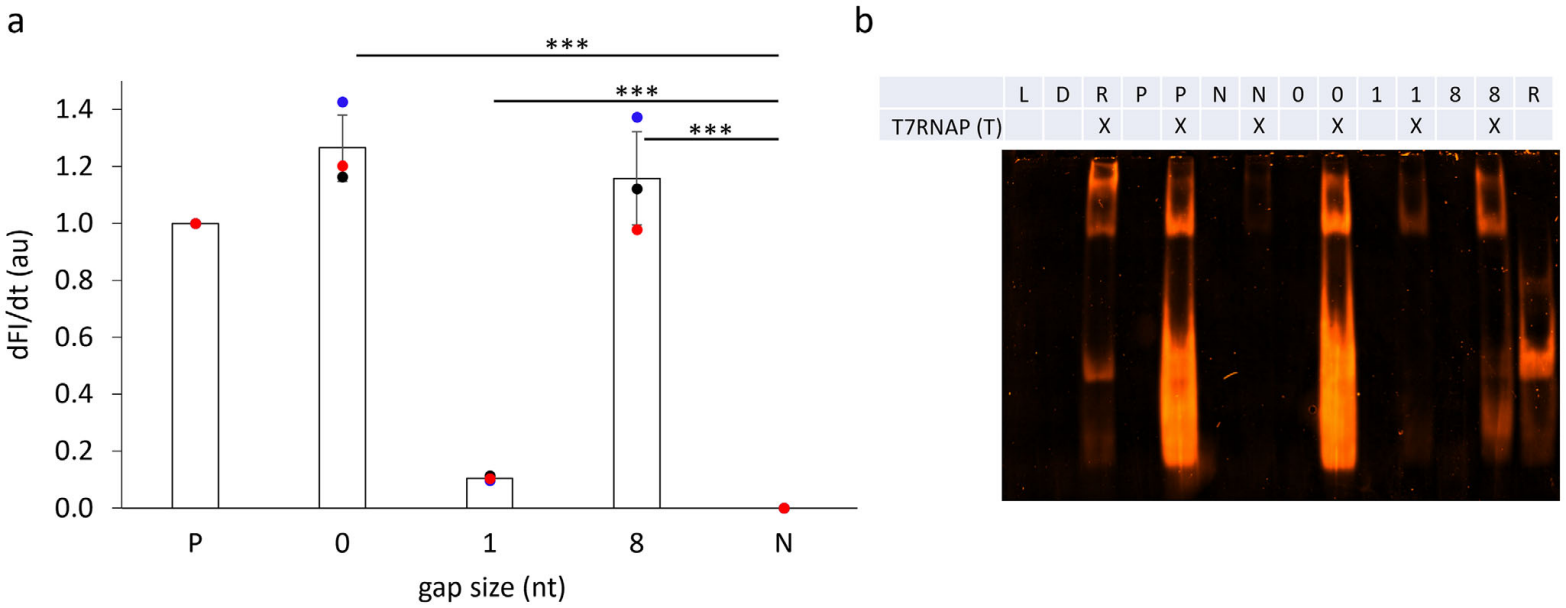
Direct transcription tracking of 9 bp promoter structure with a 0 (0), 1 (1), or 8 (8) nt gap. (a) T7 transcription rate during linear phase range normalized to the fully double‐stranded positive control (P) and 17 nt gap negative control (N) using pepper aptamer fluorescence (ex 578 nm, em 617 nm). *n* = 3, bar graphs are mean, error bars are s.d. Significance, one‐tailed homoscedastic *t*‐test; ***: p<.001. Raw data in Figure S37. (b) Native PAGE gel showing T7RNAP transcription (T) of 9 bp promoter encoding pepper aptamer. L is ultra low range DNA Ladder, D is DNA marker of expected transcription product, R is RNA marker of expected transcription product. 8% PAGE, 100 V, 30 min, HBC‐stained. Additional gels in Figure S38.

We next investigated whether this phenomenon could be harnessed to regulate T7 transcription in response to an RNA input. As a proof‐of‐principle, we designed a molecular switch based on a gap‐containing promoter. We selected a promoter structure with a gap that does not transcribe (0 bp promoter/9 nt gap) and engineered a mechanism that “fills” the gap upon addition of an input signal, effectively creating a 9 bp promoter/0 nt gap, The design (Figure 3a) consisted of a hairpin structure with the T7 promoter bases −17 to −9 in the loop (HP, green loop) and the stem (HP, blue) with 18 bp of hybridization, which was bound to the template strand via a handle region (purple). Upon addition of the input signal (HPin, input RNA), the input RNA strand (dashed) hybridizes to a 7 nt toehold directly 5’ of the hairpin stem (HPin) and opens the hairpin, thereby exposing the first 9 bases that are complementary to the single‐stranded region of the template strand's promoter sequence. The opened loop can now hybridize to the gapped promoter (HPin, green) and allow transcription of the MB (red). Without input RNA (HP) the transcription rate is similar to the negative control (neg), while in presence of input RNA (HPin) the signal rises similarly to the positive controls (ds pos, pos). The input RNA was also added to the negative control sample to test the input's ability to open the MB on its own, which it obviously does not do. Although this switch is presented as a proof‐of‐principle rather than an optimized transcription device, these results demonstrate that the newly identified gap‐dependent promoter behavior can be harnessed for programmable, input‐dependent regulation of T7 transcription.

**FIGURE 3:**
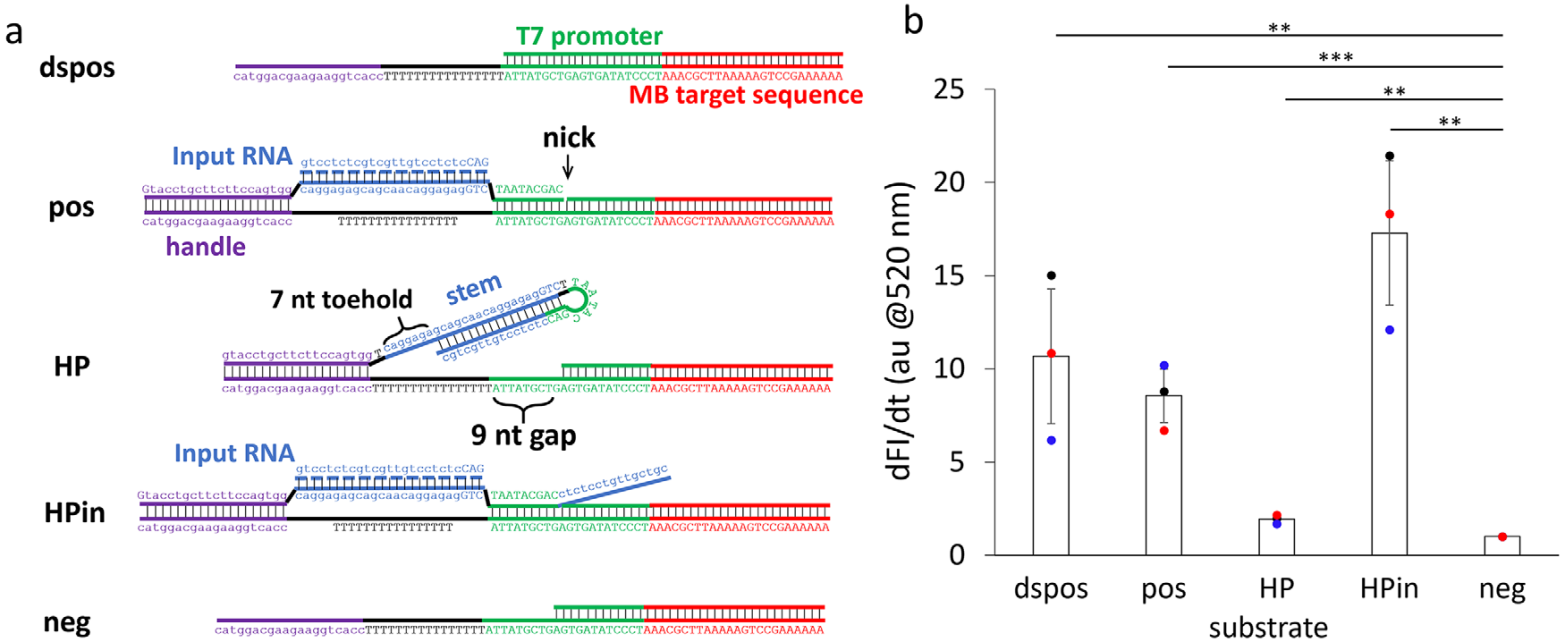
Controlling transcription with a hairpin‐based switch. (a) Schematics of structures used for demonstrating control of transcription based on excluding (HP) or including (HPin) an RNA input strand (dashed blue line). (b) T7 transcription rate during linear phase normalized to the negative control (neg) using FAM fluorescence. The positive control (dspos) has a fully double‐stranded promoter and an additional positive control (pos) contains the same upstream sequence context as the switch substrate, but without the 3’ overhang so that the hairpin cannot form. *n* = 3, bar graphs are mean, error bars are s.d. Significance, one‐tailed homoscedastic *t*‐test; **: *p* < 0.01, ***: *p* < 0.001. Raw data in Figure S54.

We examined T7RNAP's crystal structure [10] (Figure 4) to understand why small gaps or nicks in the NT strand promoter are not tolerated at certain positions. The specificity loop plays a crucial role in promoter recognition [10], with residues N748, R756, and Q758 forming hydrogen bonds in the promoter specificity region (Figure 4a) at positions −11 to −7, primarily on the T strand. There is, however, a key hydrogen bond between N748 and G‐11, partly mediated by a water molecule [10]. Most contacts are between the polymerase and T strand, but proper duplex integrity appears necessary for accurate positioning. Disruptions in the duplex therefore undermine promoter recognition. Structural analysis of the phosphate backbone just 5’ of position −12 (Figure 4b), shows dense hydrogen bonding between the NT strand backbone and amino acid residues K98, T101, A102, H211, R215, N748, and M750. Our results (Figure S35) show that these interactions are vital to polymerase activity and depend on phosphate backbone continuity.

**FIGURE 4:**
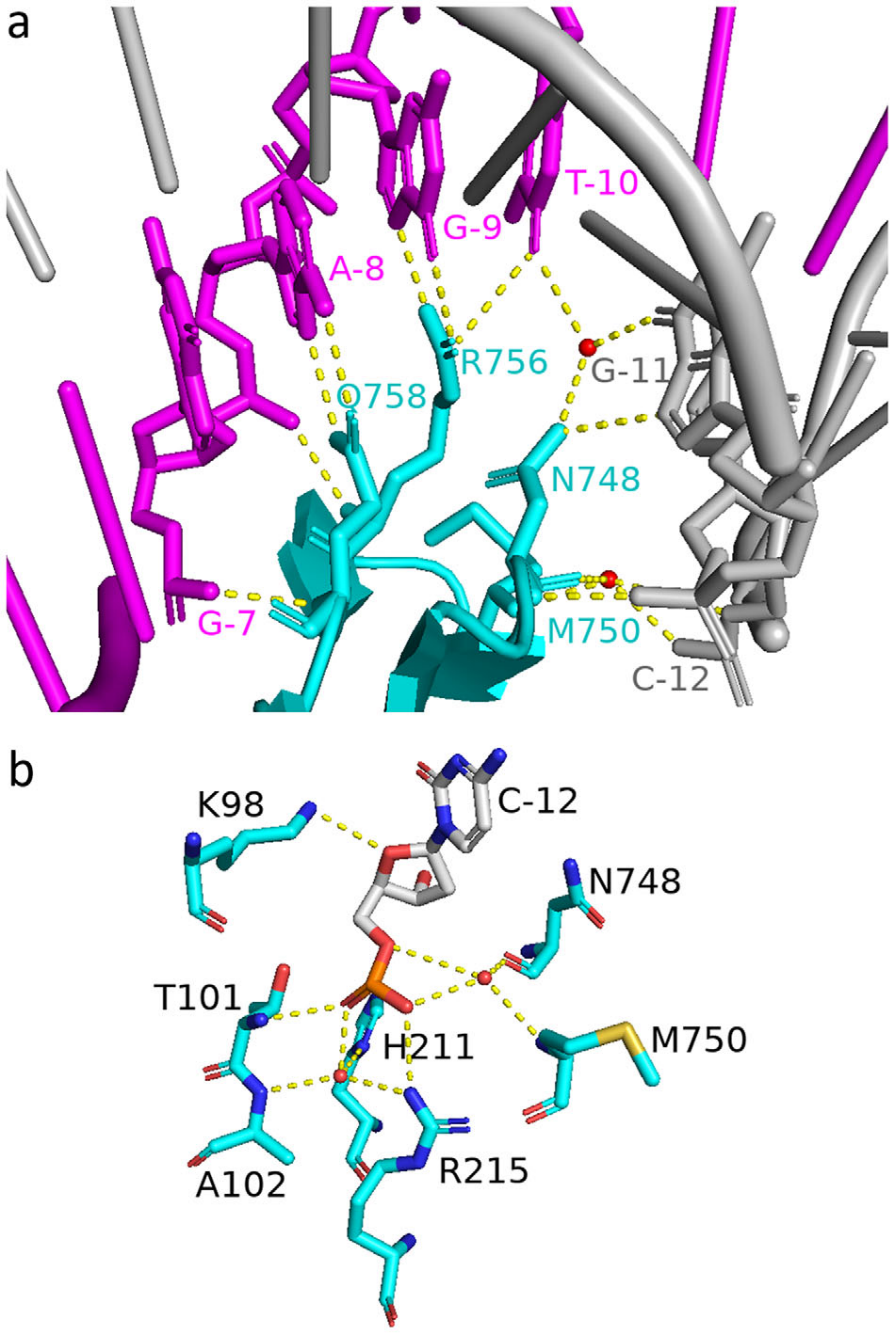
Pymol representation of T7RNAP (cyan carbons) crystal structure (1CEZ) detailing hydrogen bonding (yellow dashed lines) between the template (magenta carbons) and NT (grey carbons) strands in the (a) promoter specificity loop and (b) at position −12 in the NT strand. Red dots are water molecules.

It is known that partially single‐stranded promoters form strong interactions with the T7RNAP and prevent the polymerase from releasing the promoter and entering the elongation phase [19]. As the 9 bp promoter substrate is single‐stranded in the initiation region, it is possible the polymerase never releases the promoter and remains in the initiation phase. Since T7RNAP bound to the promoter can still transcribe RNA even up to 26 nt long, the MB target strand continuously gets transcribed without the polymerase “running off” the template. This means that T7 diffusion is no longer a limiting factor once transcription begins. Therefore, gapped substrates may be particularly useful for signal transduction applications using short RNA strands.

Other potential mechanisms may include steric hinderance between a rigid promoter duplex and amino acid residues 595–608 (which form a loop at the T7RNAP active site exit) [30] could obstruct promoter docking. A nick or gap in the promoter initiation region (−5 to −1) increases DNA flexibility, improving T7RNAP's access to the promoter specificity region and lowering the energetic cost of forming the transcription bubble. Furthermore, the flexibility of large gaps in 8, 9, or 10 bp promoter substates may help stabilize the “broken” duplex when docked in the active site, allowing key hydrogen bonds to reform and restoring promoter recognition. Lastly, we note that promoter‐independent transcription [31, 32] can occur at overhangs, unpaired regions, or termini, but was controlled for through the negative control. Therefore, the observed transcription behavior is specific to the promoter recognition of T7RNAP.

We report a previously unrecognized and surprising finding that single‐nucleotide gaps at specific positions in the T7 promoter's NT‐strand drastically reduce transcription efficiency, while larger gaps in the second half of the promoter allow transcription to proceed. We used fluorescence and gel electrophoresis to verify these claims, and we confirmed that they stem from gap size rather than incomplete strand hybridization.

While our results do not contradict any previous studies, they are surprising if one uses those previous studies to form a hypothesis of how a gap in the NT strand promoter region affects transcription. Namely, it is known that transcription fails when the promoter becomes single‐stranded upstream of position ‐9 [8, 14]. We replicate this result (Figure S46), but went a step further by restoring duplex DNA at the +1‐start site. Hypothetically, if 9 bp of duplexed promoter is insufficient to initiate transcription, then this new structure should also fail. Instead, transcription clearly occurs (Figures 1c and S24). Furthermore, prior work suggests that increasing duplex length [18], that is, shrinking the gap, should increase transcription rates. Again, our results contradict this hypothesis because extending duplexed DNA from +1 toward the recognition region reduces transcription until, for a 1 nt gap, the rate is not significantly different from the negative control (Figure S24). In fact, our findings build upon previous studies [18, 19], since the gaps in such studies were primarily outside of the promoter region, while our gaps were constrained to and moved within the promoter region.

Our findings may inform studies of T7RNAP mechanism and open new avenues for transcription regulation. Future studies applying similar tests to other phage polymerases, such as T3 and SP6, could reveal whether this promoter sensitivity is a generalizable feature across the family. Additionally, this work has potential implications for T7RNAP‐driven DNA‐based nanomachines, where our results could offer a novel method of control, as demonstrated by our engineered RNA‐dependent transcription‐based switch.

## Author Contributions

**Michael W. Haydell**: conceptualization, investigation, writing – original draft, methodology, validation, visualization, data curation, writing – review and editing, formal analysis, and supervision. **Viktoria Steuper**: investigation, methodology, validation, visualization, formal analysis, and data curation. **Michael Famulok**: conceptualization, investigation, funding acquisition, writing – original draft, methodology, validation, visualization, writing – review and editing, formal analysis, project administration, data curation, supervision, and resources.

## Conflicts of Interest

The authors declare no conflicts of interest.

## Supporting information



The Supporting Information provides experimental details on the methods used, a list of sequences, structure schematics, as well as additional data and discussions mentioned in the main text. The supporting data file provides the raw data.**Supporting File 1**: anie74782‐sup‐0001‐SuppMat.pdf.

**Supporting File 2**: anie74782‐sup‐0002‐SuppMat.xlsx.

## Data Availability

The data that supports the findings of this study are available in the Supporting Information of this article.
