# Supplementary material for: Single‐Stranded Interruptions in the T7 Promoter Non‐Template Strand Exhibit Unexpected Transcription Behavior
Source: Angew Chem Int Ed Engl. 2026 Sep 15;65(44):e2229119. doi: 10.1002/anie.2229119 (PMC13703809; doi:10.1002/anie.2229119)
Supplement: Supplementary file 1 [file ANIE-65-e2229119-s001.pdf]

Single-stranded interruptions in the T7 promoter non-template strand exhibit unexpected transcription behavior

Michael W. Haydell, Viktoria Steuper, Michael Famulok*

Table of Contents: items below are hyperlinks to respective page

Methods ... 2
Abbreviations ... 3
Signpost ... 4
Table S1: DNA sequences ... 5
Figures S1-S13: Structure Schematics ... 7
Figure S14: MB schematic ... 20
Supplementary Text ST1: concerning 0,1, and 2 bp promoter structures ... 21
Supplementary Text ST1.1: concerning the large variations in transcription rate ... 21
Figures S15-S31: Raw data for molecular beacon transcription tracking ... 22
Supplementary Text ST2: concerning nick and 1nt gap moved throughout promoter sequence ... 39
Figure S32: Positive control schematic ... 39
Figure S33: Transcription tracking of nick moving through promoter ... 40
Figure S34: Transcription tracking of 1 nt gap moving through promoter ... 41
Figure S35: Phosphorylation and ligation of nick ... 42
Supplementary Text ST3: on early plateauing mentioned in main text ... 43
Figure S36: Pepper aptamer transcription substrate schematics ... 44
Figure S37: Pepper aptamer transcription tracking ... 45
Figure S38: Original gels for transcription verification ... 46
Figure S39: Transcription quantification and reagent variation ... 47
Supplementary Text ST4: on hybridization verification experiments: ... 48
Figure S40: Melting curves of template to individual coding strands ... 49
Figures S41-S45: Hybridization verification gels ... 50
Figure S46: Transcription rate of template to individual coding strands ... 55
Figure S47: Transcription tracking raw data of template to individual coding strands ... 56
Figures S48-S49: Schematics for transcription tracking of template to individual coding strands ... 57
Supplementary Text ST5: on pull-down binding assays: ... 59
Figure S50: Binding studies ... 59
Figures S51-S52: Pull down assay for T7RNAP binding to substrates ... 60
Figure S53: Transcription tracking of pull down assay substrates ... 62
Figure S54: Transcription tracking for transcription-based switch ... 63
REFERENCES ... 64

Methods

Substrate Preparation: The DNA used in this study was purchased from biomers.net as lyophilized after cartridge purification or HPLC purification (bold name in Table S1). The sequences in Table S1 are combined to form the structures (Figures S1-S13) used in this study. Substrates are prepared at 300 nM in 1x assembly buffer (5 mM Tris, 10 mM $MgCl_2$, 50 mM NaCl, pH 7.9) with the template strand at 1.5 molar excess (450 nM) to sequester out any 3' coding strands which themselves are complementary to the molecular beacon (MB) probe, heated to 95 °C for 60 seconds and then incubated at 4 °C for 30 minutes. Substrates were freshly annealed before each experimental replicate. Substrates for pepper transcription were diluted twofold or tenfold in 1x assembly buffer. Note: both template and Temp2 in Table S1 were used as template strands as they have identical sequences except for 3 nucleotides (GTA, highlighted gold) in the black (unused) region. Those extra 3 bases do not play a role in the work presented in this paper.

The MB, sequence in Table S1 and Figure S14, was ordered as lyophilized after HPLC purification from Ella Biotech and is 2' OMe modified at every base with a 5' 6FAM modification and a 3' DABCYL quencher modification. Before use, the MB was diluted to 50 µM in 1x assembly buffer, heated to 50 °C for 5 minutes, then incubated at 4 °C for 30 minutes to ensure proper annealing, and stored at -20 °C. NTPs were purchased from Carl Roth GmbH + Co. KG.

The recombinant T7RNAP was expressed in *Escherichia Coli* and purified with Ni-NTA beads by N. Kuhn and its activity compared against New England Biolabs T7RNAP M0251, activity ~50 U/µL.

For the transcription-based switch experiments, the 1 µL of input RNA (30 µM) was added immediately before substrate addition and was at 10x excess over the substrate.

Transcription Assays: A master mix (transMM) was prepared containing the transcription components (~50 U T7RNAP, 0.33 µM molecular beacon [MB, replaced with 0.66 µM HBC620 in the case of pepper fluorescent tracking], 0.5 mM ATP, 0.5 mM CTP, 0.5 mM GTP, 0.5 mM UTP, 1 mM TCEP, 40 mM Tris pH 7.9, 50 mM NaCl, and 10 mM $MgCl_2$) from which 20 µL of transMM was combined with 10 µL of substrate (100 nM final substrate concentration for MB transcription tracking and 100, 50, or 10 nM for pepper transcription tracking under various conditions) and measured with a BioRad CFX96 Real time System PCR machine with a C1000 Touch Thermal Cycler set to measure SYBR Green in the case of FAM molecular beacon fluorescent tracking or in a TECAN Spark Cyto cell imager plate reader set to 578 nm excitation and 617 nm emission for pepper fluorescent tracking. Measurements were made every 2 minutes at 30 °C.

Gel Electrophoresis Transcription Assays: Samples for transcription tracking via gel electrophoresis were prepared identically as to the fluorescent methods, except no MB or HBC was added to the transMM. Moreover, a second transMM was made without T7RNAP in which no transcription can occur. Each substrate sample was divided in half by volume, to which one half received the transMM+T7 and the other half received the transMM-T7. After combining substrate and transMM, samples were transcribed for 2 hours at 30 °C. 1 pmol was added to an 8% PAGE and ran for 30 minutes at 100 V (13.7 V/cm) stained with HBC (30mL 40 mM HEPES, 100 mM KCl, 5 mM MgCl2, 10 µM HBC, pH 7.8) for 15 min and imaged on an Invitrogen iBright FL1500 imaging system exciting with the 515-545 nm band and measuring emission in the 568-617 nm band. The gel was then stained with SYBR Gold (Thermo Fischer) and imaged on a VWR Genoplex Gel Documentation System with 2 sec of exposure. The ladder is a GeneRuler Ultra Low Range DNA ladder from Thermo Scientific, cat no. SM1213.

Samples in the gels shown in Figures S38 a-c were processed slightly differently after transcription. Each sample was again split in half and 1.7 µL of 10x DNAse1 buffer (New England Biolabs B0303S) added to each. One half received 1µL of DNAse1 (New England Biolabs M0303S) while the other half 1µL of 1x DNAse1 buffer. The samples were digested for 4 hours at 37 °C after which 500 fmol was mixed with 5µL of 2x RNA loading dye, heated to 50 °C for 5 min and loaded on a 15% Urea PAGE (with a 4% PAGE stacking layer) and ran for 90 minutes at 100 V (13.7 V/cm) stained with SYBR Gold (Thermo Fischer) and imaged on a VWR Genoplex Gel Documentation System with 1 sec of exposure.

Substrate Melting Temperature Analysis: The verify that undesired species are not affecting the results, the substrates were prepared as described above with the template hybridized to only the 5' coding strand or only the 3' coding strand. 5 µL of each sample was combined with 5 µL of 2x Green Dye (Genaxxon Biosciences) and subjected to a melting curve program (25 °C to 90 °C at 1 °C/5 seconds) on the BioRad CFX96 Real time System PCR machine with a C1000 Touch Thermal Cycler measuring SYBR green fluorescence to track the decrease in intercalated Green Dye fluorescence as the amount of dsDNA decreases during melting.

Ligation Assay: The nick just 5' of positions -12 and -11 was ligated and the transcription activity measured both with and without ligation in 5 and 6 bp promoter structures using a 5' phosphorylated version of pro16MB or pro15MB, respectively. Ligation was performed by combining 10 µL of substrate with 10 µL of 2x DNA ligation buffer from Roche Rapid DNA Dephos and Ligation kit 04898125001 and 1 µL of T4 DNA ligase of the same kit. Non-ligated samples were diluted with 10 µL of 1x assembly buffer. Ligation was performed for 1 hour at 20 °C. Samples were transcribed as described above.

Agarose Gel Hybridization Verification: Samples were prepared with template strand at 1.5x in 1x assembly buffer (described in Materials and Methods), heated to 95 °C and annealed at 4 °C for 30 minutes. 500 fmol of each sample was loaded on the gel, except for the coding strand alone lanes to which 1500 fmol was added (to increase staining). 3% agarose, 1x TAE, 80 V, 60 or 40 minutess, pre-stained with SYBR Gold. Imaged on a FujiFilm FLA-3000 phosphoimager set to fluorescent reading at 473 nm excitation, Y520 filter, 1000 sensitivity, 16 bits/pixel, 50 µm pixel size. The ladder is a GeneRuler Ultra Low Range DNA ladder from Thermo Scientific, cat no. SM1213.

Pull-Down Binding Assays: Per sample, incubated 1 µL of Dynabeads with 150 pmol of T7RNAP in 10 µL of washing buffer (1 mM TCEP, 10 mM $MgCl_2$, 50 mM NaCl, 40 mM Tris) at 25 °C for 10 minutes. Evenly aliquoted the solution into fresh tubes (one per sample) and incubated on magnetic rack for 2 minutes, discarded supernatant. Washed each sample by incubating with 20 µL of washing buffer for 2 minutes at 25 °C, placed on magnetic rack for 2 minutes, subsequently discarded supernatant. Incubated 150 pmol of DNA in 20 µL of washing buffer with respective samples for 10 minutes at 25 °C (to T7RNAP only sample, added only 20 µL of washing buffer). Placed on magnetic rack for 2 minutes, subsequently discarded supernatant. Washed each sample, as described before, once. Eluted by incubating with 20 µL of elution buffer (300 mM Imidazole, 50 mM Na_2PO_4, 300 mM NaCl, 500 µL Tween) at 25 °C for 5 minutes. Placed on magnetic rack for 2 mins, retained supernatant. Ran 10 µL of each sample (300 fmol of DNA only as reference next to respective sample) on 8% native PAGE at 100 V for 35 minutes, stain with SYBR Gold. imaged on a FujiFilm FLA-3000 phosphoimager set to fluorescent reading at 473 nm excitation, Y520 filter, 1000 sensitivity, 16 bits/pixel, 50 µm pixel size. The ladder is a GeneRuler Ultra Low Range DNA ladder from Thermo Scientific, cat no. SM1213. Quantification of bands was performed using AIDA software across 3 different gels.

Data Processing: For graphs showing transcription rate during the linear phase, the linear phase is determined by the early stages of transcription where the increase appears as a straight line. The time point where the linear phase ends was determined manually for each experimental run based on the fastest increasing curve, and is provided in the data file and additionally as red dashed lines in the raw data curves in the supplemental information file. The rate was determined by taking the absolute value of the fluorescence intensity value at this time point minus the initial value of the negative control (where all values "should" start upon transcription initiation) and dividing by the time point. The 17 nt gp negative control slope always used time point 120 minutes. These rates were then normalized to the negative control by dividing by the negative control rate. The 120 minute timepoint was also selected for the 5 and 6 bp promoter structures in order to be the most conservative and give the data the worst possible chance at being considered significant, since none of the substrates for those structures exhibited a clear linear phase of transcription. For the ligation assay, the data was range normalized (sample – negative control)/(positive control – negative control) to better reflect rate trend, as opposed to magnitudes, between the experimental repeats. For the switch, the timepoint for all samples used the point chosen for the fastest transcribing sample in order to give the negative control the highest possible, and therefore most conservative, "transcription" rate.

Abbreviations

DNA, deoxyribonucleic acid; RNA, ribonucleic acid; T7RNAP, T7 RNA polymerase; IVT, in vitro transcription; nt, nucleotide; MB, molecular beacon; bp, base pair; FAM, fluorescein amidite; DABCYL, (4-((4-(Dimethylamino)phenyl)azo)benzoic acid); s.d., standard deviation; PAGE, polyacrylamide gel elec-trophoresis; HBC, (4-((2-hydroxyethyl)(methyl)amino)-benzylidene)-cyanophenyl-acetonitrile.

Here is a signpost for more easily navigating the SI to find figures for schematics, raw data, or control experiments

<u>Schematics</u>

Molecular beacon (MB) transcription, Figure 1C	Figures S1-S14
Positive Control, Figures S33, S34, S35, S46, S47	Figure S32
Pepper Transcription, Figure 2	Figure S36
Template hybridized to 5' coding strand, Figures S40, S46, S47	Figure S48
Template hybridized to 3' coding strand, Figures S40, S46, S47	Figure S49
Binding studies, Figures S50-53	Figure S53
Transcription-based switch, Figure 3	Figure S54

<u>Raw Data</u>

MB transcription tracking, Figure 1C	Figures S15-S31
Pepper transcription tracking, Figure 2A	Figure S37
Gel based pepper transcription tracking, Figure 2b	Figure S38
Input mediated regulation, Figure 3	Figure S54

<u>Control Experiments</u>

How a nick's location affects transcription	Figure S33
How a 1 nt gap's location affects transcription	Figure S34
Ligating nicks	Figure S35
Pepper transcription, quantification/variable conditions	Figure S39
Melting curves for strand annealing	Figure S40
Gels showing annealing success	Figures S41-45
Transcription rate of individual coding strands	Figures S46, S47
Binding studies	Figures S50-S53

Table S1: List of DNA sequences used in this study. Colored regions indicate complementarity: Red = MB target (or pepper) sequence, green = T7 promoter, purple = handle, black= unused (Template/Temp2), or poly T spacer sequence (Switch Temp), blue=input, the toehold sequence on the switch hairpin is underlined, o=2'OMe. 5' coding strands are named as #pro where # is how many base pairs into the promoter sequence (from the 5' end) are duplexed. 3' coding strands are named as pro#MB where # is how many base pairs into the promoter sequence (from the 3' end) are duplexed. In all subsequent figures, the black template strand is either "Template" or "Temp2" unless otherwise mentioned. Bold name indicates that oligonucleotide is HPLC purified, all others are cartridge purified.

Name	Sequence (5' -> 3')
**Template**	AAAAAAGCCTGAAAAATTCGCAAATCCCTATAGTGAGTCGTATTATCAGGAGAGCAGCAACAGGAGAGGTCTTTTTCCACTGGAAGAAGCAGGTAC
**Temp2**	AAAAAAGCCTGAAAAATTCGCAAATCCCTATAGTGAGTCGTATTATCAGGAGAGCAGCAACAGGAGAGGTCGTATTTTTCCACTGGAAGAAGCAGGTAC
ProMB	TGCTGCTCTCCTGATAATACGACTCACTATAGGGATTTGCGAATTTTTCAGGCTTTTTT
0pro	CTCTCCTGTTGCTGCTCTCCTGA
1pro	CTCTCCTGTTGCTGCTCTCCTGAT
2pro	CTCTCCTGTTGCTGCTCTCCTGATA
3pro	CTCTCCTGTTGCTGCTCTCCTGATAA
4pro	CTCTCCTGTTGCTGCTCTCCTGATAAT
5pro	CTCTCCTGTTGCTGCTCTCCTGATAATA
6pro	CTCTCCTGTTGCTGCTCTCCTGATAATAC
7pro	CTCTCCTGTTGCTGCTCTCCTGATAATACG
8pro	CTCTCCTGTTGCTGCTCTCCTGATAATACGA
9pro	CTCTCCTGTTGCTGCTCTCCTGATAATACGAC
**10pro**	CTCTCCTGTTGCTGCTCTCCTGATAATACGACT
11pro	CTCTCCTGTTGCTGCTCTCCTGATAATACGACTC
12pro	CTCTCCTGTTGCTGCTCTCCTGATAATACGACTCA
13pro	CTCTCCTGTTGCTGCTCTCCTGATAATACGACTCAC
14pro	CTCTCCTGTTGCTGCTCTCCTGATAATACGACTCACT
15pro	CTCTCCTGTTGCTGCTCTCCTGATAATACGACTCACTA
16pro	CTCTCCTGTTGCTGCTCTCCTGATAATACGACTCACTAT
17pro	CTCTCCTGTTGCTGCTCTCCTGATAATACGACTCACTATA
pro4MB	GGGATTTGCGAATTTTTCAGGCTTTTTT
pro5MB	AGGGATTTGCGAATTTTTCAGGCTTTTTT
pro6MB	TAGGGATTTGCGAATTTTTCAGGCTTTTTT
pro7MB	ATAGGGATTTGCGAATTTTTCAGGCTTTTTT
pro8MB	TATAGGGATTTGCGAATTTTTCAGGCTTTTTT
pro9MB	CTATAGGGATTTGCGAATTTTTCAGGCTTTTTT
pro10MB	ACTATAGGGATTTGCGAATTTTTCAGGCTTTTTT
pro11MB	CACTATAGGGATTTGCGAATTTTTCAGGCTTTTTT
pro12MB	TCACTATAGGGATTTGCGAATTTTTCAGGCTTTTTT
pro13MB	CTCACTATAGGGATTTGCGAATTTTTCAGGCTTTTTT
pro14MB	ACTCACTATAGGGATTTGCGAATTTTTCAGGCTTTTTT
pro15MB	GACTCACTATAGGGATTTGCGAATTTTTCAGGCTTTTTT
pro16MB	CGACTCACTATAGGGATTTGCGAATTTTTCAGGCTTTTTT
pro17MB	ACGACTCACTATAGGGATTTGCGAATTTTTCAGGCTTTTTT

pro18MB	TACGACTCACTATAGGGATTTGCGAATTTTTCAGGCTTTTTT
pro19MB	ATACGACTCACTATAGGGATTTGCGAATTTTTCAGGCTTTTTT
pro20MB	AATACGACTCACTATAGGGATTTGCGAATTTTTCAGGCTTTTTT
pro21MB	TAATACGACTCACTATAGGGATTTGCGAATTTTTCAGGCTTTTTT
**pro15MB phos**	Phos-GACTCACTATAGGGATTTGCGAATTTTTCAGGCTTTTTT
**pro16MB phos**	Phos-CGACTCACTATAGGGATTTGCGAATTTTTCAGGCTTTTTT
**temp_pepper**	TCCCGGCGCCAGTGCCTGCCGAAGCAGGCCGACACGCCACGATTGGGGATCCCTA TAGTGAGTCGTATTATCAGGAGAGCAGCAACAGGAGAG
**pos_pepper**	CTCTCCTGTTGCTGCTCTCCTGATAATACGACTCACTATAGGGATCCCCAATCGTGG CGTGTCGGCCTGCTTCGGCAGGCACTGGCGCCGGGA
**pro12_pepper**	TCACTATAGGGATCCCCAATCGTGGCGTGTCGGCCTGCTTCGGCAGGCACTGGCGC CGGGA
**pro11_pepper**	CACTATAGGGATCCCCAATCGTGGCGTGTCGGCCTGCTTCGGCAGGCACTGGCGCC GGGA
**pro4_pepper**	GGGATCCCCAATCGTGGCGTGTCGGCCTGCTTCGGCAGGCACTGGCGCCGGGA
**Pepper reference**	UCCCCAAUCGUGGCGUGUCGGCCUGCUUCGGCAGGCACUGGCGCCGGGA
**Pepper marker (R)**	GGGAUCCCCAAUCGUGGCGUGUCGGCCUGCUUCGGCAGGCACUGGCGCCGGGA
**Temp scrambled**	AAAAAAGCCTGAAAAATTCGCAAACAGTATGGCTTATCTACGACTTCAGGAGAGCAG CAACAGGAGAG
Pro21_MB_scrambled	AGTCGTAGATAAGCCATACTGTTTGCGAATTTTTCAGGCTTTTTT
**Molecular Beacon (MB)**	FAM-UoUoUoGoCoCoUoGoAoAoAoAoAoUoUoCoGoCoAoAoAo-DABCYL
**Switch Template**	AAAAAAGCCTGAAAAATTCGCAAATCCCTATAGTGAGTCGTATTATTTTTTTTTTTTTTT TCCACTGGAAGAAGCAGGTAC
**Switch HP**	GTACCTGCTTCTTCCAGTGGTCAGGAGAGCAGCAACAGGAGAGGTCTTAATACGACC TCTCCTGTTGCTGC
**Input RNA**	GACCUCUCCUGUUGCUGCUCUCCUG
Switch Pos	GTACCTGCTTCTTCCAGTGGTCAGGAGAGCAGCAACAGGAGAGGTCTTAATACGAC

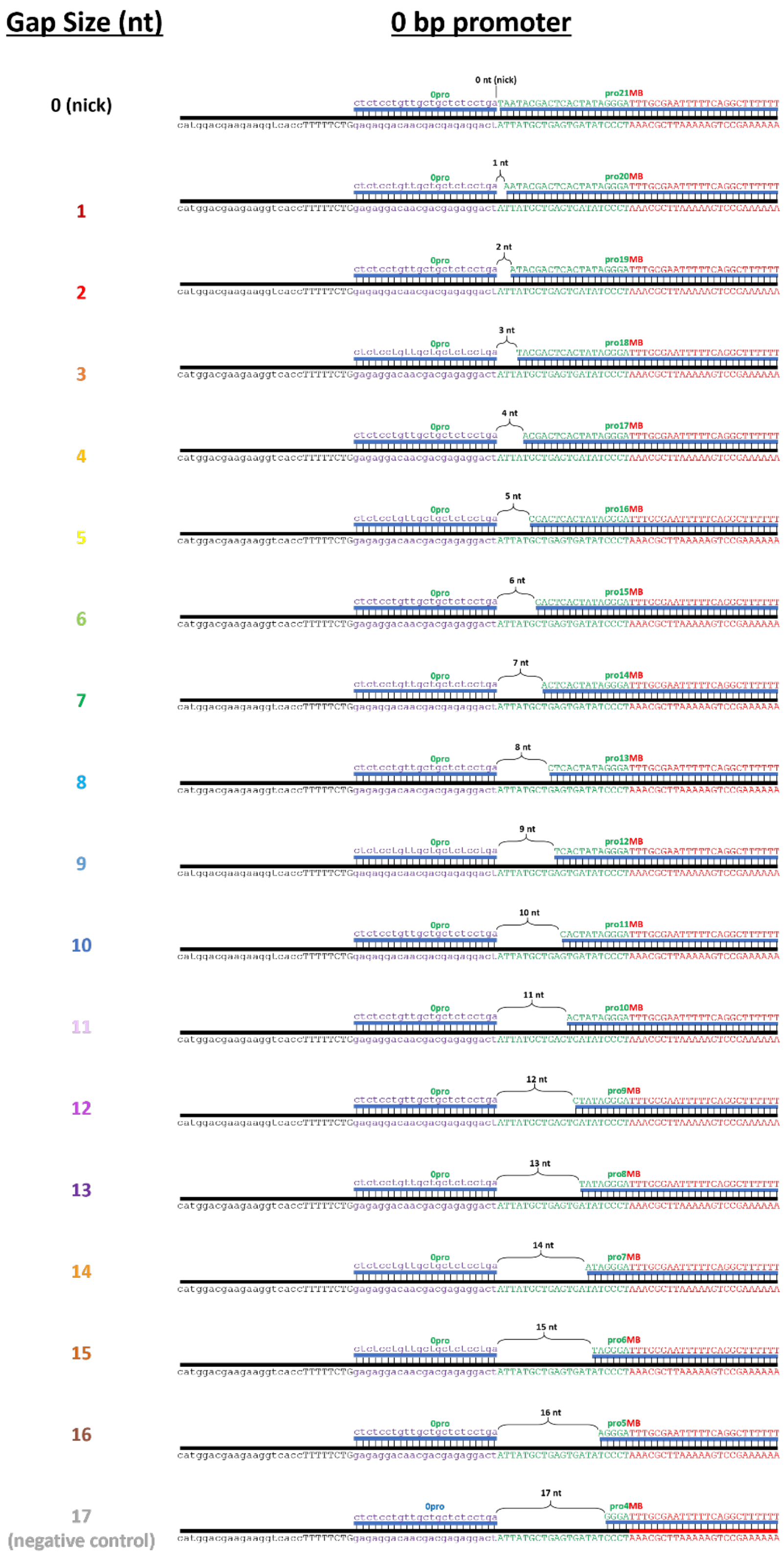

Figure S1: Schematic for 0 bp promoter structures with 0-17 nt gaps. Template strands are black, coding strands are blue, and sequence text is colored based on functional region, purple = handle, green = T7 promoter, red = MB target region. Gap size names on left are colored to correlate with the curve in raw data graphs (Figure S15) that correspond to that structure.

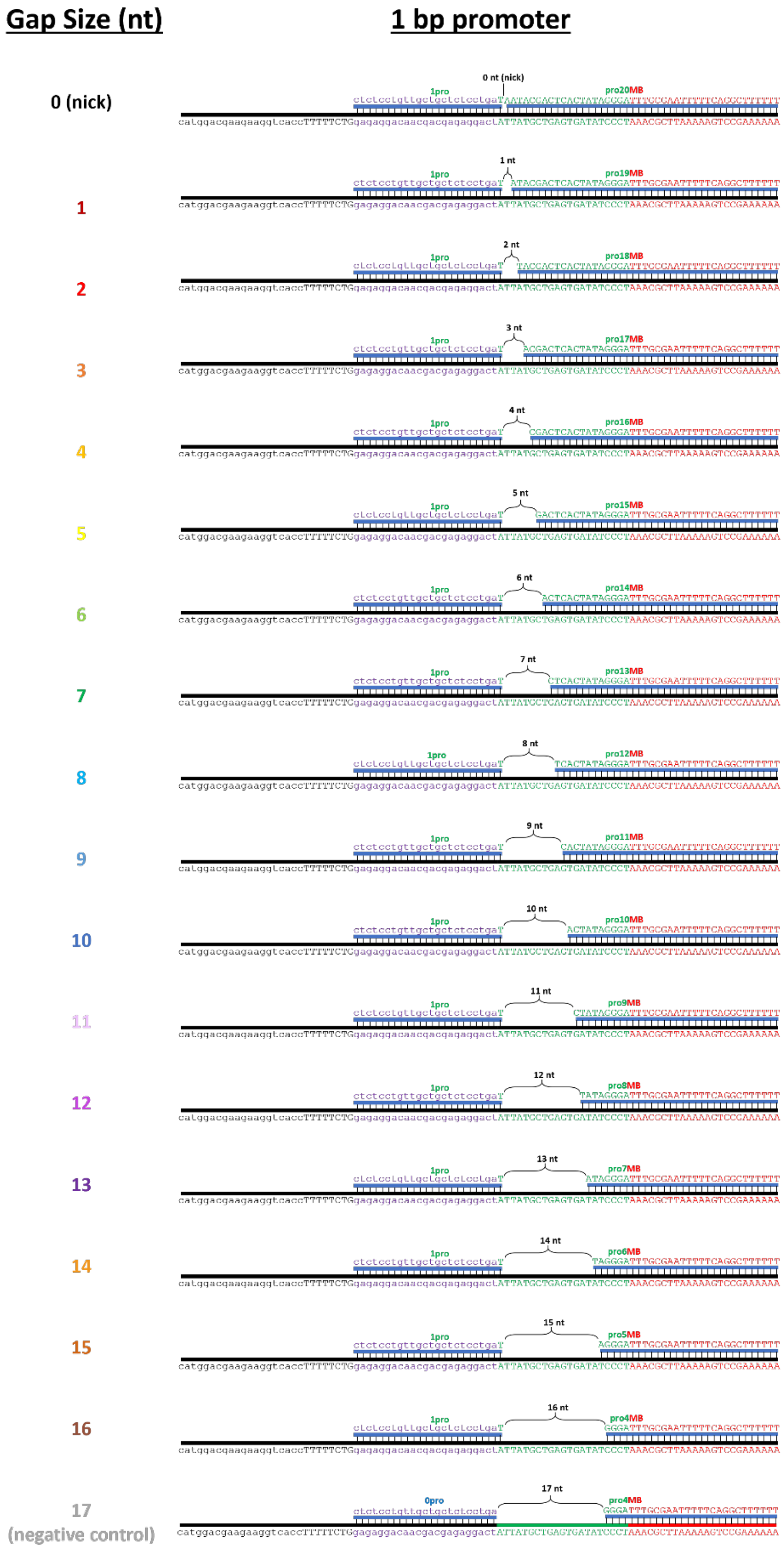

Figure S2: Schematic for 1 bp promoter structures with 0-17 nt gaps. Template strands are black, coding strands are blue, and sequence text is colored based on functional region, purple = handle, green = T7 promoter, red = MB target region. Gap size names on left are colored to correlate with the curve in raw data graphs (Figure S16) that correspond to that structure.

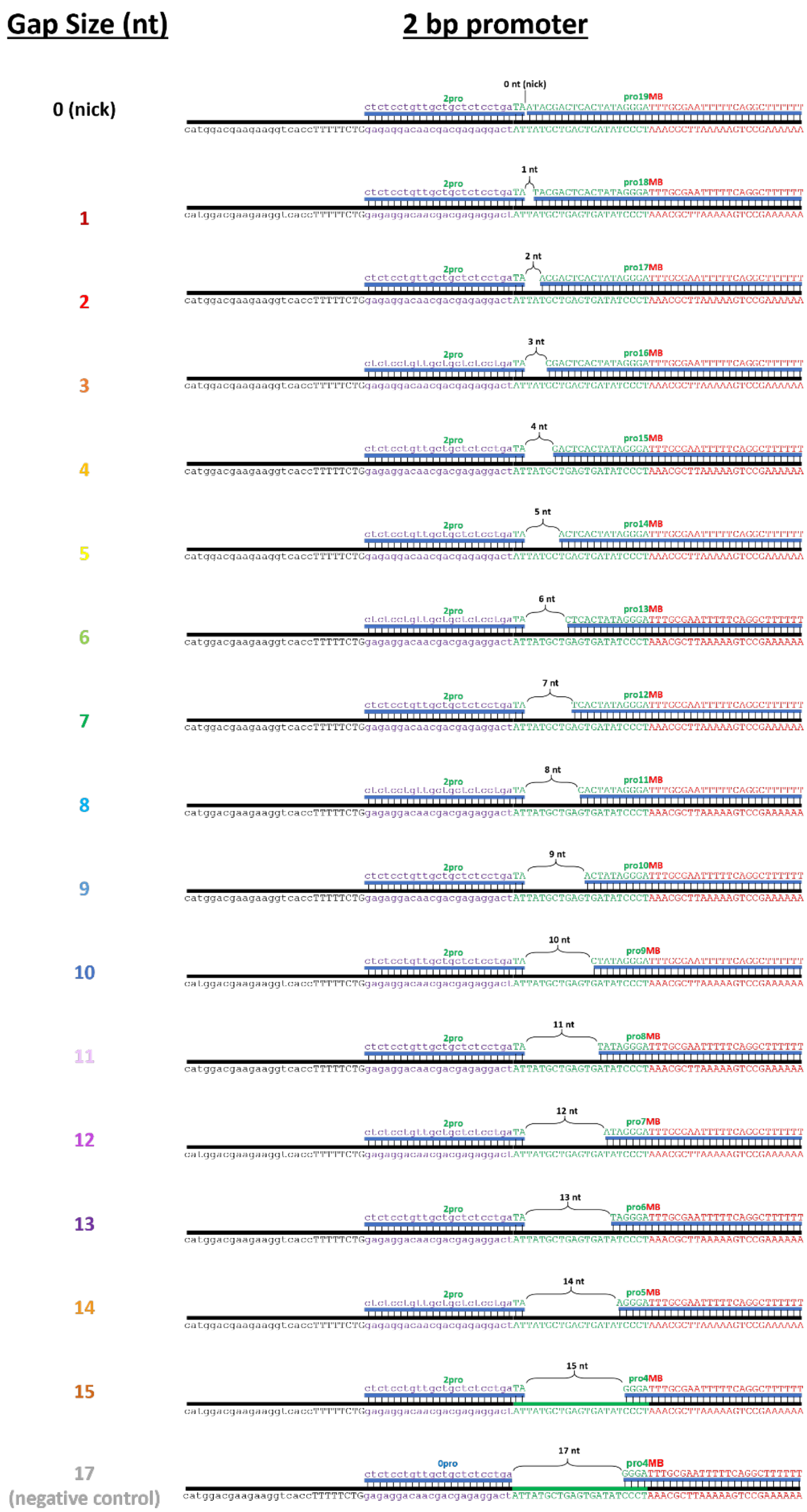

Figure S3: Schematic for 2 bp promoter structures with 0-15, and 17 nt gaps. Template strands are black, coding strands are blue, and sequence text is colored based on functional region, purple = handle, green = T7 promoter, red = MB target region. Gap size names on left are colored to correlate with the curve in raw data graphs (Figure S17) that correspond to that structure.

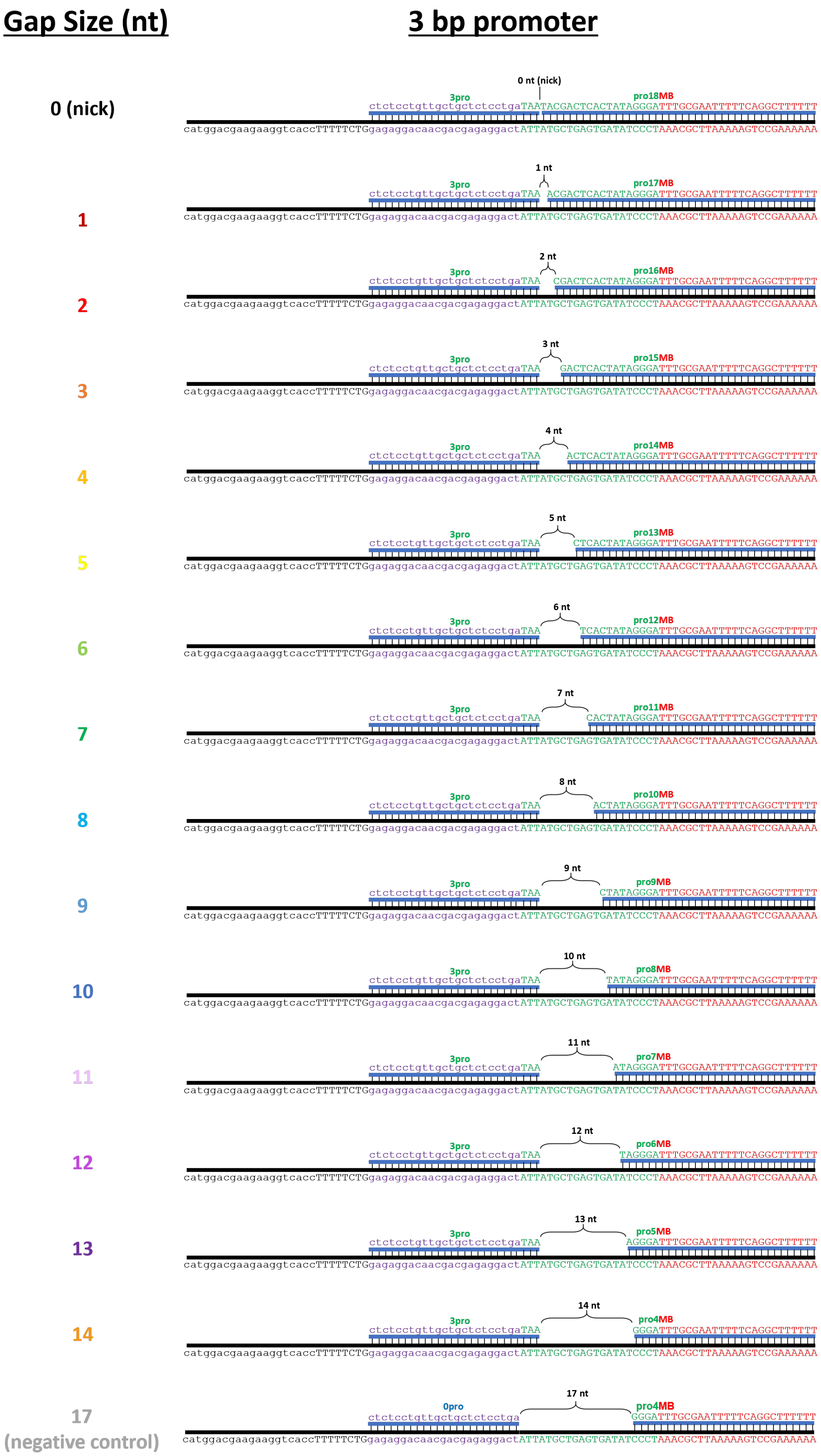

Figure S4: Schematic for 3 bp promoter structures with 0-14, and 17 nt gaps. Template strands are black, coding strands are blue, and sequence text is colored based on functional region, purple = handle, green = T7 promoter, red = MB target region. Gap size names on left are colored to correlate with the curve in raw data graphs (Figure S18) that correspond to that structure.

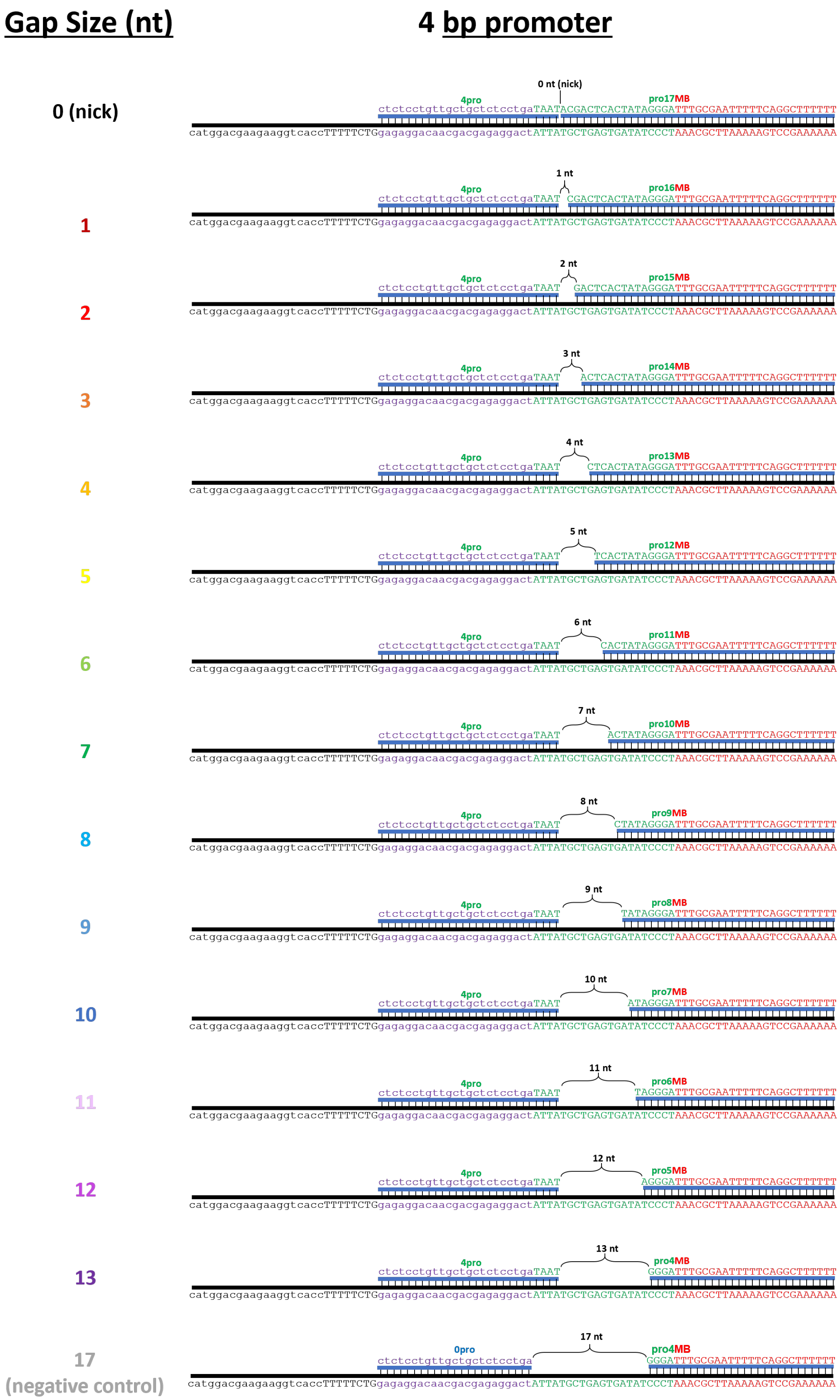

Figure S5: Schematic for 4 bp promoter structures with 0-13, and 17 nt gaps. Template strands are black, coding strands are blue, and sequence text is colored based on functional region, purple = handle, green = T7 promoter, red = MB target region. Gap size names on left are colored to correlate with the curve in raw data graphs (Figure S19) that correspond to that structure.

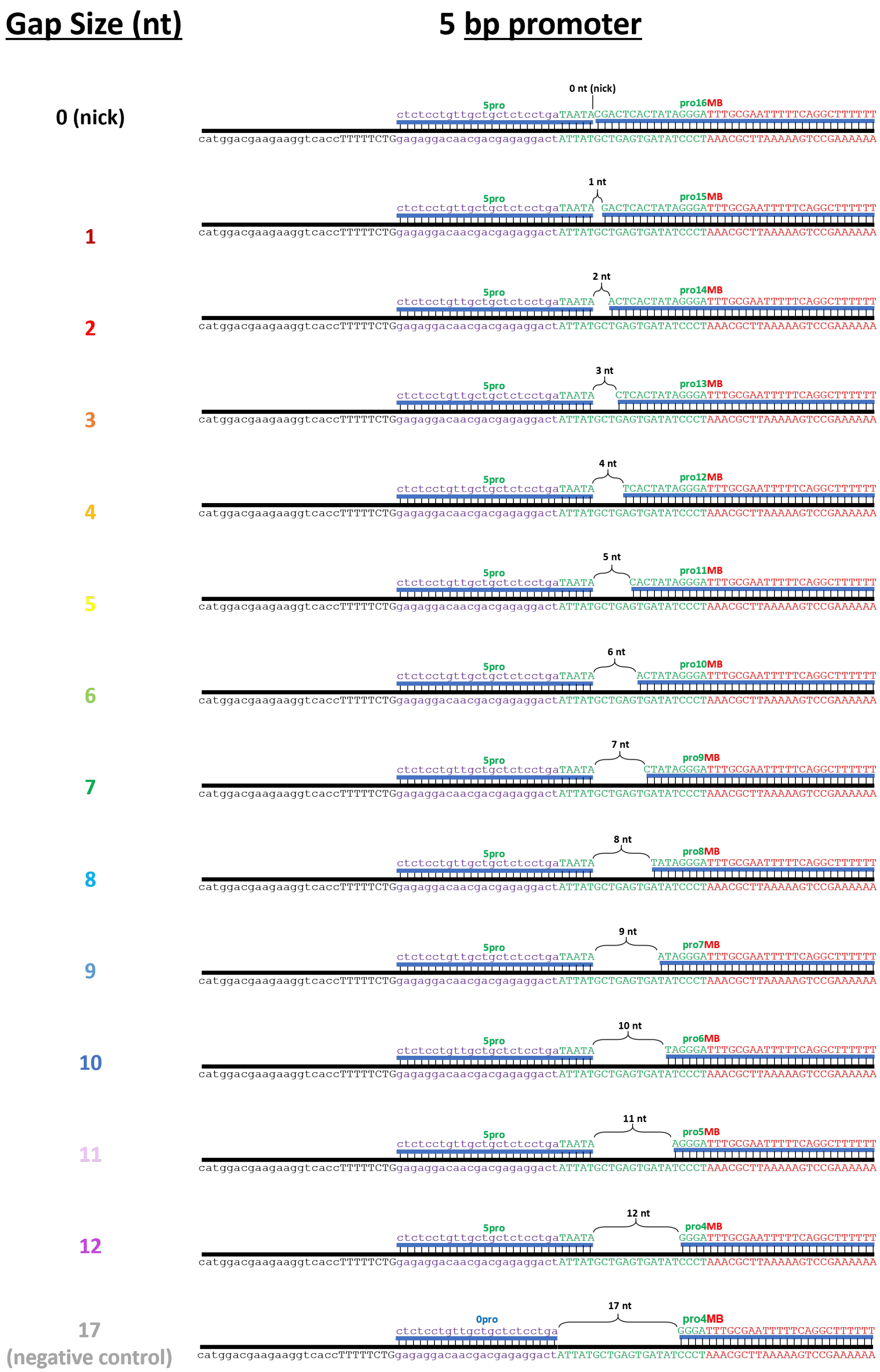

Figure S6: Schematic for 5 bp promoter structures with 0-12, and 17 nt gaps. Template strands are black, coding strands are blue, and sequence text is colored based on functional region, purple = handle, green = T7 promoter, red = MB target region. Gap size names on left are colored to correlate with the curve in raw data graphs (Figure S20) that correspond to that structure

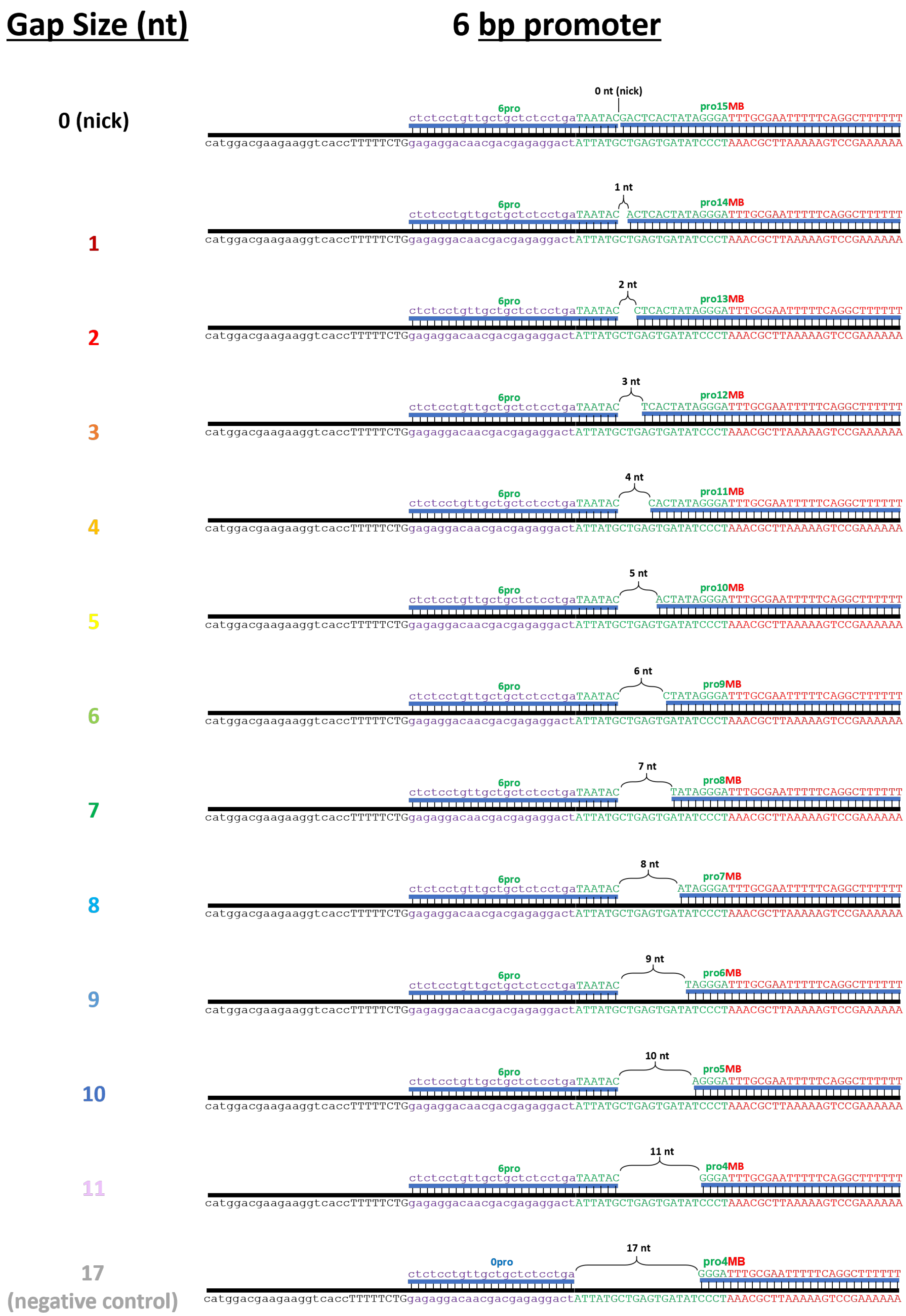

Figure S7: Schematic for 6 bp promoter structures with 0-11, and 17 nt gaps. Template strands are black, coding strands are blue, and sequence text is colored based on functional region, purple = handle, green = T7 promoter, red = MB target region. Gap size names on left are colored to correlate with the curve in raw data graphs (Figure S21) that correspond to that structure.

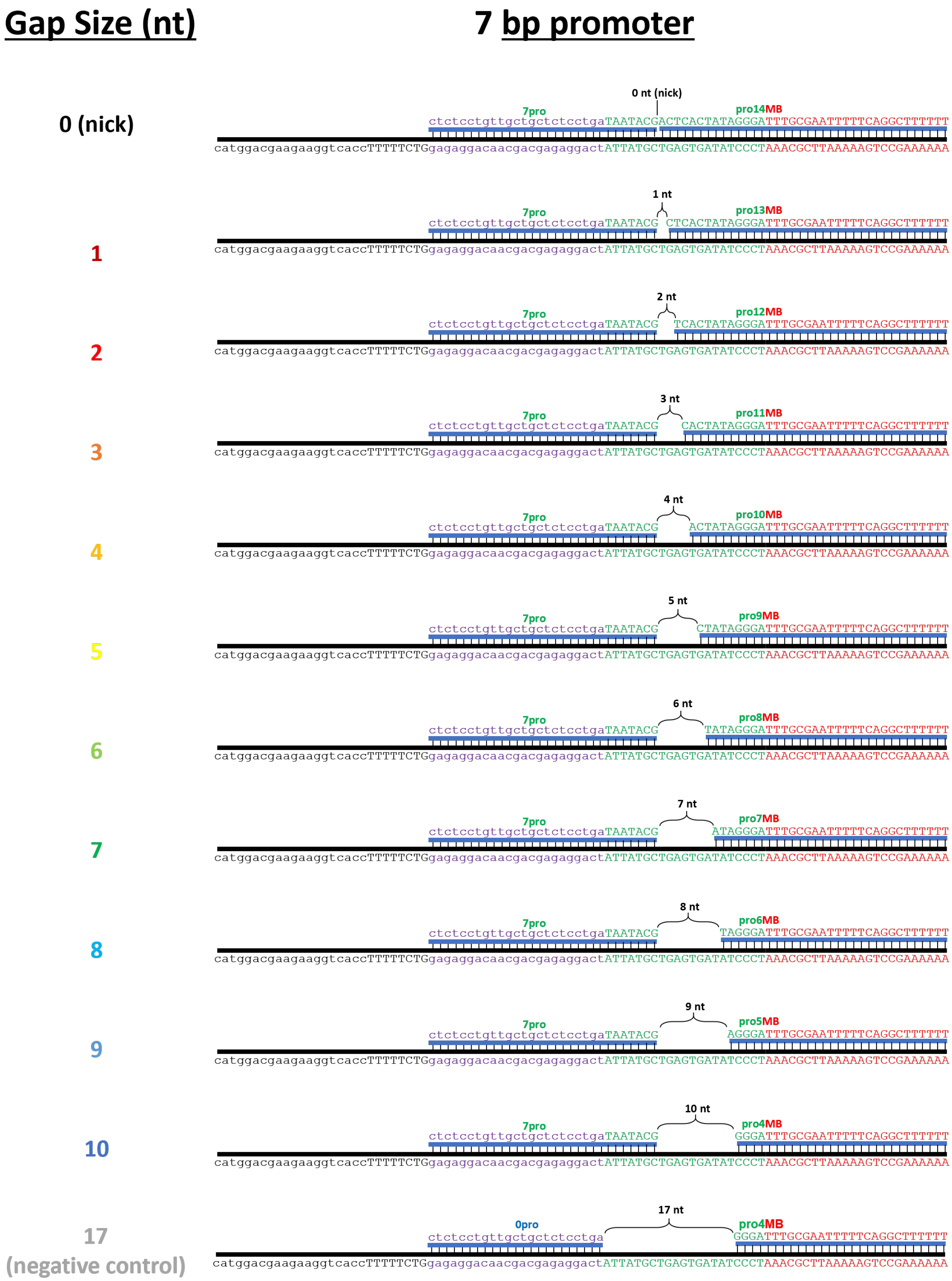

Figure S8: Schematic for 7 bp promoter structures with 0-10, and 17 nt gaps. Template strands are black, coding strands are blue, and sequence text is colored based on functional region, purple = handle, green = T7 promoter, red = MB target region. Gap size names on left are colored to correlate with the curve in raw data graphs (Figure S22) that correspond to that structure.

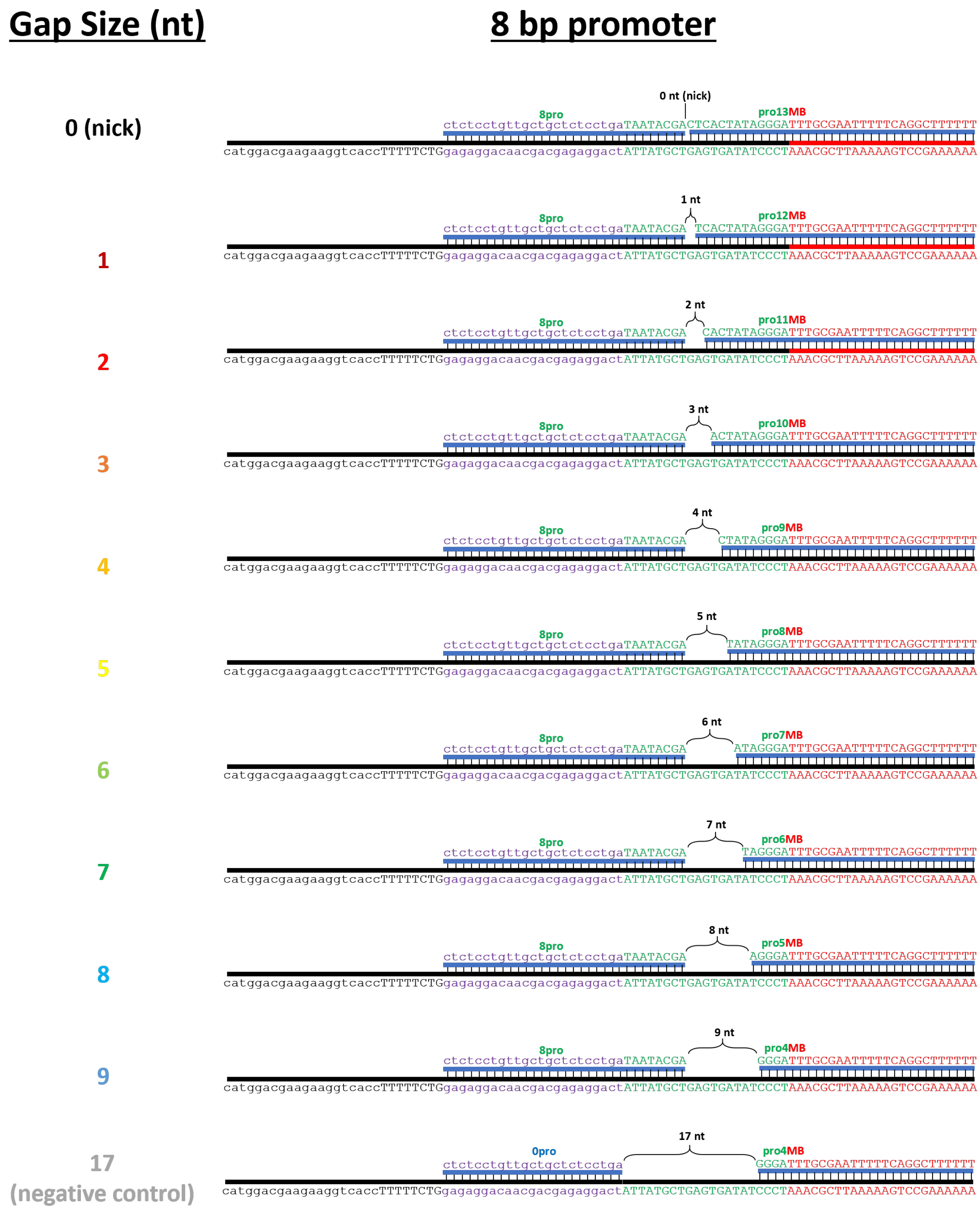

Figure S9: Schematic for 8 bp promoter structures with 0-9 and 17 nt gaps. Template strands are black, coding strands are blue, and sequence text is colored based on functional region, purple = handle, green = T7 promoter, red = MB target region. Gap size names on left are colored to correlate with the curve in raw data graphs (Figure S23) that correspond to that structure.

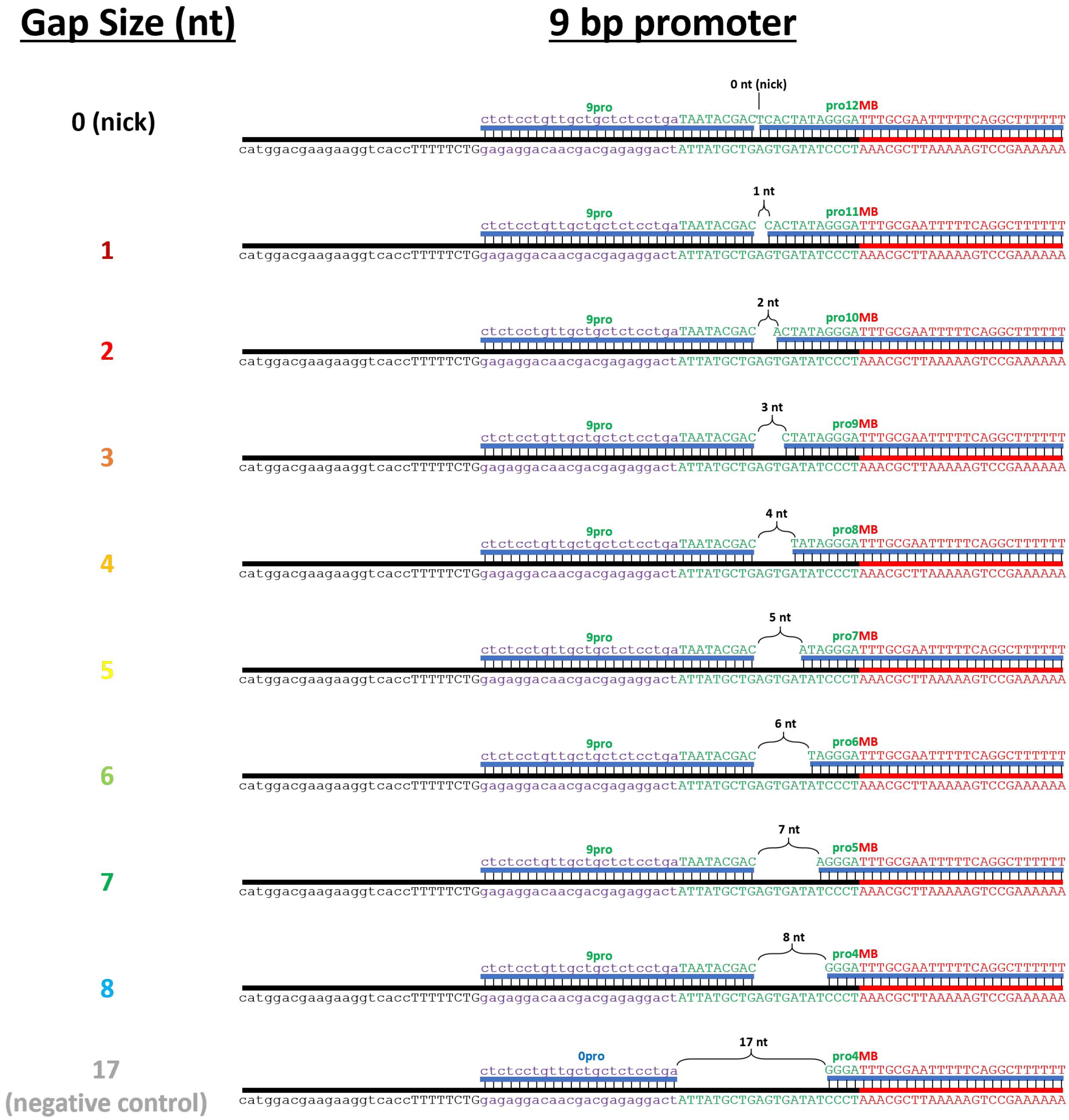

Figure S10: Schematic for 9 bp promoter structures with 0-8, and 17 nt gaps. Template strands are black, coding strands are blue, and sequence text is colored based on functional region, purple = handle, green = T7 promoter, red = MB target region. Gap size names on left are colored to correlate with the curve in raw data graphs (Figure S24) that correspond to that structure.

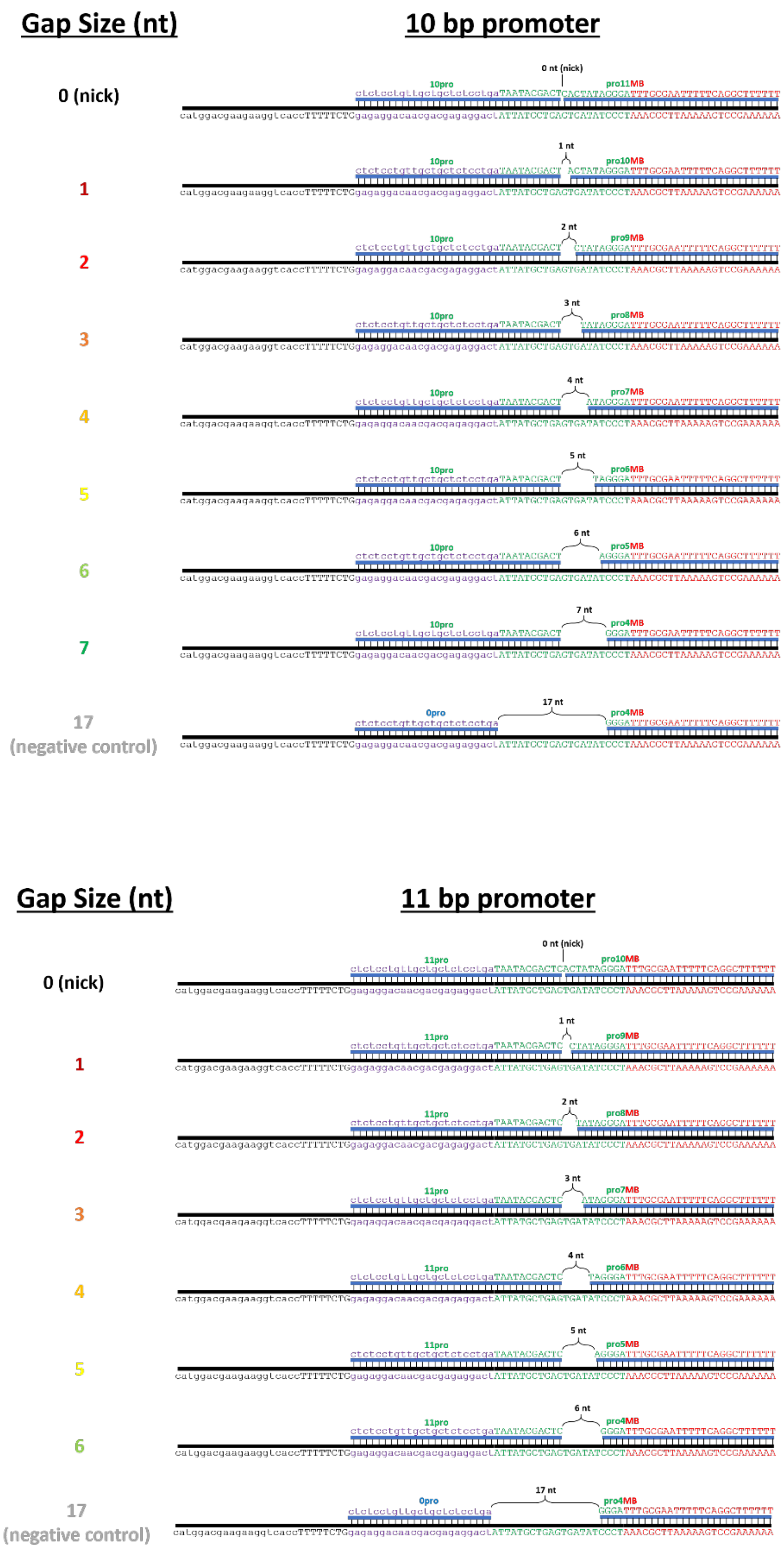

Figure S11: Schematic for 10 and 11 bp promoter structures with 0-7, and 17 nt gaps or 0-6, and 17 nt gaps respectively. Template strands are black, coding strands are blue, and sequence text is colored based on functional region, purple = handle, green = T7 promoter, red = MB target region. Gap size names on left are colored to correlate with the curve in raw data graphs (Figures S25 and S26) that correspond to that structure.

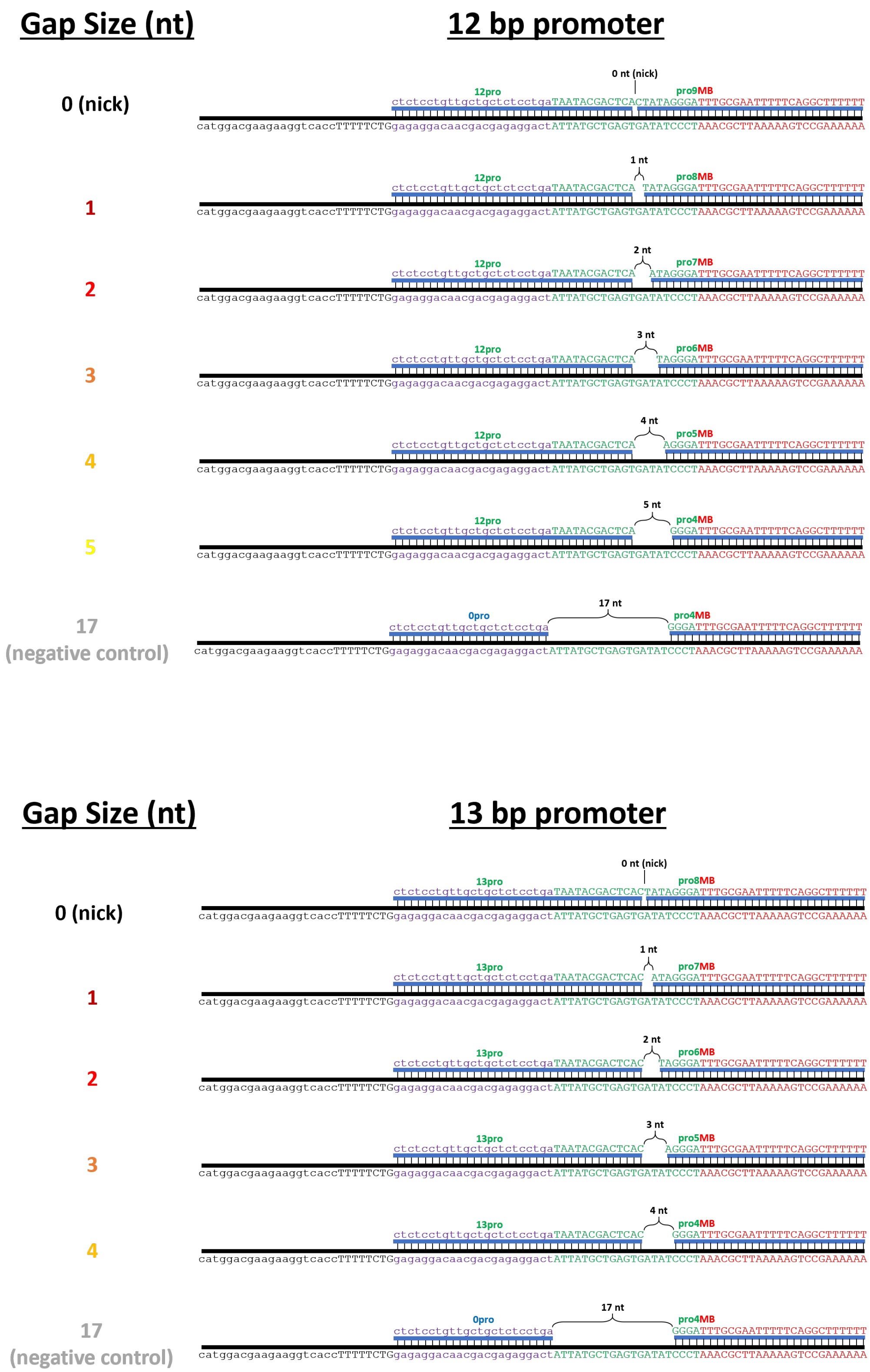

Figure S12: Schematic for 12 and 13 bp promoter structures with 0-5, and 17 nt gaps or 0-4, and 17 nt gaps respectively. Template strands are black, coding strands are blue, and sequence text is colored based on functional region, purple = handle, green = T7 promoter, red = MB target region. Gap size names on left are colored to correlate with the curve in raw data graphs (Figures S27 and S28) that correspond to that structure.

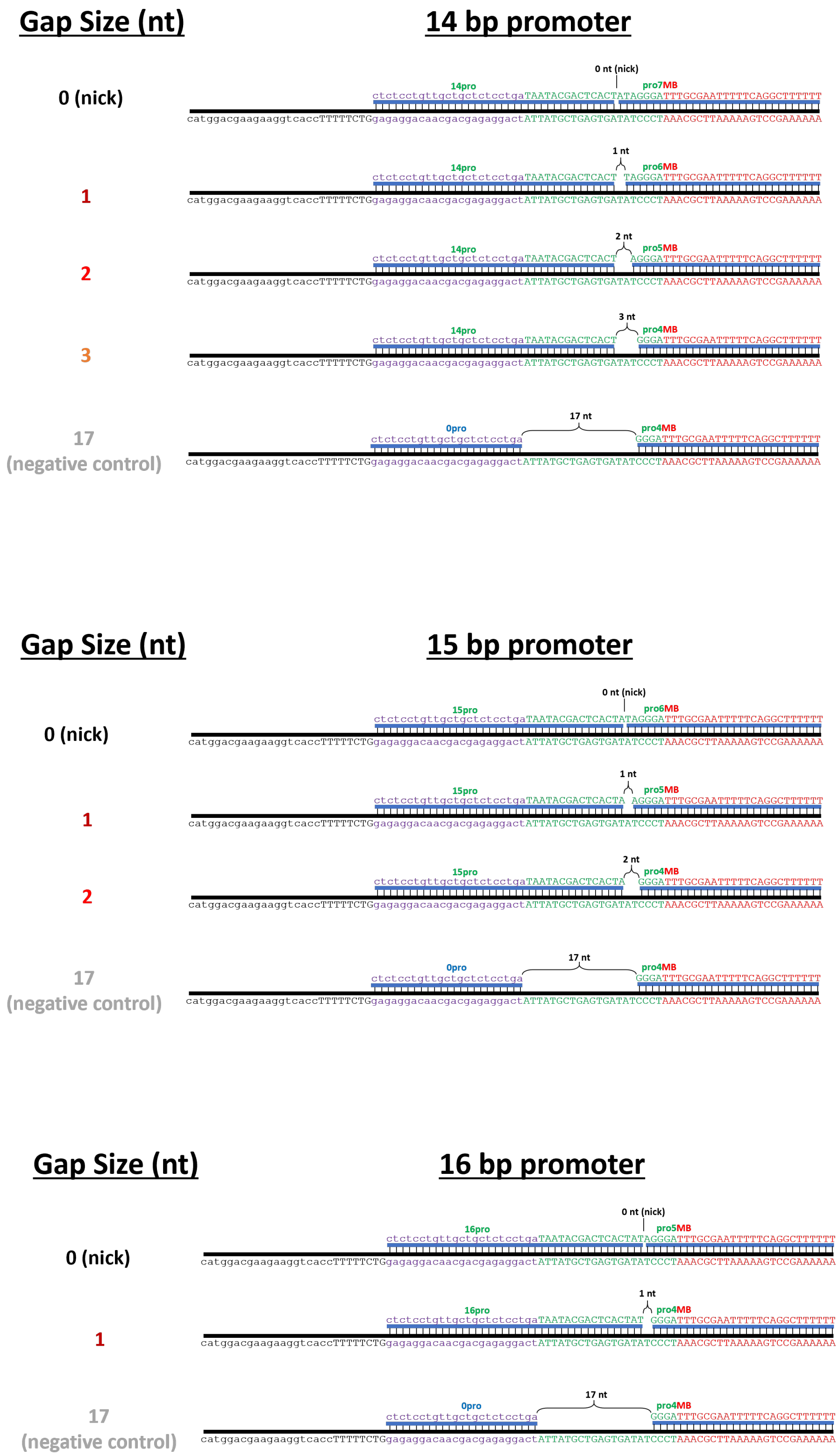

Figure S13: Schematic for 14, 15, and 16 bp promoter structures with 0-3 and 17 nt gaps, 0-2 and 17 nt gaps, or 0-1 and 17 nt gaps. respectively. Template strands are black, coding strands are blue, and sequence text is colored based on functional region, purple = handle, green = T7 promoter, red = MB target region. Gap size names on left are colored to correlate with the curve in raw data graphs (Figures S29-31) that correspond to that structure.

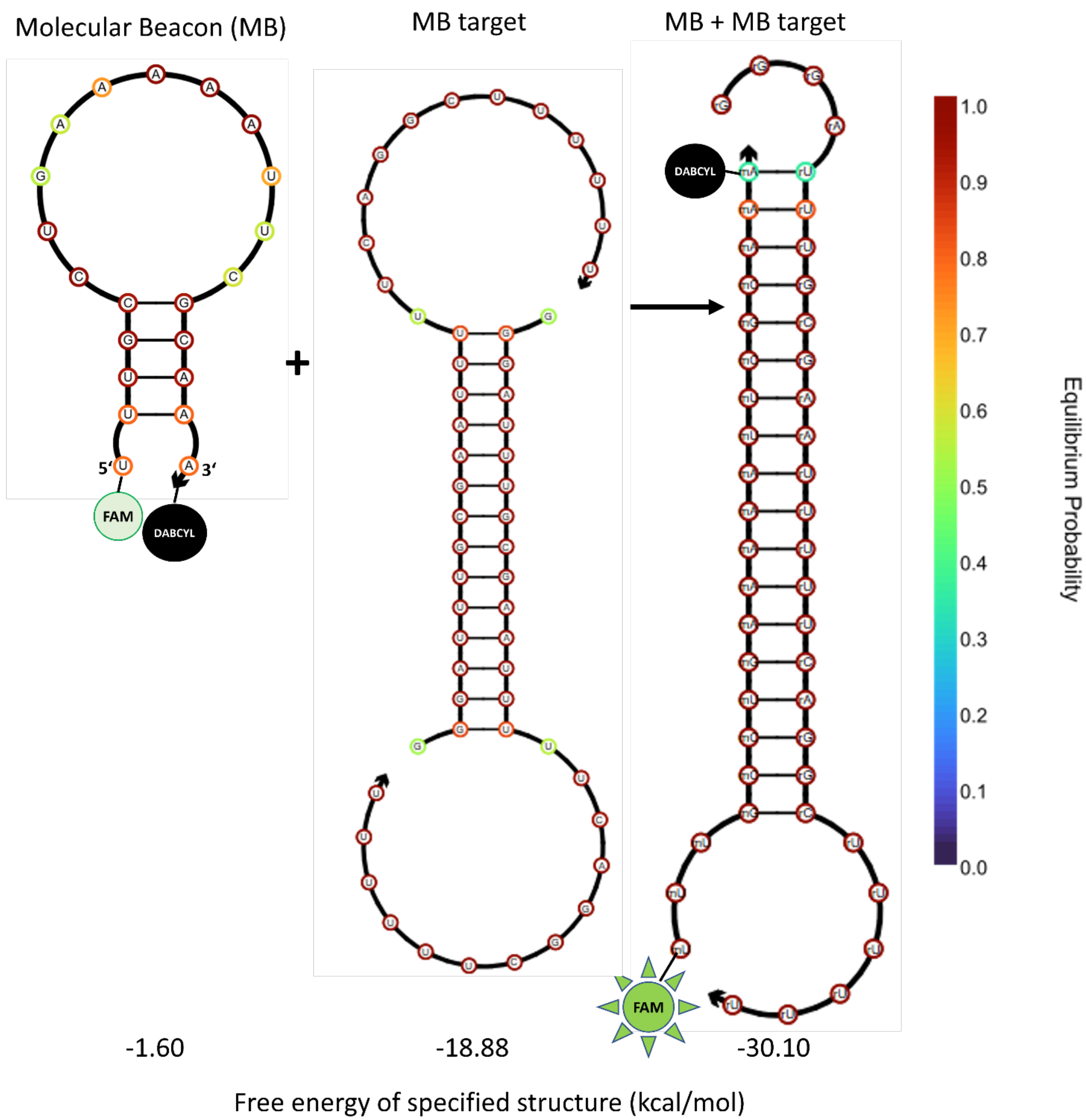

Figure S14: Nupack[1-3] folding prediction for the molecular beacon (MB, left), MB target transcription product (middle), and the MB hybridized to the MB target (right) based on 2' OMe RNA model, RNA model, and 2'OMe/RNA hybrid model respectively in .012 M NaCl at 30°C for complexes containing 2 strands. Base color shading represents the probability of hybridization with red being more likely and purple being less likely, scale bar to right. The free energy of the specified structures is given below each image in kcal/mol. All bases of the MB are 2' OMe modified RNA and it is 5' modified with a 6FAM fluorophore and 3' modified with a DABCYL quencher. The MB forms a hairpin that brings the 5' and 3' ends together resulting in quenching of the FAM fluorophore. Upon hybridization to the target RNA sequence, the MB hairpin is opened which results in separation of the FAM from the DABCYL quencher and therefore fluorescence intensity increases. Such a method can be used to track transcription progress.

.

Supplementary Text ST1: concerning 0,1, and 2 bp promoter structures

As mentioned in the main text, 0,1, and 2 bp promoters with small gaps exhibit different behavior than the other structures. Notably 2 bp promoter with a 1 nt gap at position -15 show marginal transcription activity, whereas larger gaps abolish transcription. In 1 bp promoters, a 1 nt gap at position -16 allows detectable transcription, albeit at a rate approximately 50% that of the nicked sample. Larger gaps again eliminate transcription. For 0 bp promoters, transcription is only observed, albeit at very low levels, when a 1 nt gap is placed at position -17 or a 2 nt gap spans positions -17 and -16; all larger gaps are nonfunctional (Supplementary data file, Figures S15-S17). This behavior is likely due to the fact that most of the promoter is double-stranded and is in accordance with previously cited work.[4]

Supplementary Text ST1.1: concerning the large variations in transcription rate

The following Figures (Figures S15-S31) show the raw data on which the conclusion was based that small gaps in the non-template promoter prevent transcription while larger gaps recover transcription, as well as the normalized transcription rates for the # bp promoter structures shown in the schematics in Figures S1-S14. The large error bars arise when we attempt to quantify this effect in order to represent many experiments in a single figure. The linear range normalized to the negative control proved to be the best method for this (heat map, Figure 1C), although this results in high standard deviations. Despite the high standard deviations, T-tests presented in the figures below still yield significant results.

The large error bars for some of the transcription rates are likely due to two sources, 1) the multi-channel pipette and 2) variations in each aliquot of our in-house-prepared T7 RNA polymerase.

1) We used a multi-channel pipette to add the DNA substrates to the reaction mixture in order to ensure that transcription starts at approximately the same time for each sample. There will be some variation in the volume taken up by each channel.

2) Due to the number of samples involved the molecular beacon experiments required extremely large volumes of transcription buffer. Consequently, we used up many aliquots of our self-prepared T7 RNA polymerase and other reagents. Although we prepared the transcription buffer in exactly the same way each time, unavoidable differences between aliquots are likely the cause of such variations in the transcription rate during the linear phase.

However, the general trend (that small gaps in the non-template promoter prevent transcription while larger gaps restore transcription) remains consistent despite the fluctuations in transcription rate, is clearly evident in the raw data and is the reason why we are including the raw data here.

At the start of each run, the FAM signal in the negative control initially appears to rise. This is likely due to 3 reasons: First, and most likely, some molecular beacons (MB) may melt due to the rise in temperature. Second, although the template strand is present at 1.5-fold excess relative to the 3' coding strand, there will still be a certain proportion of unbound 3' coding strand (which has a region complementary to the MB) that can hybridize and open the MB thereby increasing FAM fluorescence. Finally, as mentioned in the manuscript's conclusion, there could be some promoter independent transcription[5-6], which cannot be ruled out.

Note: Some of the curves in the raw data show a profile in which the transcription rate levels off and then rises. We suspect that this is an artifact resulting from the use of a PCR cycler to measure the increase in the fluorescence signal. Since these profiles appear only late in the course of the reaction (after it has run its course), they do not affect the conclusions of this work.

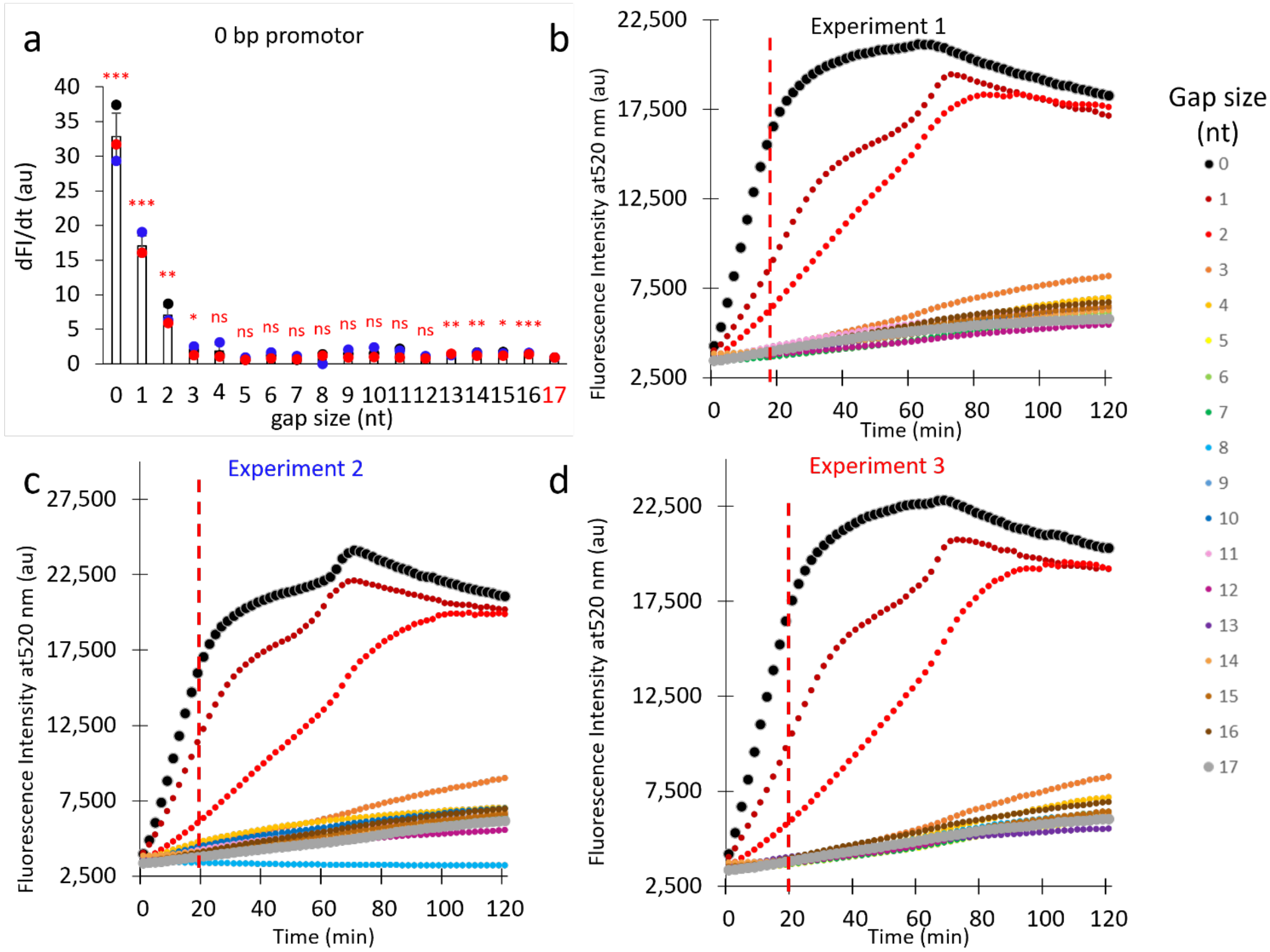

Figure S15: FAM molecular beacon fluorescence intensity transcription tracking for 0 bp promoter structure with gaps of various sizes. a) fluorescence increase during the linear phase, normalized to the 17 nt gap negative control (17), n=3 (black, blue, red dots), bar graphs are mean, error bars are s.d. Red significance marks show one tailed homoscedastic T-tests in relation to the negative control, highlighted red; ns: p>.05, *: p<.05, **: p<.01, ***: p<.001. Linear phase is denoted by red dashed lines in b) raw data for the first experimental repeat (black dots in a), c) raw data for the second experimental repeat (blue dots in a), and d) raw data for the third experimental repeat (red dots in a). Legend to right shows what color line in b-d correlates to what gap size. These colors also correlate to the gap size color in Figure S1.

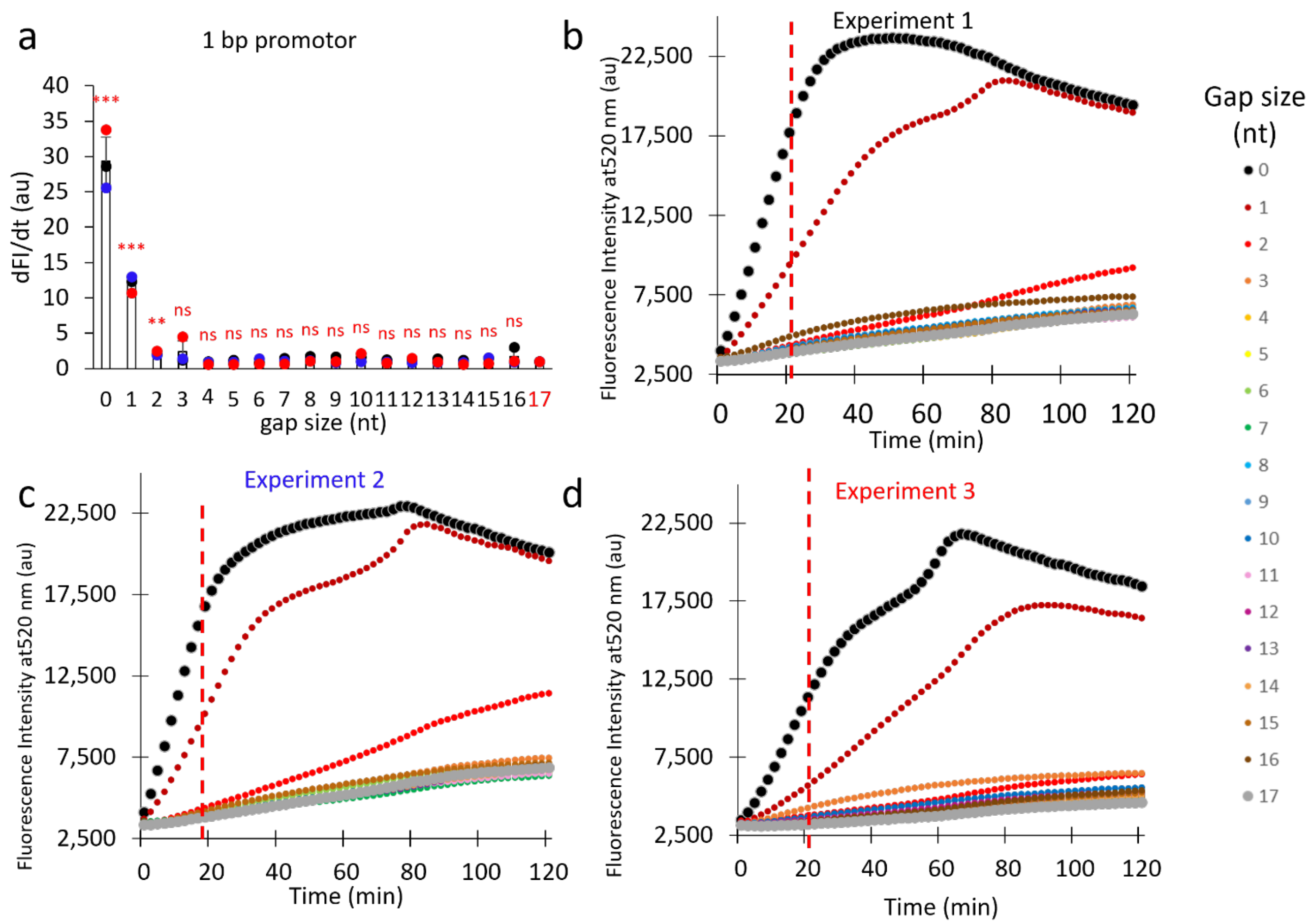

Figure S16: FAM molecular beacon fluorescence intensity transcription tracking for 1 bp promoter structure with gaps of various sizes. a) fluorescence increase during the linear phase, normalized to the 17 nt gap negative control (17), n=3 (black, blue, red dots), bar graphs are mean, error bars are s.d. Red significance marks show one tailed homoscedastic T-tests in relation to the negative control, highlighted red; ns: p>.05, **: p<.01, ***: p<.001. Linear phase is denoted by red dashed lines in b) raw data for the first experimental repeat (black dots in a), c) raw data for the second experimental repeat (blue dots in a), and d) raw data for the third experimental repeat (red dots in a). Legend to right shows what color line in b-d correlates to what gap size. These colors also correlate to the gap size color in Figure S2.

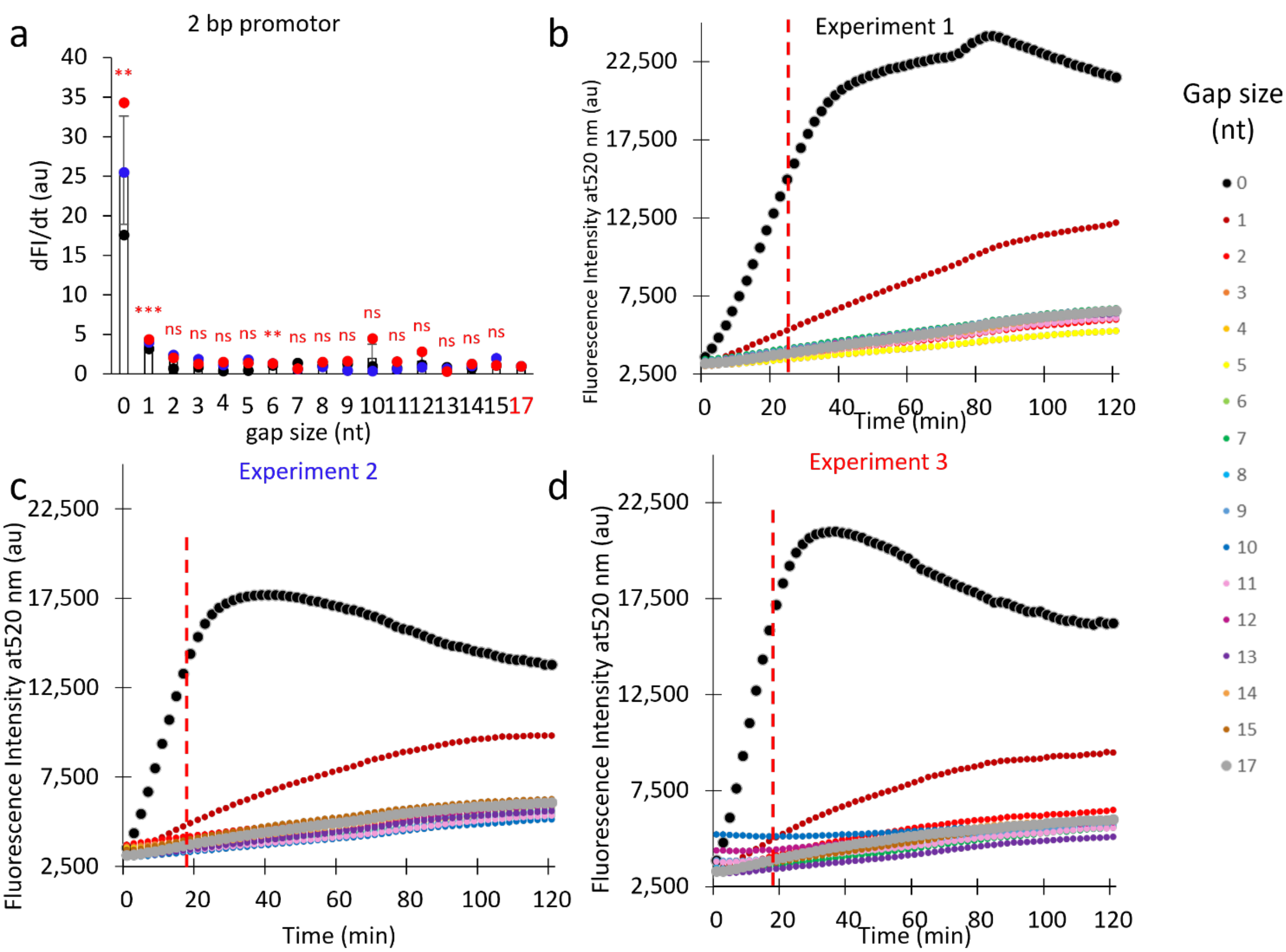

Figure S17: FAM molecular beacon fluorescence intensity transcription tracking for 2 bp promoter structure with gaps of various sizes. a) fluorescence increase during the linear phase, normalized to the 17 nt gap negative control (17), n=3 (black, blue, red dots), bar graphs are mean, error bars are s.d. Red significance marks show one tailed homoscedastic T-tests in relation to the negative control, highlighted red; ns: p>.05, **: p<.01, ***: p<.001. Linear phase is denoted by red dashed lines in b) raw data for the first experimental repeat (black dots in a), c) raw data for the second experimental repeat (blue dots in a), and d) raw data for the third experimental repeat (red dots in a). Legend to right shows what color line in b-d correlates to what gap size. These colors also correlate to the gap size color in Figure S3.

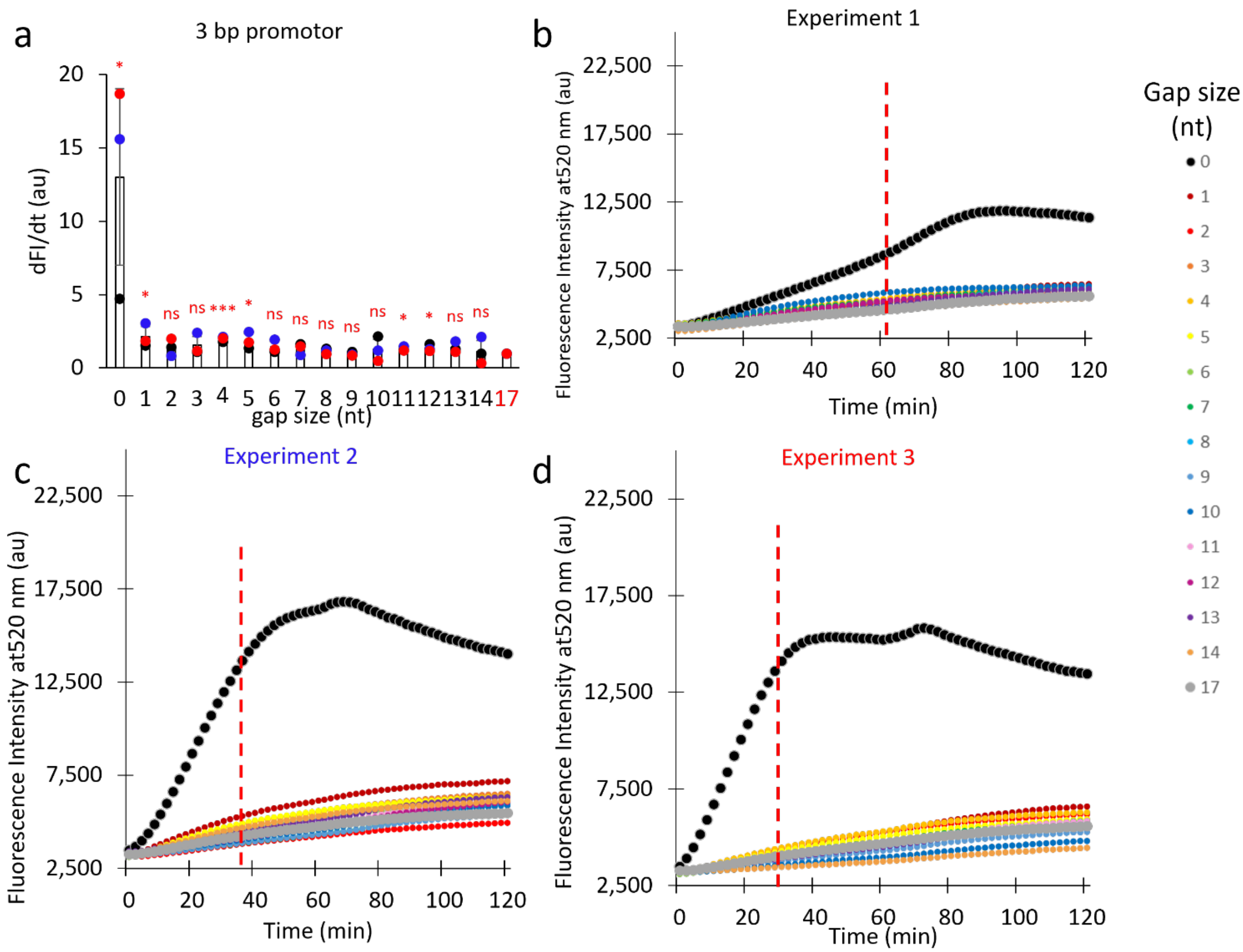

Figure S18: FAM molecular beacon fluorescence intensity transcription tracking for 3 bp promoter structure with gaps of various sizes. a) fluorescence increase during the linear phase, normalized to the 17 nt gap negative control (17), n=3 (black, blue, red dots), bar graphs are mean, error bars are s.d. Red significance marks show one tailed homoscedastic T-tests in relation to the negative control, highlighted red; ns: p>.05, *: p<.05, ***: p<.001. Linear phase is denoted by red dashed lines in b) raw data for the first experimental repeat (black dots in a), c) raw data for the second experimental repeat (blue dots in a), and d) raw data for the third experimental repeat (red dots in a). Legend to right shows what color line in b-d correlates to what gap size. These colors also correlate to the gap size color in Figure S4.

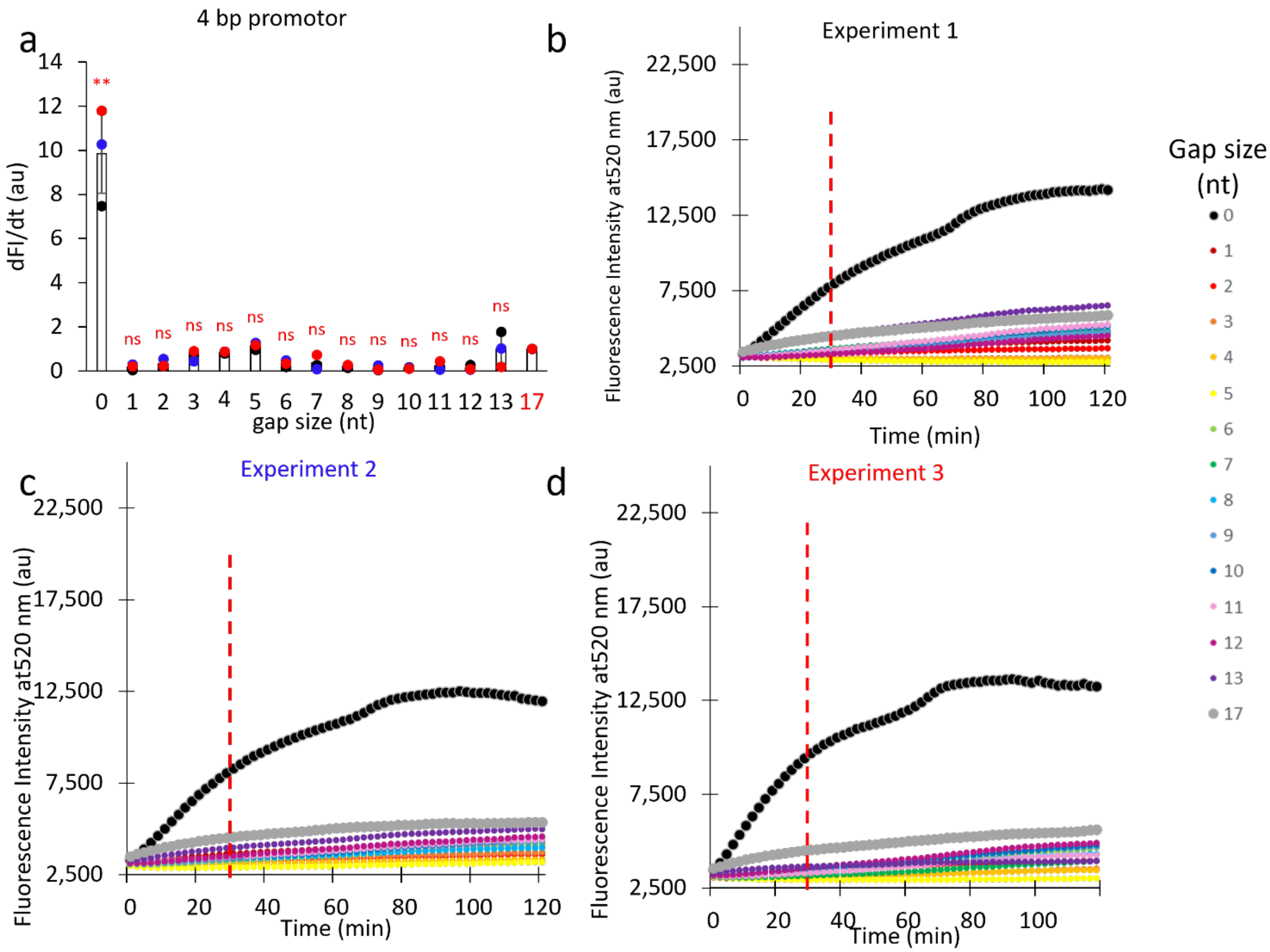

Figure S19: FAM molecular beacon fluorescence intensity transcription tracking for 4 bp promoter structure with gaps of various sizes. a) fluorescence increase during the linear phase, normalized to the 17 nt gap negative control (17), n=3 (black, blue, red dots), bar graphs are mean, error bars are s.d. Red significance marks show one tailed homoscedastic T-tests in relation to the negative control, highlighted red; ns: p>.05, **: p<.01. Linear phase is denoted by red dashed lines in b) raw data for the first experimental repeat (black dots in a), c) raw data for the second experimental repeat (blue dots in a), and d) raw data for the third experimental repeat (red dots in a). Legend to right shows what color line in b-d correlates to what gap size. These colors also correlate to the gap size color in Figure S5.

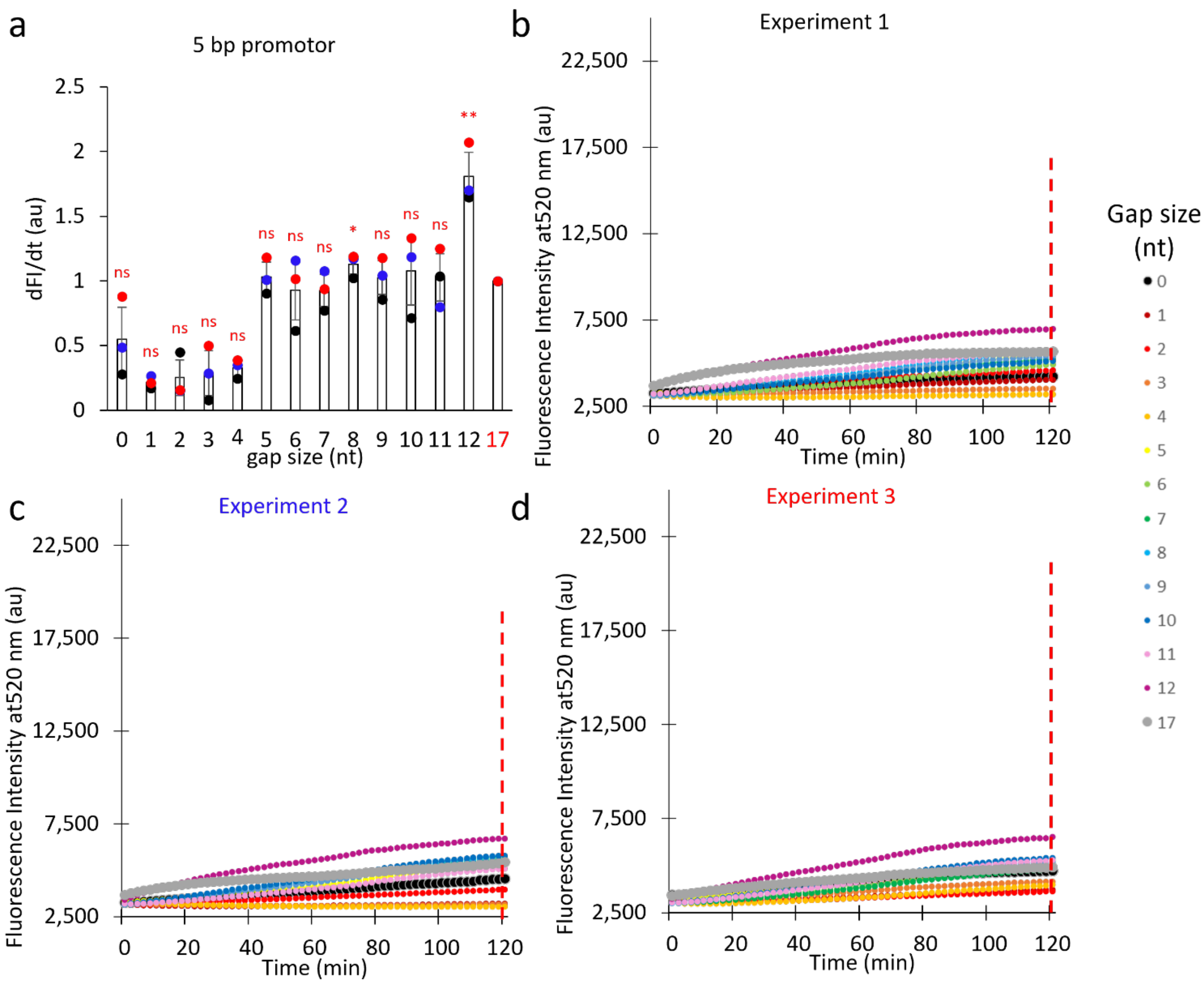

Figure S20: FAM molecular beacon fluorescence intensity transcription tracking for 5 bp promoter structure with gaps of various sizes. a) fluorescence increase during the linear phase, normalized to the 17 nt gap negative control (17), n=3 (black, blue, red dots), bar graphs are mean, error bars are s.d. Red significance marks show one tailed homoscedastic T-tests in relation to the negative control, highlighted red; ns: p>.05, *: p<.05, **: p<.01. Linear phase is denoted by red dashed lines in b) raw data for the first experimental repeat (black dots in a), c) raw data for the second experimental repeat (blue dots in a), and d) raw data for the third experimental repeat (red dots in a). Legend to right shows what color line in b-d correlates to what gap size. These colors also correlate to the gap size color in Figure S6.

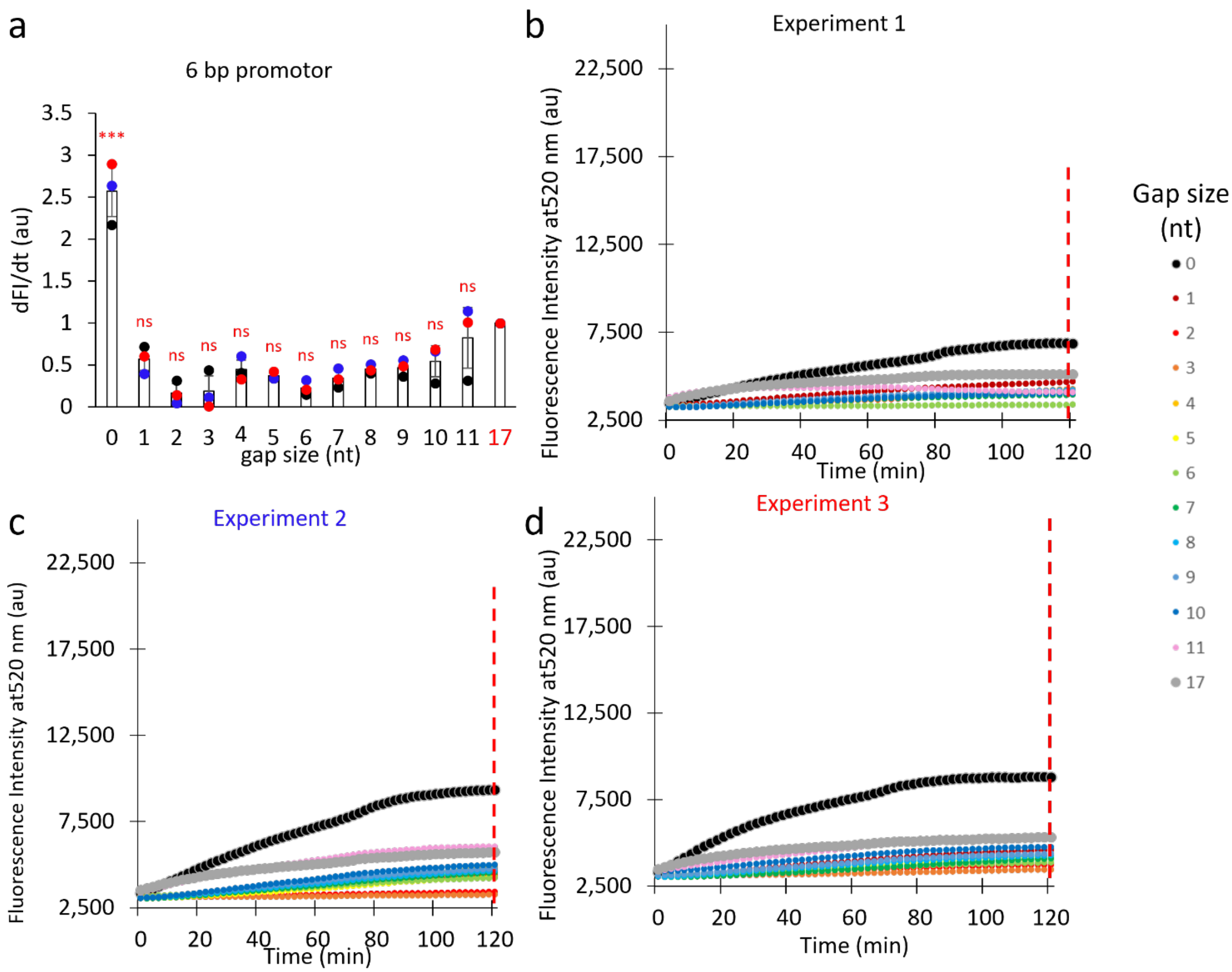

Figure S21: FAM molecular beacon fluorescence intensity transcription tracking for 6 bp promoter structure with gaps of various sizes. a) fluorescence increase during the linear phase, normalized to the 17 nt gap negative control (17), n=3 (black, blue, red dots), bar graphs are mean, error bars are s.d. Red significance marks show one tailed homoscedastic T-tests in relation to the negative control, highlighted red; ns: p>.05, ***: p<.001. Linear phase is denoted by red dashed lines in b) raw data for the first experimental repeat (black dots in a), c) raw data for the second experimental repeat (blue dots in a), and d) raw data for the third experimental repeat (red dots in a). Legend to right shows what color line in b-d correlates to what gap size. These colors also correlate to the gap size color in Figure S7.

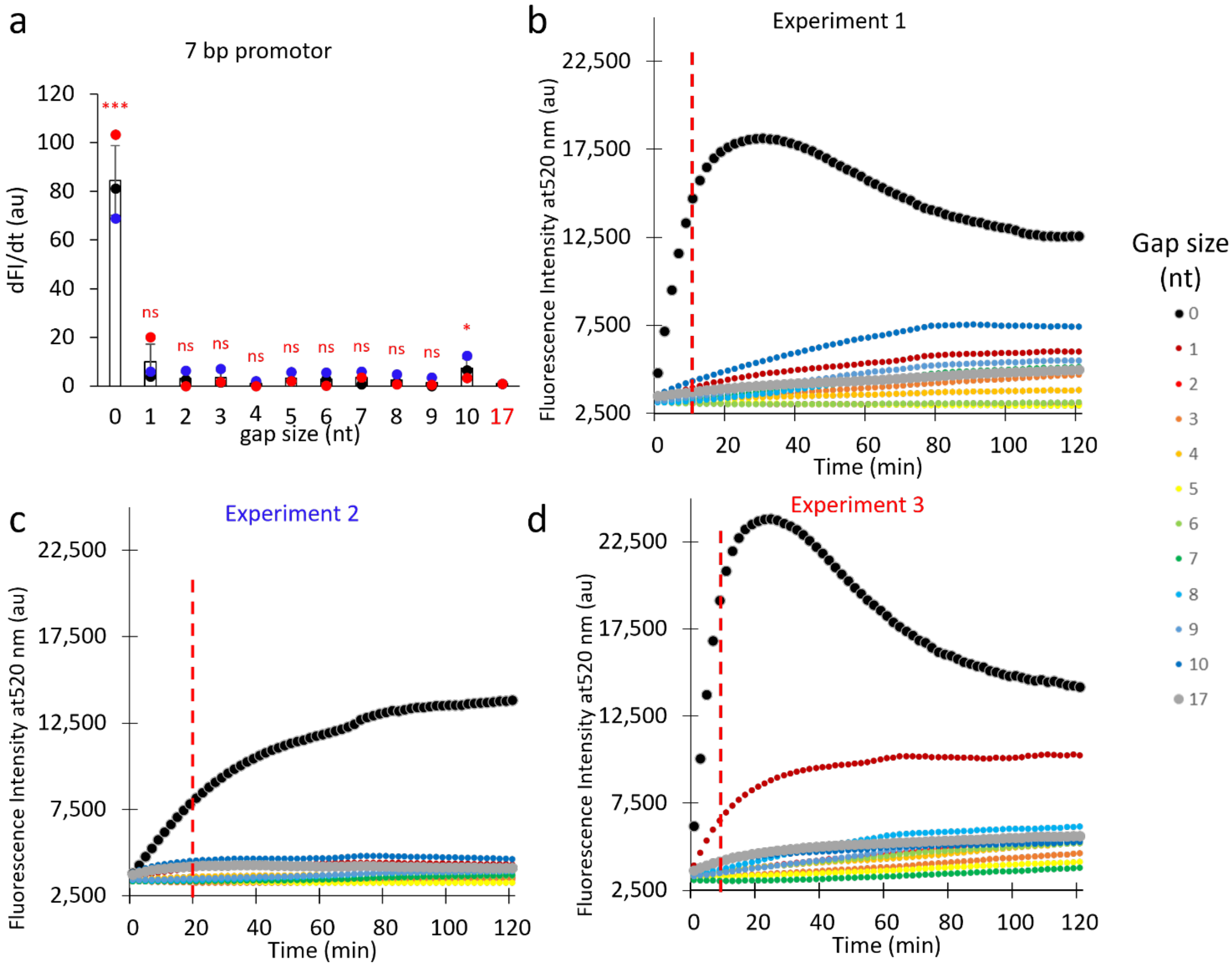

Figure S22: FAM molecular beacon fluorescence intensity transcription tracking for 7 bp promoter structure with gaps of various sizes. a) fluorescence increase during the linear phase, normalized to the 17 nt gap negative control (17), n=3 (black, blue, red dots), bar graphs are mean, error bars are s.d. Red significance marks show one tailed homoscedastic T-tests in relation to the negative control, highlighted red; ns: p>.05, *: p<.05, ***: p<.001. Linear phase is denoted by red dashed lines in b) raw data for the first experimental repeat (black dots in a), c) raw data for the second experimental repeat (blue dots in a), and d) raw data for the third experimental repeat (red dots in a). Legend to right shows what color line in b-d correlates to what gap size. These colors also correlate to the gap size color in Figure S8.

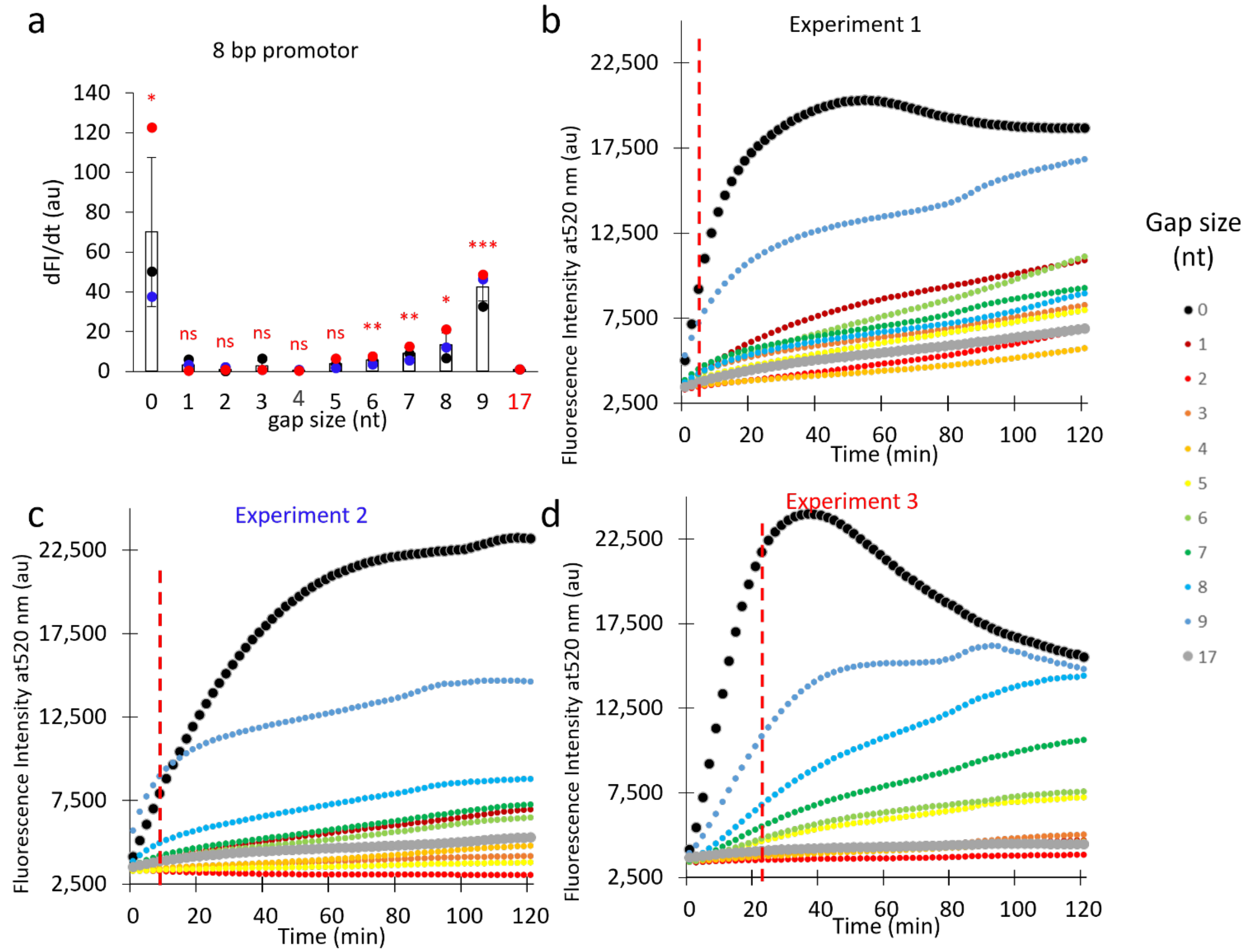

Figure S23: FAM molecular beacon fluorescence intensity transcription tracking for 8 bp promoter structure with gaps of various sizes. a) fluorescence increase during the linear phase, normalized to the 17 nt gap negative control (17), n=3 (black, blue, red dots), bar graphs are mean, error bars are s.d. Red significance marks show one tailed homoscedastic T-tests in relation to the negative control, highlighted red; ns: p>.05, *: p<.05, **: p<.01, ***: p<.001. Linear phase is denoted by red dashed lines in b) raw data for the first experimental repeat (black dots in a), c) raw data for the second experimental repeat (blue dots in a), and d) raw data for the third experimental repeat (red dots in a). Legend to right shows what color line in b-d correlates to what gap size. These colors also correlate to the gap size color in Figure S9.

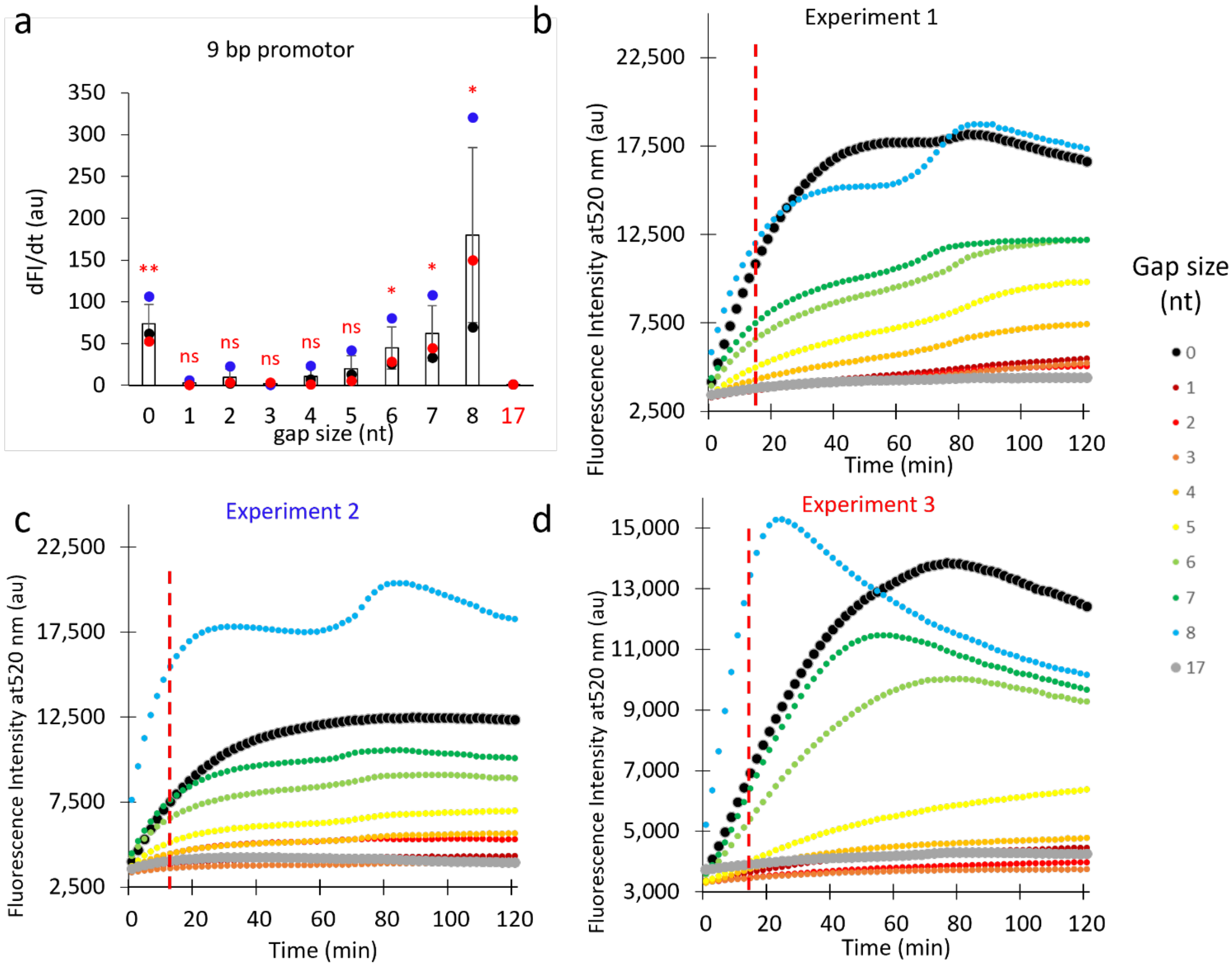

Figure S24: FAM molecular beacon fluorescence intensity transcription tracking for 9 bp promoter structure with gaps of various sizes. a) fluorescence increase during the linear phase, normalized to the 17 nt gap negative control (17), n=3 (black, blue, red dots), bar graphs are mean, error bars are s.d. Red significance marks show one tailed homoscedastic T-tests in relation to the negative control, highlighted red; ns: p>.05, *: p<.05, **: p<.01. Linear phase is denoted by red dashed lines in b) raw data for the first experimental repeat (black dots in a), c) raw data for the second experimental repeat (blue dots in a), and d) raw data for the third experimental repeat (red dots in a). Legend to right shows what color line in b-d correlates to what gap size. These colors also correlate to the gap size color in Figure S10.

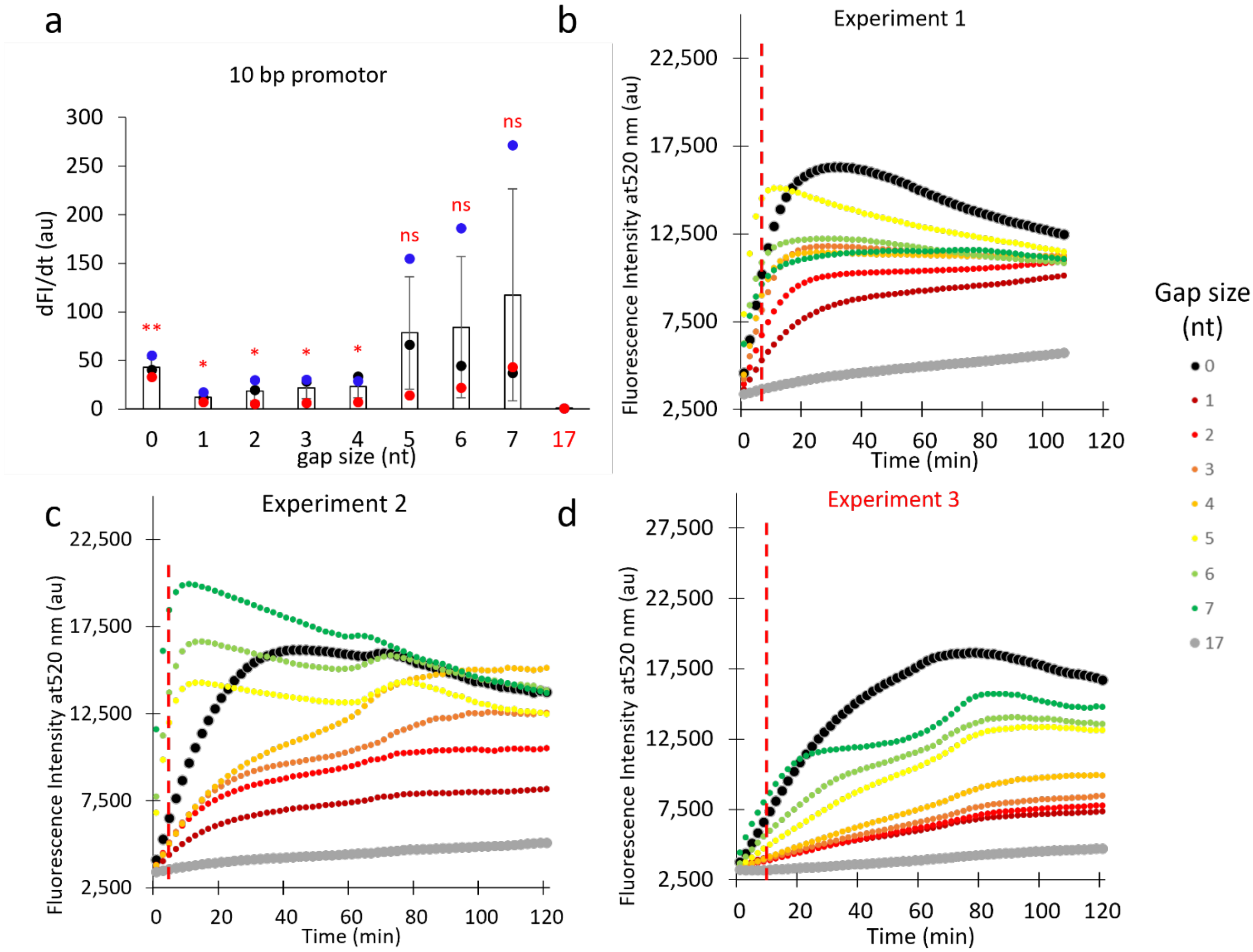

Figure S25: FAM molecular beacon fluorescence intensity transcription tracking for 10 bp promoter structure with gaps of various sizes. a) fluorescence increase during the linear phase, normalized to the 17 nt gap negative control (17), n=3 (black, blue, red dots), bar graphs are mean, error bars are s.d. Red significance marks show one tailed homoscedastic T-tests in relation to the negative control, highlighted red; ns: p>.05, *: p<.05, **: p<.01. Linear phase is denoted by red dashed lines in b) raw data for the first experimental repeat (black dots in a), c) raw data for the second experimental repeat (blue dots in a), and d) raw data for the third experimental repeat (red dots in a). Legend to right shows what color line in b-d correlates to what gap size. These colors also correlate to the gap size color in Figure S11.

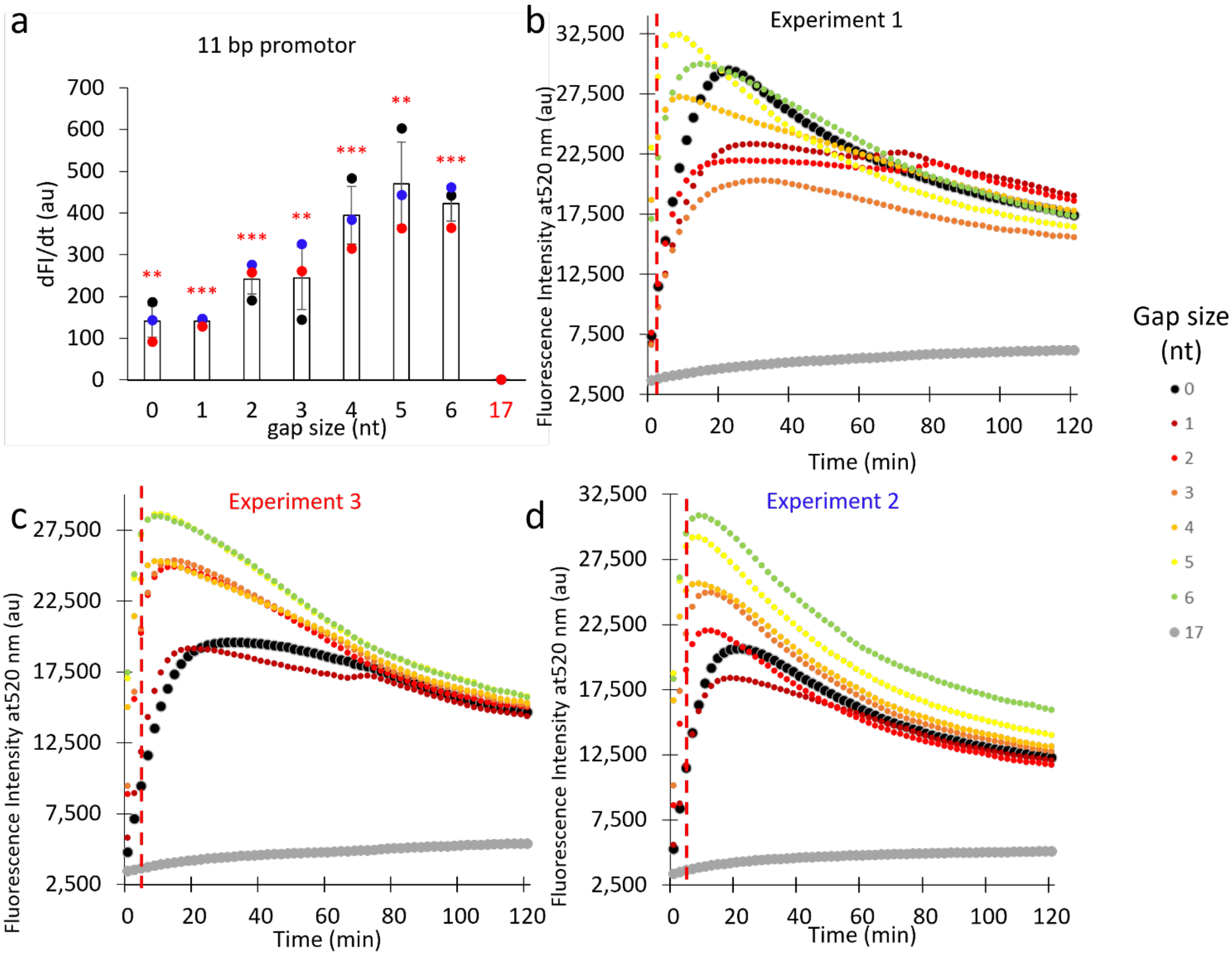

Figure S26: FAM molecular beacon fluorescence intensity transcription tracking for 11 bp promoter structure with gaps of various sizes. a) fluorescence increase during the linear phase, normalized to the 17 nt gap negative control (17), n=3 (black, blue, red dots), bar graphs are mean, error bars are s.d. Red significance marks show one tailed homoscedastic T-tests in relation to the negative control, highlighted red; **: p<.01, ***: p<.001. Linear phase is denoted by red dashed lines in b) raw data for the first experimental repeat (black dots in a), c) raw data for the second experimental repeat (blue dots in a), and d) raw data for the third experimental repeat (red dots in a). Legend to right shows what color line in b-d correlates to what gap size. These colors also correlate to the gap size color in Figure S12.

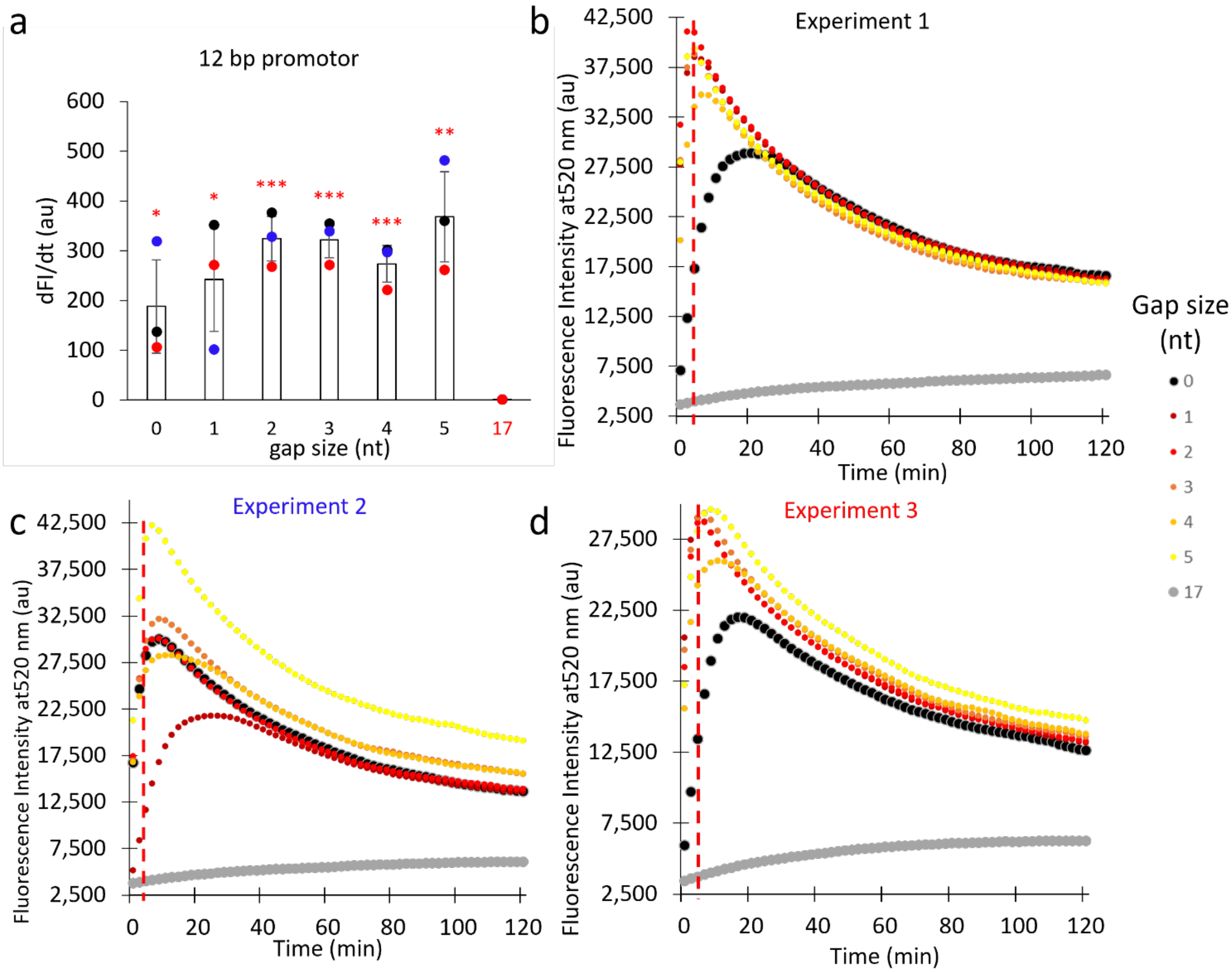

Figure S27: FAM molecular beacon fluorescence intensity transcription tracking for 12 bp promoter structure with gaps of various sizes. a) fluorescence increase during the linear phase, normalized to the 17 nt gap negative control (17), n=3 (black, blue, red dots), bar graphs are mean, error bars are s.d. Red significance marks show one tailed homoscedastic T-tests in relation to the negative control, highlighted red; *: p<.05, **: p<.01, ***: p<.001. Linear phase is denoted by red dashed lines in b) raw data for the first experimental repeat (black dots in a), c) raw data for the second experimental repeat (blue dots in a), and d) raw data for the third experimental repeat (red dots in a). Legend to right shows what color line in b-d correlates to what gap size. These colors also correlate to the gap size color in Figure S13.

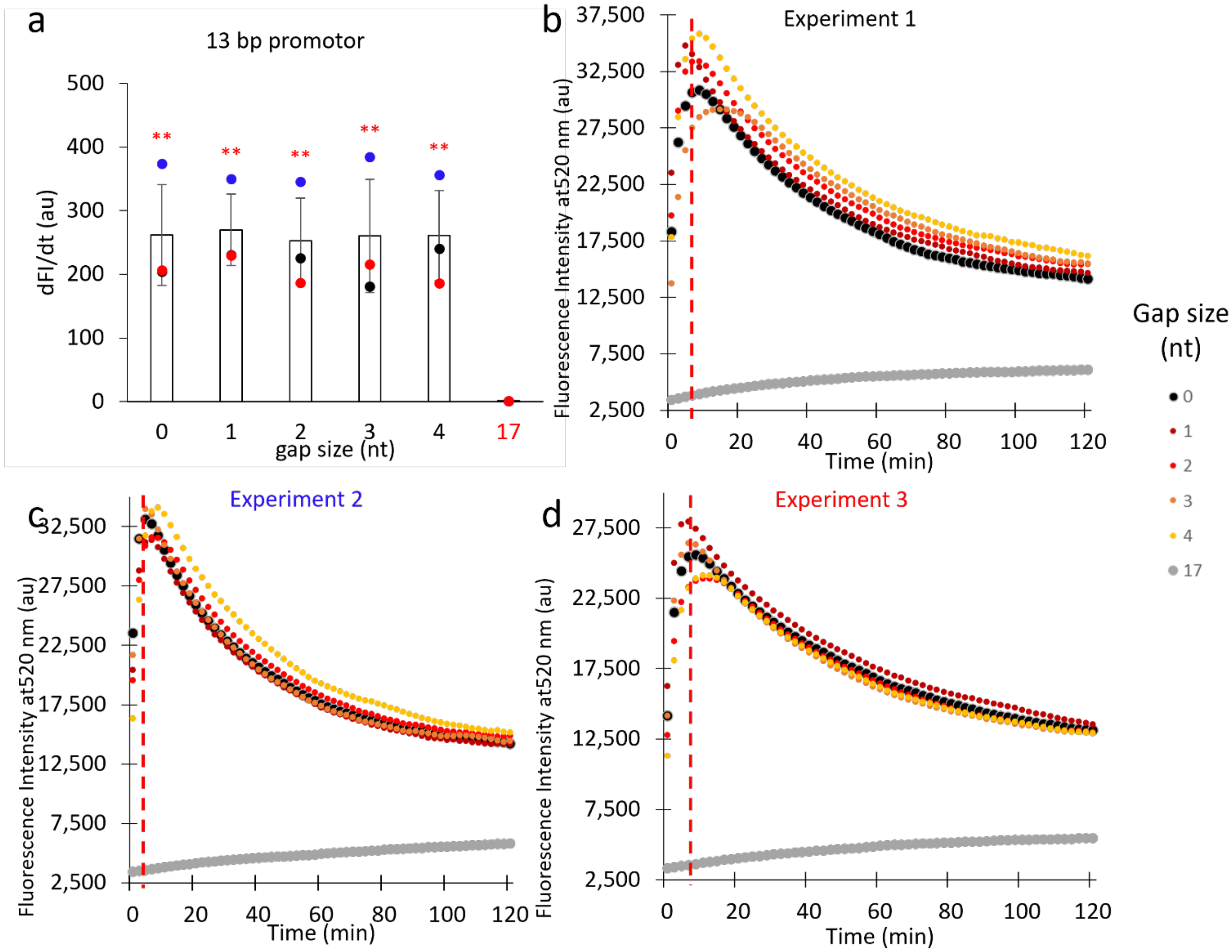

Figure S28: FAM molecular beacon fluorescence intensity transcription tracking for 13 bp promoter structure with gaps of various sizes. a) fluorescence increase during the linear phase, normalized to the 17 nt gap negative control (17), n=3 (black, blue, red dots), bar graphs are mean, error bars are s.d. Red significance marks show one tailed homoscedastic T-tests in relation to the negative control, highlighted red; **: p<.01. Linear phase is denoted by red dashed lines in b) raw data for the first experimental repeat (black dots in a), c) raw data for the second experimental repeat (blue dots in a), and d) raw data for the third experimental repeat (red dots in a). Legend to right shows what color line in b-d correlates to what gap size. These colors also correlate to the gap size color in Figure S14.

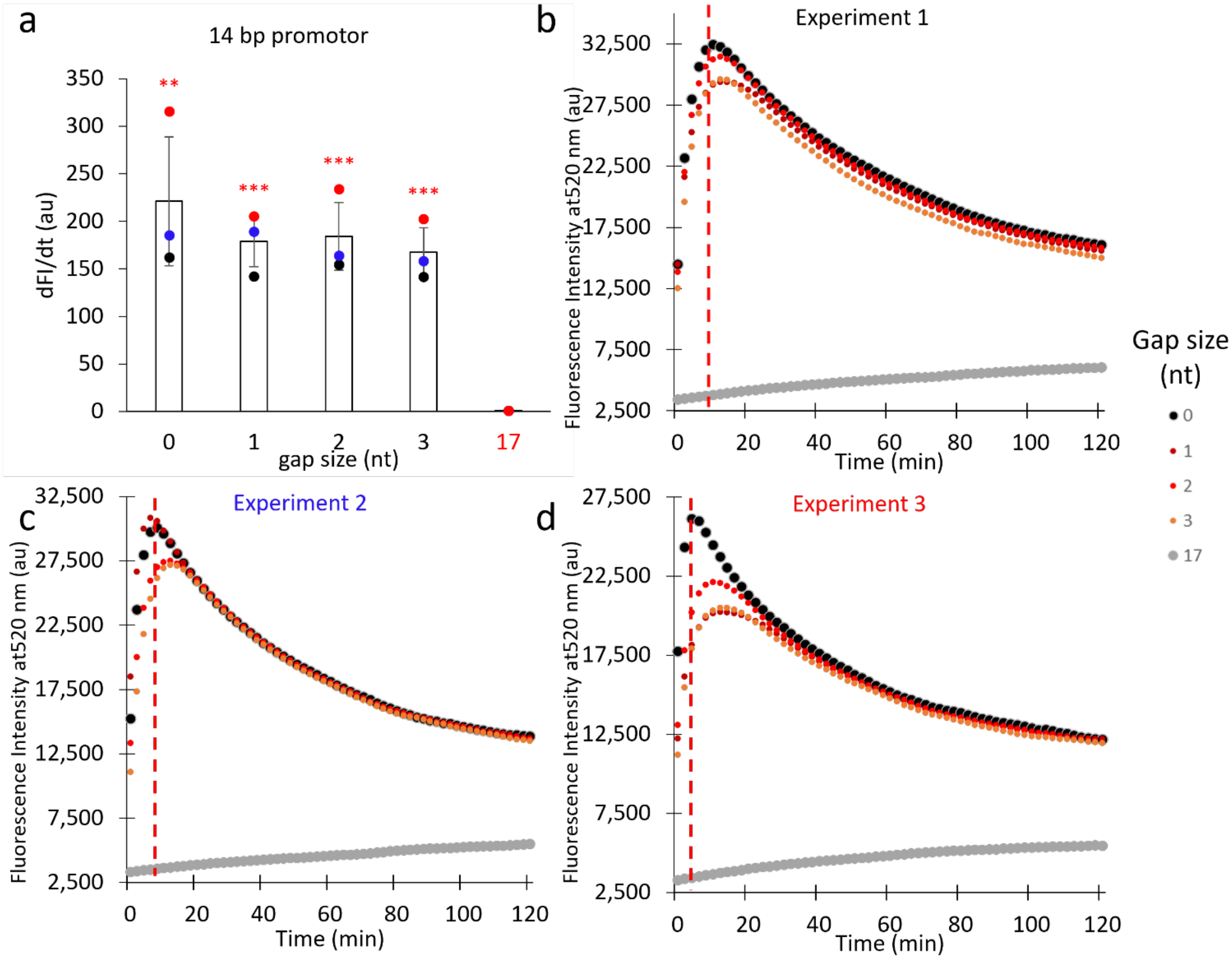

Figure S29: FAM molecular beacon fluorescence intensity transcription tracking for 14 bp promoter structure with gaps of various sizes. a) fluorescence increase during the linear phase, normalized to the 17 nt gap negative control (17), n=3 (black, blue, red dots), bar graphs are mean, error bars are s.d. Red significance marks show one tailed homoscedastic T-tests in relation to the negative control, highlighted red; **: p<.01, ***: p<.001. Linear phase is denoted by red dashed lines in b) raw data for the first experimental repeat (black dots in a), c) raw data for the second experimental repeat (blue dots in a), and d) raw data for the third experimental repeat (red dots in a). Legend to right shows what color line in b-d correlates to what gap size. These colors also correlate to the gap size color in Figure S15.

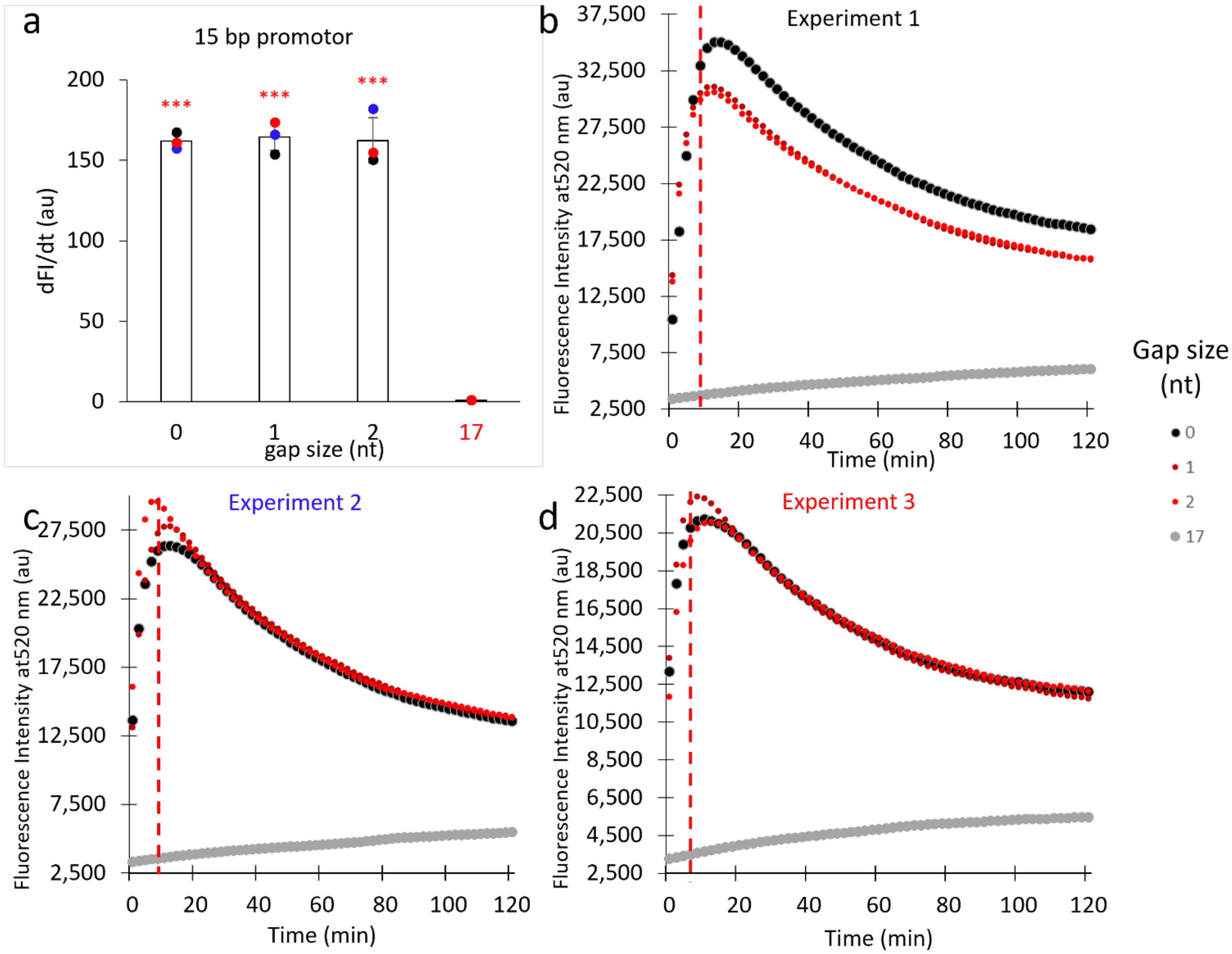

Figure S30: FAM molecular beacon fluorescence intensity transcription tracking for 15 bp promoter structure with gaps of various sizes. a) fluorescence increase during the linear phase, normalized to the 17 nt gap negative control (17), n=3 (black, blue, red dots), bar graphs are mean, error bars are s.d. Red significance marks show one tailed homoscedastic T-tests in relation to the negative control, highlighted red; ***: p<.001. Linear phase is denoted by red dashed lines in b) raw data for the first experimental repeat (black dots in a), c) raw data for the second experimental repeat (blue dots in a), and d) raw data for the third experimental repeat (red dots in a). Legend to right shows what color line in b-d correlates to what gap size. These colors also correlate to the gap size color in Figure S16.

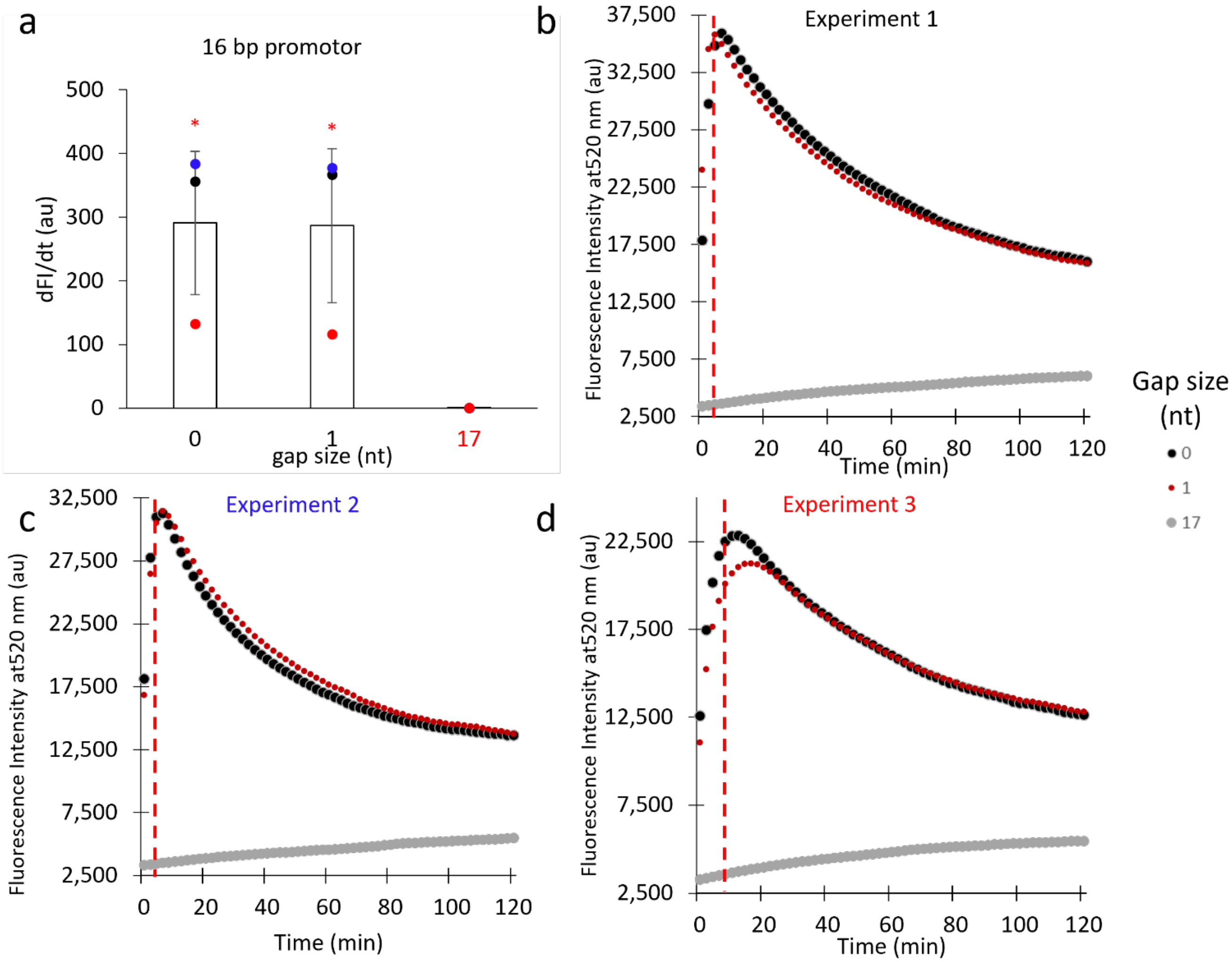

Figure S31: FAM molecular beacon fluorescence intensity transcription tracking for 16 bp promoter structure with gaps of various sizes. a) fluorescence increase during the linear phase, normalized to the 17 nt gap negative control (17), n=3 (black, blue, red dots), bar graphs are mean, error bars are s.d. Red significance marks show one tailed homoscedastic T-tests in relation to the negative control, highlighted red; *: p<.05. Linear phase is denoted by red dashed lines in b) raw data for the first experimental repeat (black dots in a), c) raw data for the second experimental repeat (blue dots in a), and d) raw data for the third experimental repeat (red dots in a). Legend to right shows what color line in b-d correlates to what gap size. These colors also correlate to the gap size color in Figure S17.

Supplementary Text ST2: concerning nick and 1nt gap moved throughout promoter sequence

Since the data shown in Figure 1c of the main text was collected during different experiments, ie each # bp structure was its own experiment, it would be improper to use such data to ascertain how a gap of a specific size affects transcription as it is shifted along the promote sequence. As such, we repeated the transcription assays for a 0 nt gap (nick) and a 1 nt gap at each location in the non-template strand promoter sequence, compared against a fully double-stranded positive control, shown in Figure S32, as well as a negative control with a 17 nt gap in the promoter region. These experiments will be called “comprehensive”.

The results shown in Figures S33 and S34 are comparable to those collected and consolidated from the separate experiments (henceforth called “consolidated”), which supports the reproducibility of our findings and provides evidence that one actually could use the data in Figure 1c to say how a certain gap size affects transcription as it shifts along the promoter (though we would still advocate for direct experimentation to say for sure). Notably, the position of the 1 nt gap has more of an influence on transcription rate than the position of the nick, except in cases where nicks occur just 5’ of positions -12 and -11. In those cases, transcription drops to levels comparable to the negative control. Given that these constructs were composed of synthetic DNA, a nick represents a break in the phosphate backbone and lacks the 5’ phosphate group.

To test whether the missing phosphate was responsible for the reduced transcription, we repeated the -12 and -11 nick experiments using 5’ phosphorylated versions of the relevant 3’ coding strands. As shown in Figure S35, phosphorylation had no effect until the nick was enzymatically ligated, after which transcription activity returned to the level of the positive control. Interestingly, both the consolidated and comprehensive experiments show that introducing either a nick or 1 nt gap downstream of position -6 results in a transcription rate higher than that of the fully intact promoter. This finding suggests that structural defects in the T7 promoter initiation region may improve the transcription rate[4, 7-8]. Furthermore, these experiments show that the 1 nt gaps between positions -15 to -7 drastically reduce transcription, while only nicks 5’ of positions -12 and -11 have a similar effect.

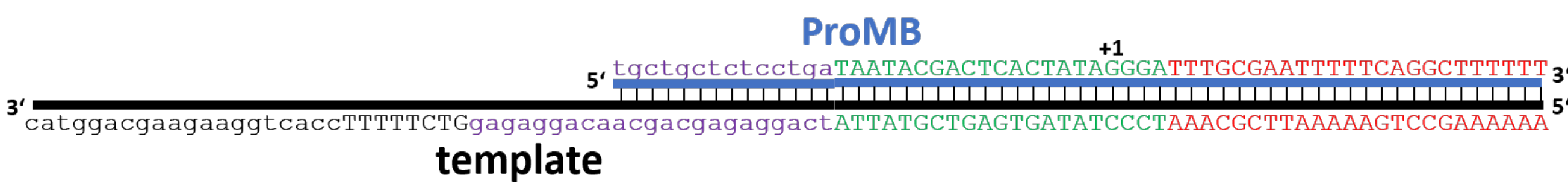

Figure S32: Schematic for positive control used in comprehensive, verification, and control experiments using MB. The coding strand (blue) is completely complementary to the template strand (black) from positions -31 to +28, which ensures the entire promoter (green) and MB target (red) regions are double-stranded. The purple region is the handle region, and the black bases on the template strand upstream of the handle are unused. Transcription start site (+1, G) is marked in the coding strand.

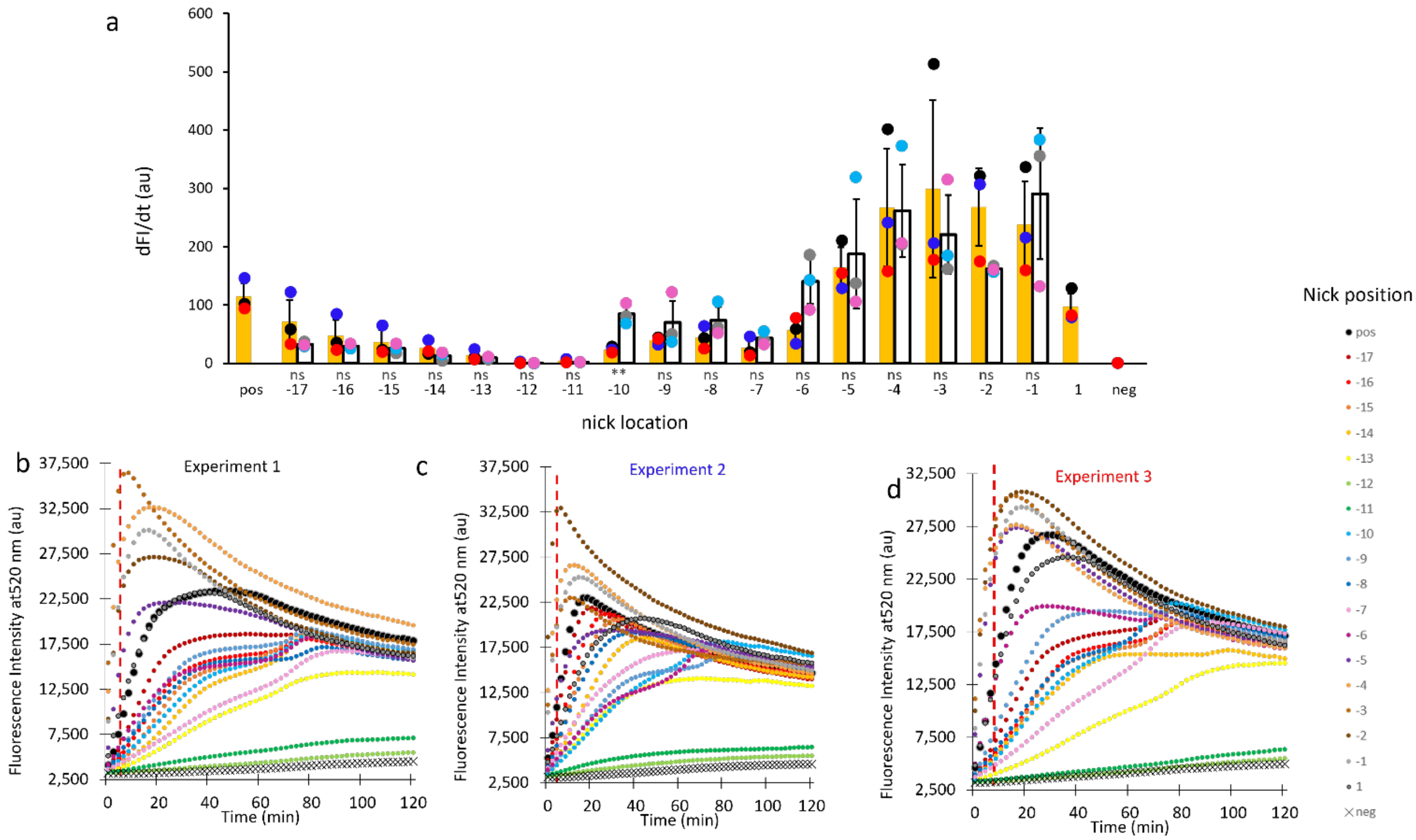

Figure S33: T7 transcription of substrates with promoters interrupted by a nick in the T7 promoter sequence. a) Transcription rate during linear phase collected during the same experiment (gold bars) vs data consolidated from separate experiments shown in Figure 1 using the # bp promoters (white bars). Bar graphs show the mean slope of FAM fluorescence increase during the linear phase, normalized to the 17 nt gap negative control (neg) over n=3 repeats (black, red, blue dots for comprehensive data or grey, cyan, pink dots for consolidated data), error bars are s.d. Black significance marks below the x-axis show two tailed homoscedastic T-tests comparing consolidated data to comprehensive data; ns: not significant, **: p<.01. Raw data for FAM molecular beacon fluorescence intensity transcription tracking for comprehensive experiment moving a nick (0 nt gap) throughout the T7 promoter sequence for b) the first experimental repeat (black dots in a), c) the second experimental repeat (blue dots in a), and d) for the third experimental repeat (red dots in a). Legend to right shows what color line correlates to where the nick is in the promoter sequence. Note: the nick is just 5' of the indicated position. Red dashed lines show where the linear phase ends for rate calculation. The positive control (pos, black dots) is fully double-stranded and the negative control (neg, black "X") has a 17 nt gap in the promoter sequence.

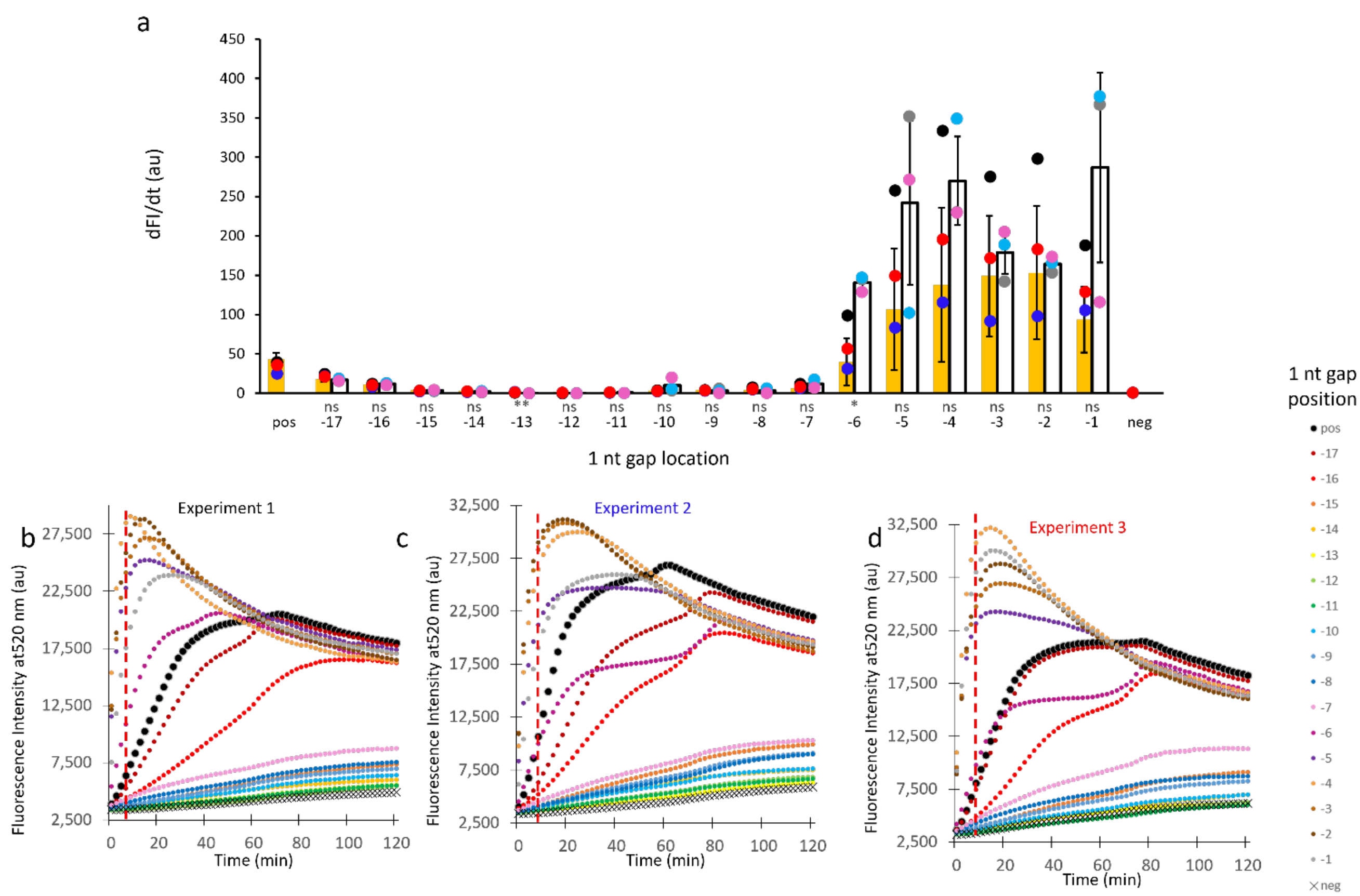

Figure S34: T7 transcription of substrates with promoters interrupted by a 1 nt gap in the T7 promoter sequence. a) Transcription rate during linear phase collected during the same experiment (gold bars) vs data consolidated from separate experiments shown in Figure 1 using the # bp promoters (white bars). Bar graphs show the mean slope of FAM fluorescence increase during the linear phase, normalized to the 17 nt gap negative control (neg) over n=3 repeats (black, red, blue dots for comprehensive data or grey, cyan, pink dots for consolidated data), error bars are s.d. Black significance marks below the x-axis show two tailed homoscedastic T-tests comparing consolidated data to comprehensive data; ns: not significant, *: p<.05, **: p<.01. Raw data for FAM molecular beacon fluorescence intensity transcription tracking for comprehensive experiment moving a nick (0 nt gap) throughout the T7 promoter sequence for b) the first experimental repeat (black dots in a), c) the second experimental repeat (blue dots in a), and d) for the third experimental repeat (red dots in a). Legend to right shows what color line correlates to where the nick is in the promoter sequence. Red dashed lines show where the linear phase ends for rate calculation. The positive control (pos, black dots) is fully double-stranded and the negative control (neg, black "X") has a 17 nt gap in the promoter sequence.

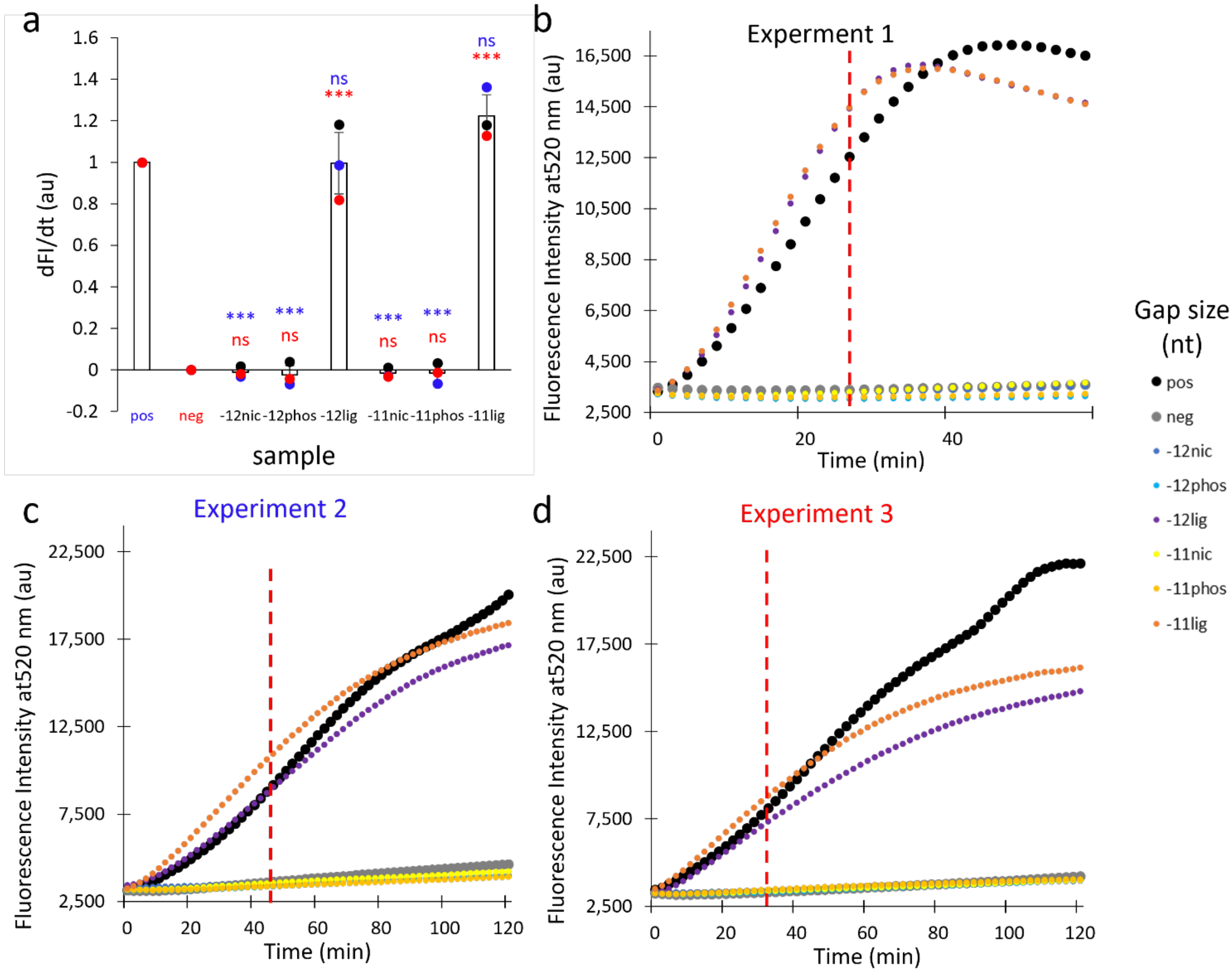

Figure S35: FAM molecular beacon fluorescence intensity transcription tracking for effect of phosphorylation (phos) and ligation (lig) of a nick (nic) 5' of positions -12 (shades of blue) or -11 (shades of yellow). a) fluorescence increase during the linear phase, range normalized to a fully double-stranded positive control (pos, black dots) and a negative control (neg, grey dots) with a 17 nt gap in the promoter sequence, n=3 (black, blue, red dots), bar graphs are mean, error bars are s.d. Red significance marks show one tailed homoscedastic T-tests in relation to the negative control, highlighted red. Blue significance marks show one tailed homoscedastic T-tests in relation to the positive control, highlighted blue. ns: p>.05, ***: p<.001Linear phase is denoted by red dashed lines in b) raw data for the first experimental repeat (black dots in a), c) raw data for the second experimental repeat (blue dots in a), and d) raw data for the third experimental repeat (red dots in a). Legend to right shows what color line in b-d correlates to what sample.

Supplementary Text ST3: on early plateauing mentioned in main

The verification experiments shown in Figure 2a of the main text only illustrate the transcription rate during the linear phase of pepper aptamer transcription; however, as mentioned in the main text, the raw data exhibit an early plateau effect that needs to be investigated. Specifically, the pepper fluorescence signal for the 9 bp promoter with an 8 nt gap, decreases early on (cyan dots in Figure S37) compared to the same structure transcribing an MB target (cyan dots in Figure S24), which levels off either with, or well above the level of the nicked reference sample (thick black dots in Fig S24).

One possible explanation for this different behavior emerged from using polyacrylamide gel electrophoresis (PAGE; Figure S38), in which the band of the RNA transcription product for sample with the 8 nt gap was less intense than the corresponding band in the lanes of the positive control or the sample with the 0 nt gap. This reduced yield of full-length transcription product likely contributes to why the signal intensity of the sample with the 8 nt gap levels off much earlier that the nicked samples or positive controls, and is consistent with the T7RNAP not releasing the promoter to enter into the elongation phase. Transcription is still possible in this state, and T7RNAP can produce transcripts even up to 26 nt and still remain on the promoter.[9] The pepper aptamer is still being transcribed, albeit at a much reduced efficiency and this is consistent with the results of an early plateau in 8 nt gap sample in Figure S37 and a faint product band in lane 8TD of Figure S38.

To quantify the amount of final transcription product generated, we repeated the transcription assays using the pepper aptamer and included a standard curve generated based on the published pepper aptamer sequence[9] (which is known to function; sequence in Table S1 “pepper reference”). The standard curve was generated by adding 10 µL of the pepper aptamer at 50, 100, 200, 400, and 800 nM to 20 µL of transcription master mix (so final aptamer concentrations were: 17, 33, 67, 133, and 267 nM, respectively) and measuring it simultaneously with the repeated tests using substrates with gaps at 100 nM substrate concentration, 0.5 mM NTP, and ca. 50 U T7RNAP. The results in Figure S39a show that the 9 bp promoter substrate with an 8 nt gap typically transcribes about 2.5 times the substrate concentration when the substrate is present at 100 nM before reaching a dynamic equilibrium between fluorescent signal decay rate and the transcription rate.

To further investigate the hypothesis that substrates with larger gaps impair promoter release, transcription of the 9 bp promoter with an 8 nt gap was repeated under various reaction conditions, namely at substrate concentrations of either 10, 50, and 100 nM, with NTPs at concentrations of either 0.5 or 2 mM and T7RNAP at either 50 or 150 U per sample. The results (Figure S39b) show that the 9 bp promoter structure with an 8 nt gap reaches a plateau early on, which is consistent with the conditions expected for transcription: lower concentrations result in low transcription efficiency and overall weaker signal. This is consistent with a lower rate of promoter release, as substrates that spend more time attached to the promoter are less likely proceed to elongation. Furthermore, at lower concentrations, those polymerases that do move into elongation are less likely to find a new polymerase at lower concentrations. An increase in T7RNAP concentration provides more opportunities for entry into elongation, and the overall signal increases. However, an increase in NTP concentration appears to slightly reduce the overall signal.

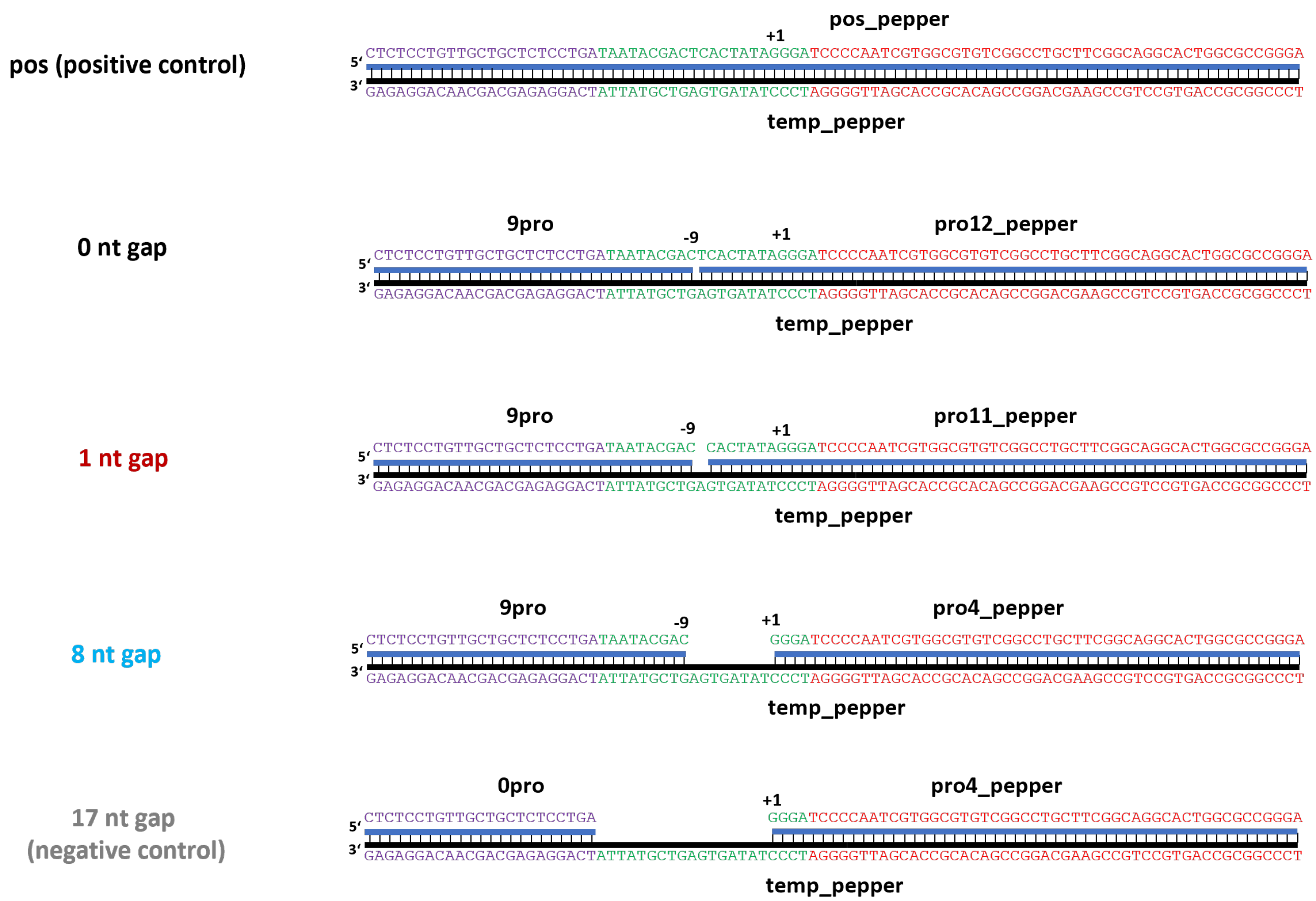

Figure S36: Schematics for transcription substrates encoding a pepper fluorogenic aptamer. For the positive control, the coding strand (top) is completely complementary to the template strand (bottom), which ensures the entire promoter (green) and pepper (red) regions are entirely double-stranded. The 0, 1, and 8 nt gap samples have either a 0, 1, or 8 nt single-stranded gap, respectively, after position -9. The negative control has a 17 nt single-stranded gap across the entire promoter sequence. The purple region is the handle region. Transcription start site (+1, G) is marked in the coding strand. The colors of the substrate names correlate to the curve color describing that substrates transcription in Figure S37.

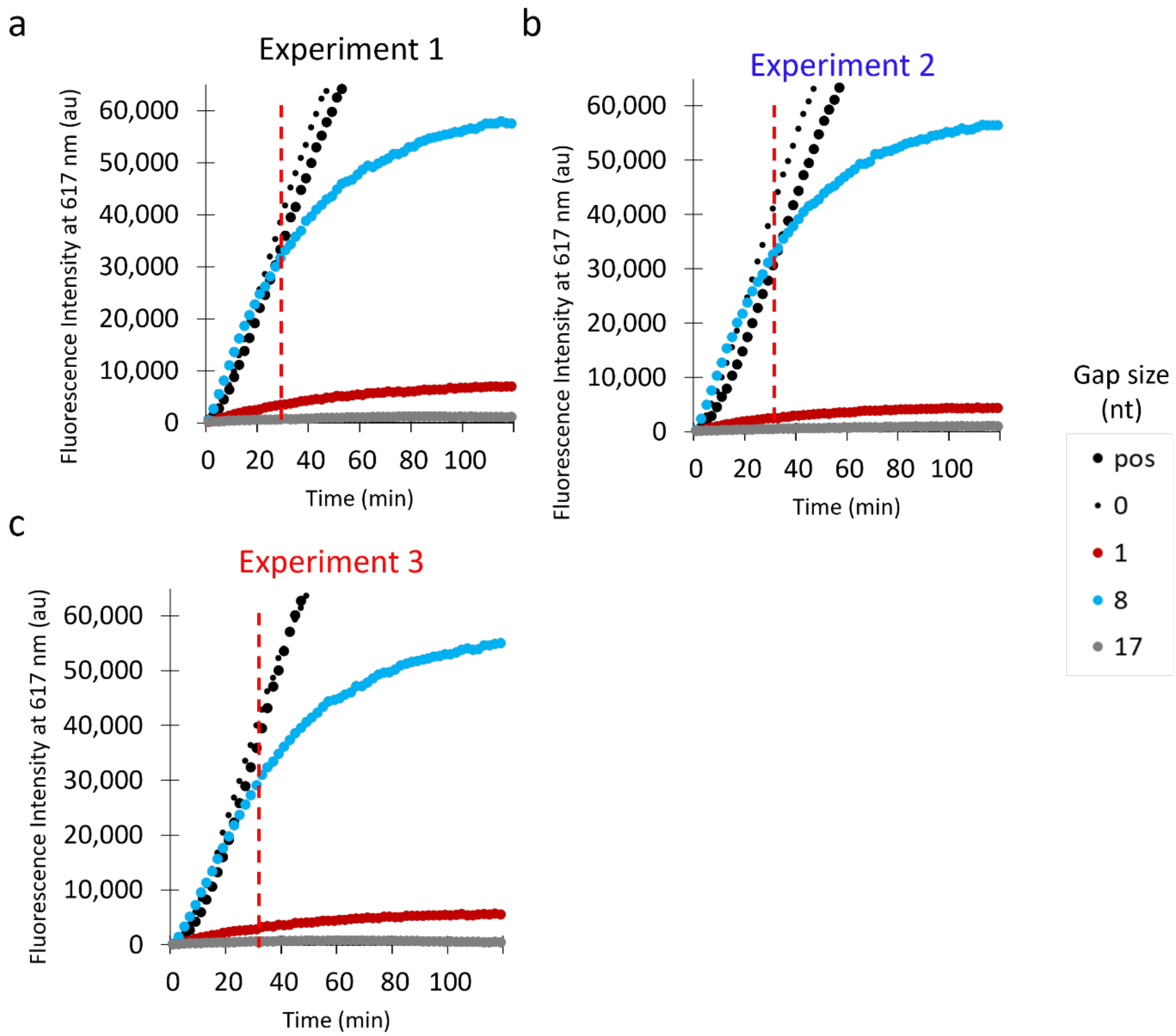

Figure S37: Raw data for pepper transcription fluorescence intensity transcription tracking for a) the first experimental repeat (black dots in Figure 2a), b) the second experimental repeat (blue dots in Figure 2a), and c) for the third experimental repeat (red dots in Figure 2a). Legend to right shows what color line correlates to which substrate shown in Figure S36 is being transcribed. Red dashed lines show where the linear phase ends for rate calculation. Positive control (pos) is fully double-stranded and the negative control (neg) has a 17 nt gap in the promoter sequence

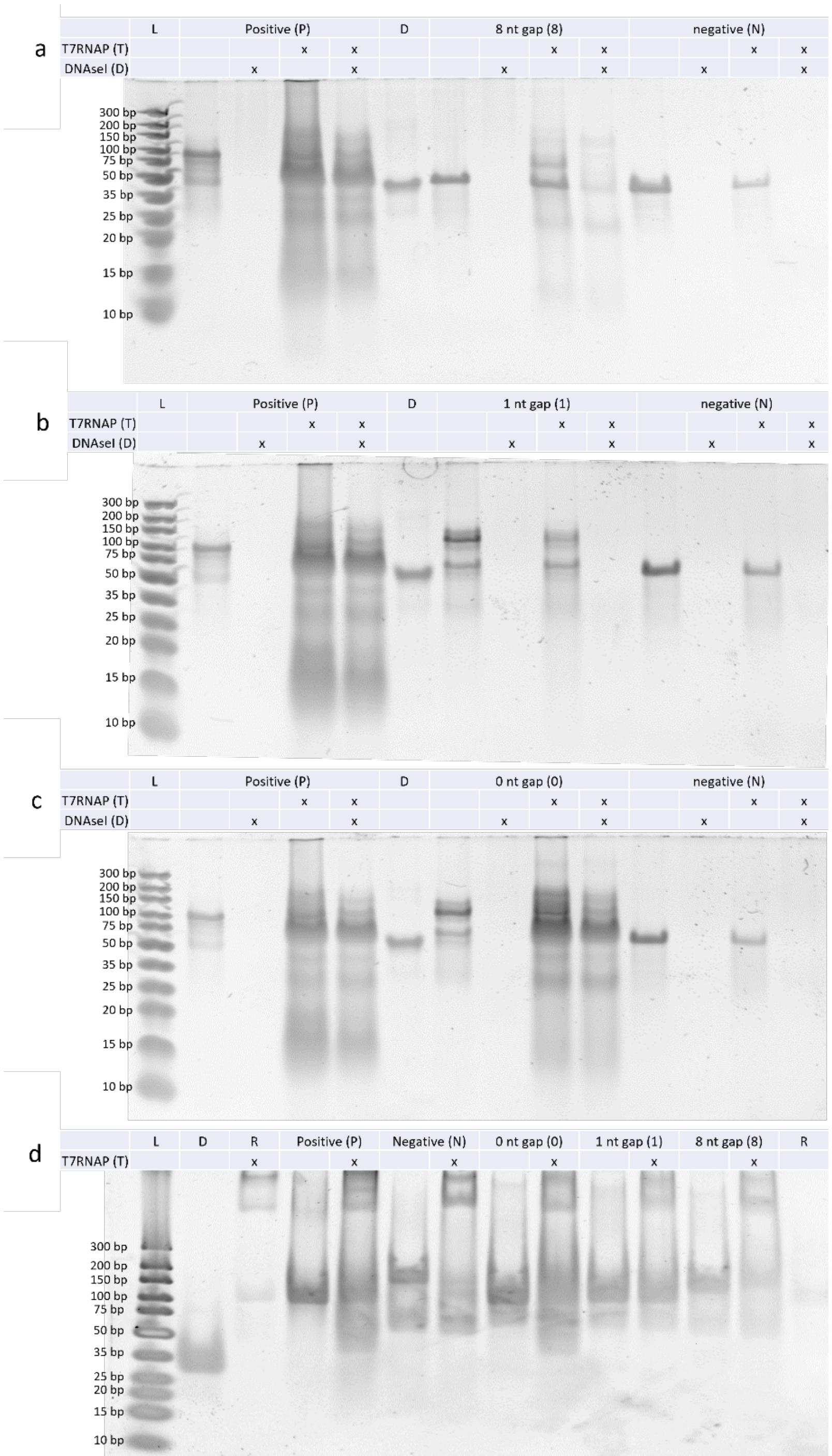

Figure S38: Original gels showing transcription products for 9 bp promoter structures encoding pepper aptamer with a) 8 nt gap (8), b) 1 nt gap (1) and c) 0 nt gap (0) in the promoter region, 15% Urea PAGE, 100 V, 90 min, SYBR gold stained, 4% PAGE stacking layer. d) SYBR gold stain of gel presented in Figure 2b, 8% PAGE 100 V, 30 min. All gels contain the fully double-stranded promoter positive control (P) and a negative control (N) with a 17 nt gap in the promoter region. Lanes are marked as to whether samples were transcribed with T7RNAP (T), DNAseI digested (D), or both with an “x” in the table above each gel. The ladder (L) is an ultra low range ladder from Thermo Fischer. D is a DNA marker (pro4_pepper, Table S1) and R is an RNA marker (Pepper marker, Table S1) of the length expected of the transcription product.

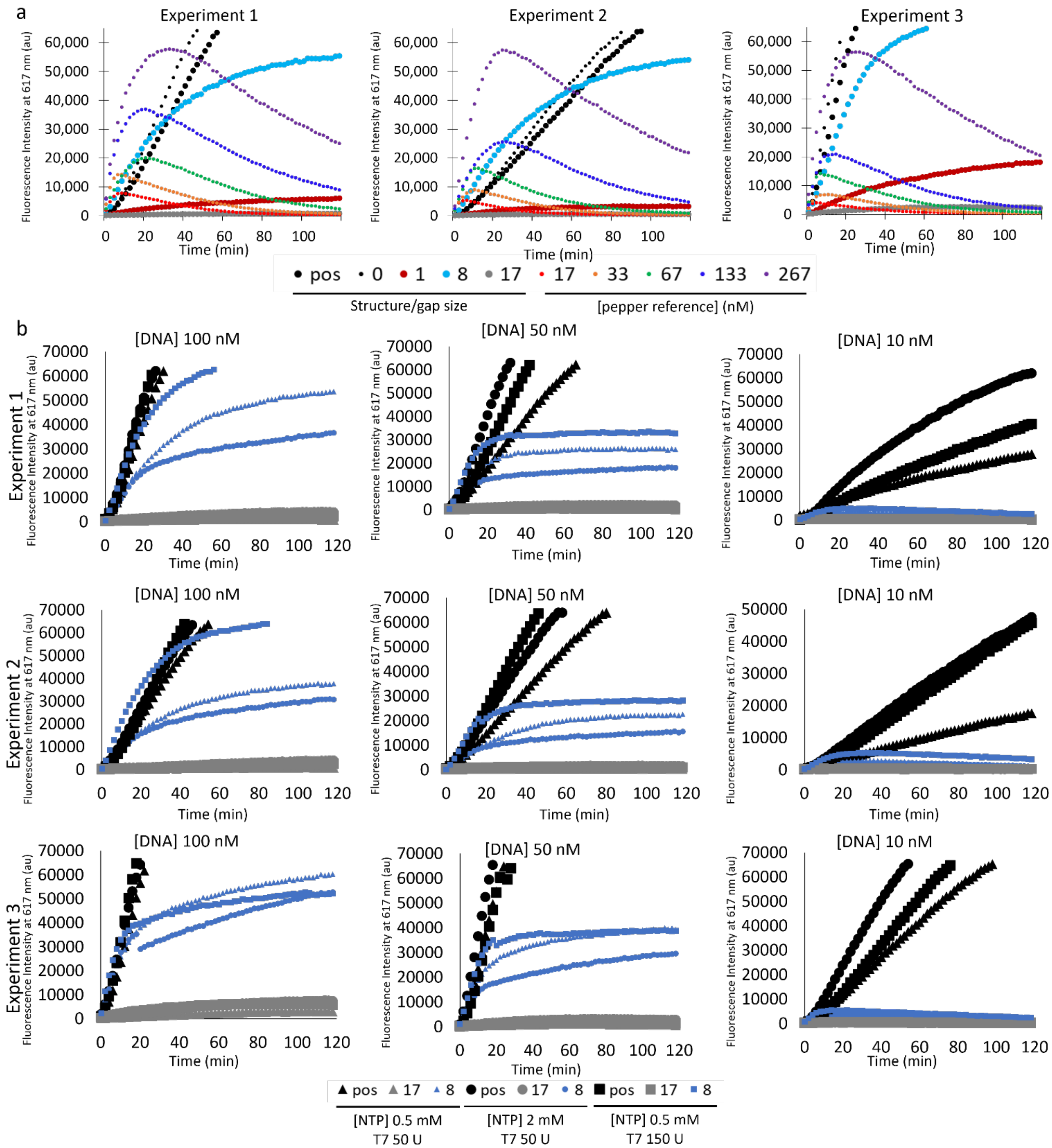

Figure S39: Raw fluorescence-intensity data (in triplicate), for Pepper transcription tracking. a) Quantification of full-length transcription products and b) assessment of changes in transcription-reagent concentrations (substrate DNA, Polymerase, and NTPs). The legends below each dataset indicate which substrate corresponds to each colored line. The positive control (pos, black) is fully double-stranded, whereas the negative control (17, grey) contains a 17-nt gap in the promoter sequence.

Supplementary Text ST4: on hybridization verification experiments:

Several additional experiments were conducted to confirm the reliability of the transcription results and rule out potential artifacts. First, to verify proper hybridization of the individual coding strands to the template strand, we performed melting curve analysis on duplexes formed between the template strand and either the 5' or 3' coding strand. As expected, the melting temperature increased with longer hybridized regions, consistent with proper and specific base pairing, shown in Figure S40. To further validate hybridization, gel electrophoresis was also performed, confirming the formation of the expected duplexes, shown in Figures S41-S45.

Next, we evaluated the possible impact of incomplete or unintended substrate assembly, since the template strand was added in excess during transcription reactions. This excess was intended to sequester unbound 3′ coding strand, which can otherwise activate MBs. However, this design allows for the formation of four substrate species: 1) the desired substrate with template strand hybridized to both 5' coding and 3' coding strands, 2) the template strand alone expected to be present due to being in excess, 3) an undesired substrate with template strand hybridized only to the 5' coding strand, and 4) an undesired substrate with template strand hybridized only to the 3' coding strand. Because the transcriptional behavior of these undesired species was unknown, we analyzed them independently to ensure they were not contributing to the observed effects. For the template + 5′ coding strand species, analyzed in Figures S46a and S47a–c, with schematics in Figure S48, transcription was negligible across most samples. The only exception was when the double-stranded region extended to at least position -5, a condition already known[4] to permit high transcription regardless of gap size. Thus, we conclude that this species does not significantly affect results for the 8, 9, or 10 bp promoter constructs. Similarly, for the template + 3′ coding strand species, analyzed in Figures S46b and S47d–f, with schematics in Figure S49 increased transcription was only observed when the double-stranded region started at position -15 or upstream, also in accordance with prior work.[4] Again, this is not relevant for the 8, 9, or 10 bp promoter structures, indicating that this species also does not influence our results in those cases. Together, these control experiments effectively rule out improper hybridization and unwanted partial substrate species as sources of error in the original transcription assays (Figures 1c, S15-S31).

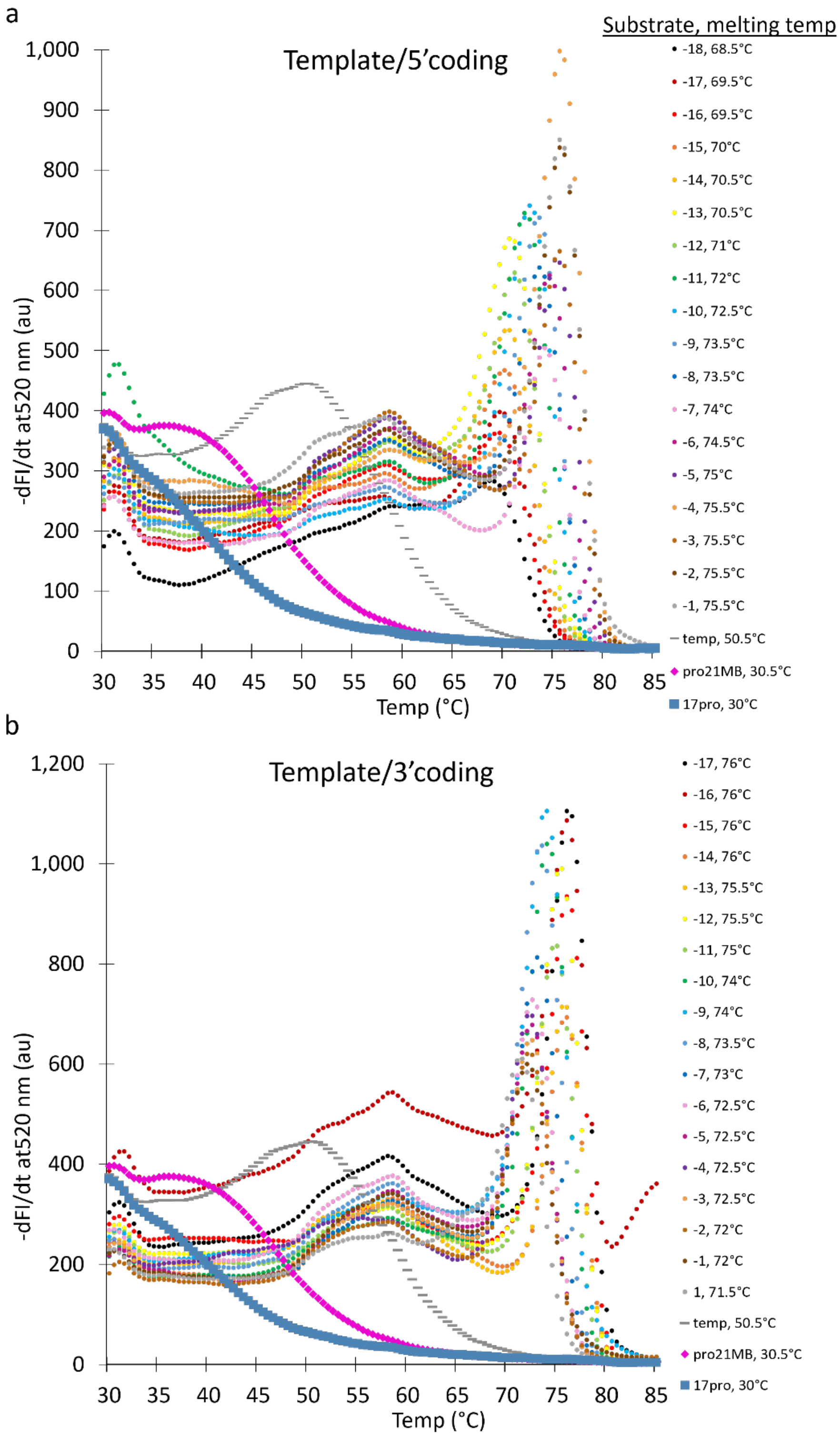

Figure S40: Negative derivative of melting curves for the template strand hybridized to a) the 5' coding strand or b) the 3' coding strand compared to the template strand alone, the longest possible 3' coding strand alone (pro21MB), and the longest 5' coding strand alone (17pro). Legend to right shows the substrate correlating its colored curve marking the position of the end of dsDNA (for 5' coding strand) or the beginning of dsDNA (for the 3' coding strand), as well as the melting temperature for that coding strand hybridized to the template strand. Schematics of structures are shown in Figures S48 and S49.

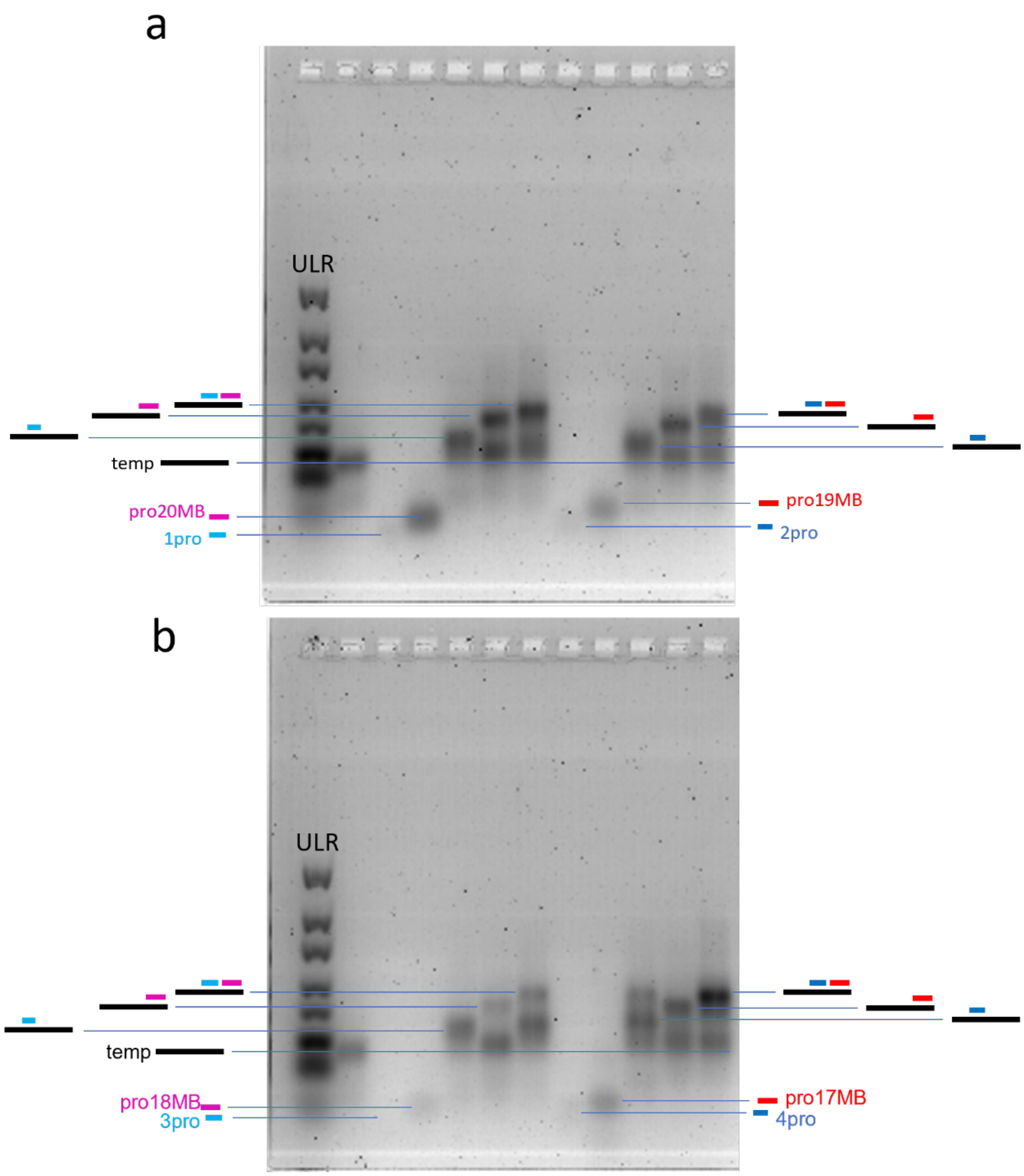

Figure S41: Gel electrophoresis of template strand (black) hybridization to coding strands a) 1pro (cyan) and/or pro20MB (magenta) as well as 2pro (blue) and/or pro19MB (red) and b) 3pro (cyan) and/or pro18MB (magenta) as well as 4pro (blue) and/or pro17MB (red). Lane labeled with ULR contains an Ultra Low Range ladder Gene Ruler from Thermo Fisher. Samples were prepared with template strand at 1.5x in 1x assembly buffer (described in Materials and Methods), heated to 95°C and annealed at 4°C for 30 min. 500 fmol of each sample was loaded on the gel, except for the coding strand alone lanes to which 1500 fmol was added (to try to increase visibility). 3% agarose, 1x TAE, 80 V, 60 mins, pre-stained with SYBR Gold. In every case, addition of a coding strand to the template results in an upward mobility shift, indicating successful hybridization.

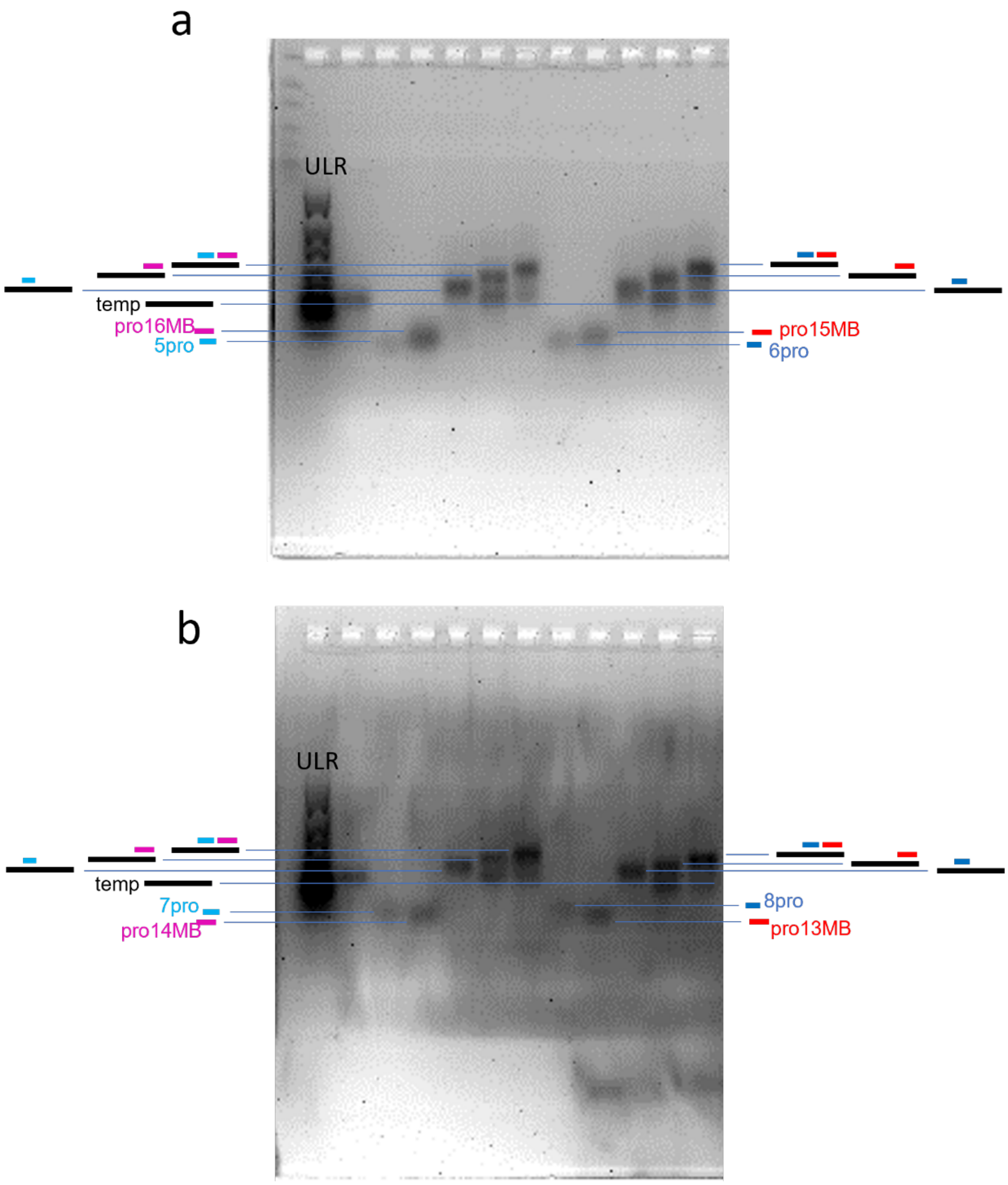

Figure S42: Gel electrophoresis of template strand (black) hybridization to coding strands a) 5pro (cyan) and/or pro16MB (magenta) as well as 6pro (blue) and/or pro15MB (red) and b) 7pro (cyan) and/or pro14MB (magenta) as well as 8pro (blue) and/or pro13MB (red). Lane labeled with ULR contains an Ultra Low Range ladder Gene Ruler from Thermo Fisher. Samples were prepared with template strand at 1.5x in 1x assembly buffer (described in Materials and Methods), heated to 95°C and annealed at 4°C for 30 min. 500 fmol of each sample was loaded on the gel, except for the coding strand alone lanes to which 1500 fmol was added (to try to increase visibility). 3% agarose, 1x TAE, 80 V, 40 mins, pre-stained with SYBR Gold. In every case, addition of a coding strand to the template results in an upward mobility shift, indicating successful hybridization.

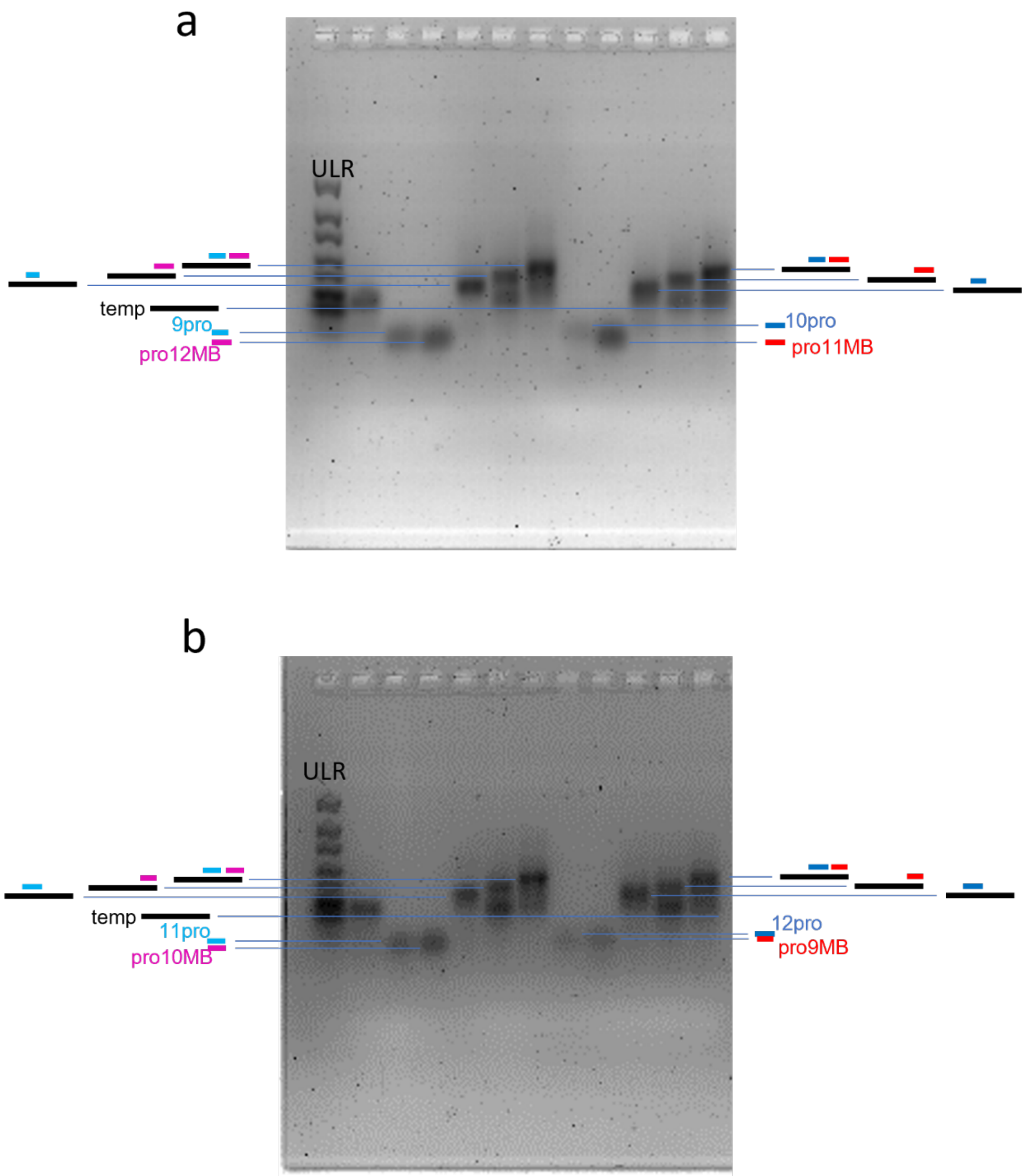

Figure S43: Gel electrophoresis of template strand (black) hybridization to coding strands a) 9pro (cyan) and/or pro12MB (magenta) as well as 10pro (blue) and/or pro11MB (red) and b) 11pro (cyan) and/or pro10MB (magenta) as well as 12pro (blue) and/or pro9MB (red). Lane labeled with ULR contains an Ultra Low Range ladder Gene Ruler from Thermo Fisher. Samples were prepared with template strand at 1.5x in 1x assembly buffer (described in Materials and Methods), heated to 95°C and annealed at 4°C for 30 min. 500 fmol of each sample was loaded on the gel, except for the coding strand alone lanes to which 1500 fmol was added (to try to increase visibility). 3% agarose, 1x TAE, 80 V, 40 mins, pre-stained with SYBR Gold. In every case, addition of a coding strand to the template results in an upward mobility shift, indicating successful hybridization.

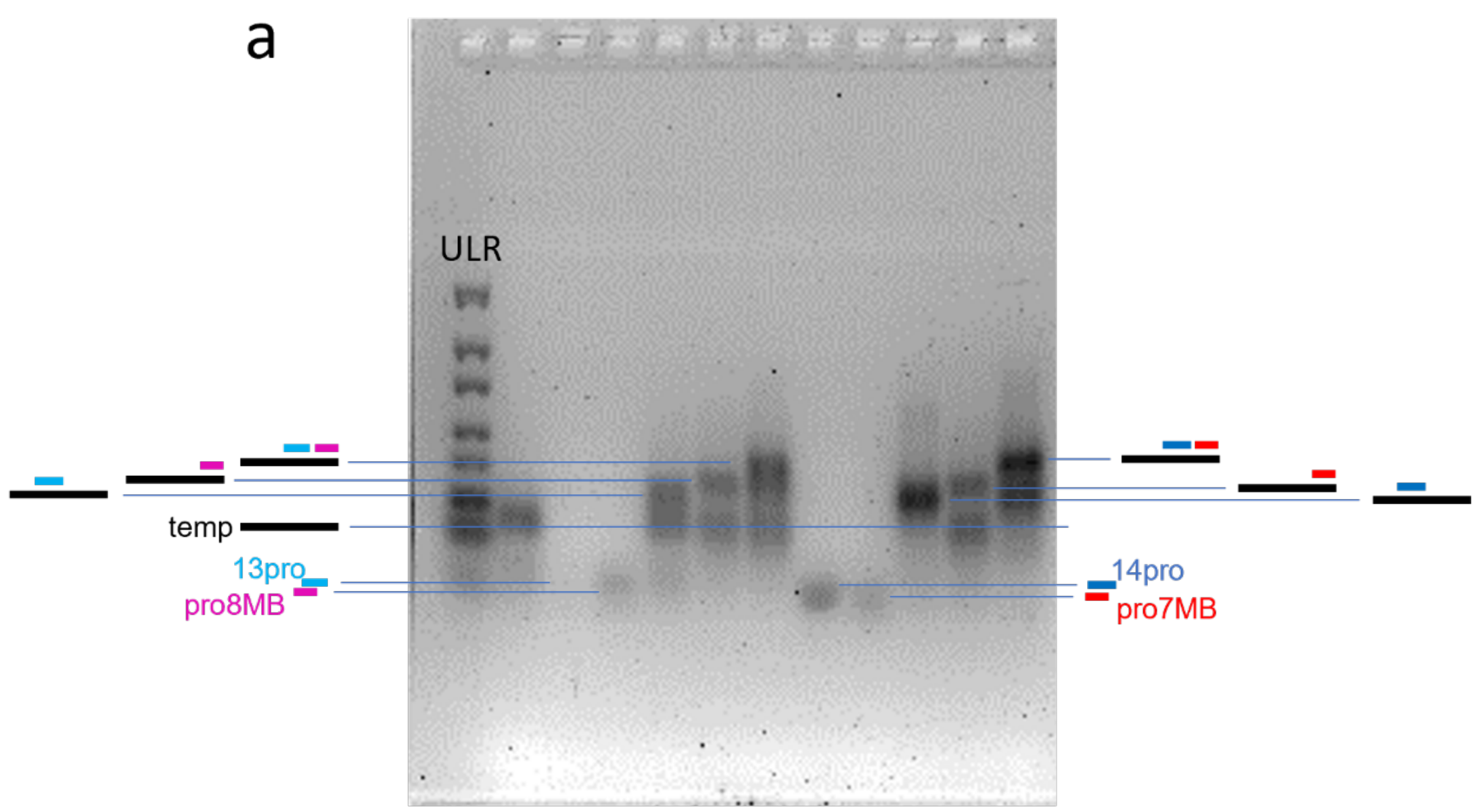

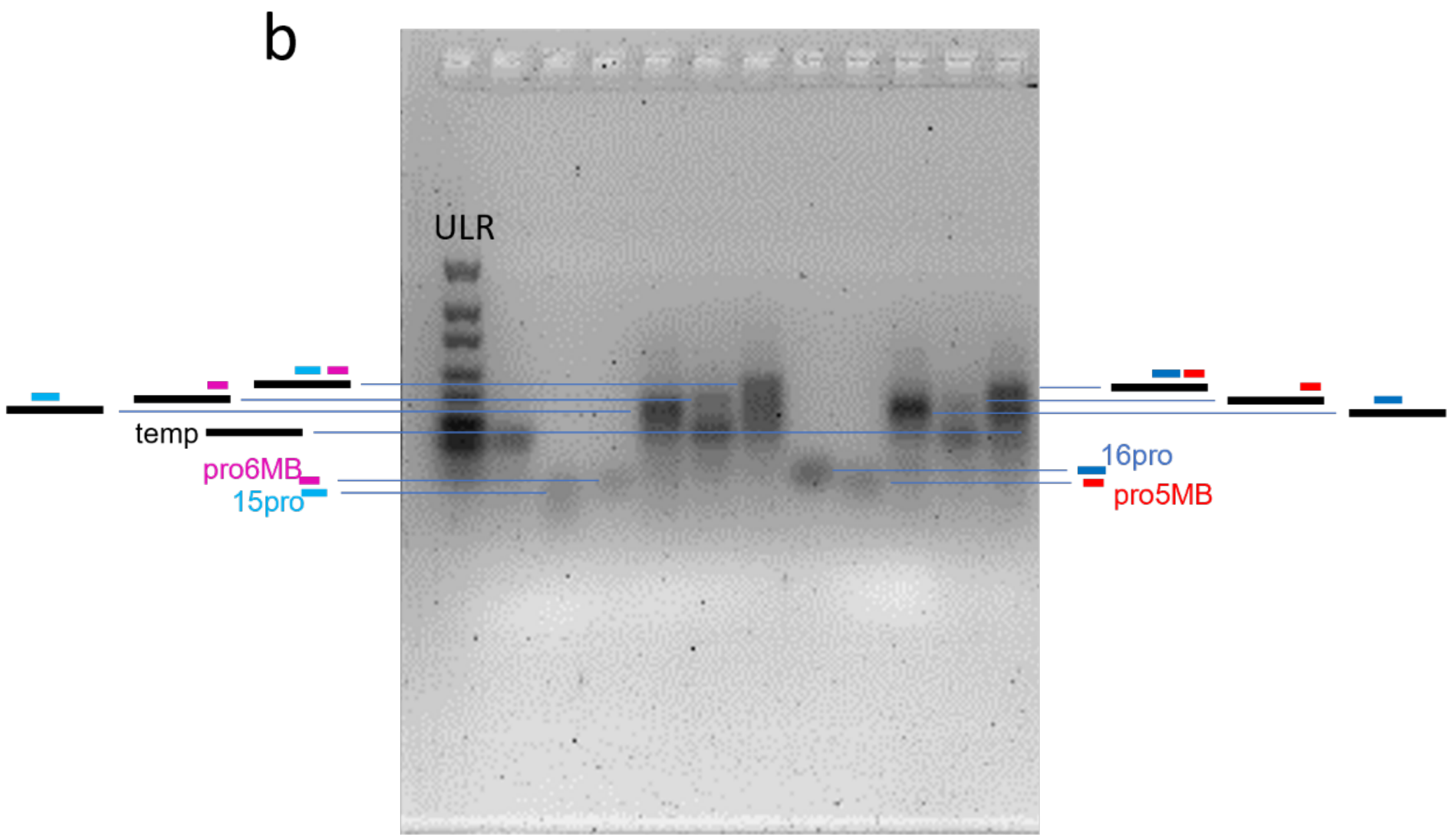

Figure S44: Gel electrophoresis of template strand (black) hybridization to coding strands a) 13pro (cyan) and/or pro8MB (magenta) as well as 14pro (blue) and/or pro7MB (red) and b) 15pro (cyan) and/or pro6MB (magenta) as well as 16pro (blue) and/or pro5MB (red). Lane labeled with ULR contains an Ultra Low Range ladder Gene Ruler from Thermo Fisher. Samples were prepared with template strand at 1.5x in 1x assembly buffer (described in Materials and Methods), heated to 95°C and annealed at 4°C for 30 min. 500 fmol of each sample was loaded on the gel, except for the coding strand alone lanes to which 1500 fmol was added (to try to increase visibility). 3% agarose, 1x TAE, 80 V, 40 mins, pre-stained with SYBR Gold. In every case, addition of a coding strand to the template results in an upward mobility shift, indicating successful hybridization.

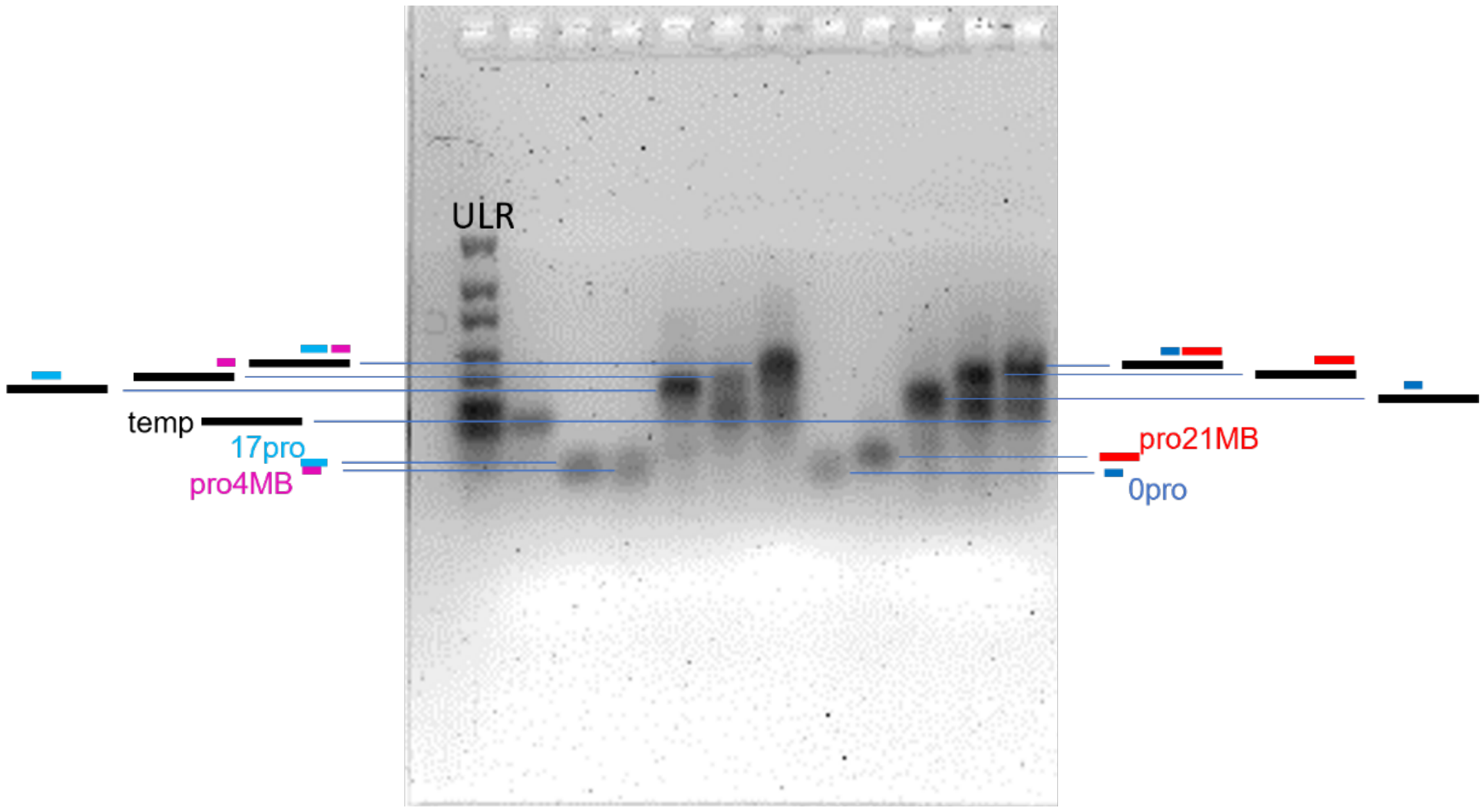

Figure S45: Gel electrophoresis of template strand (black) hybridization to coding strands 17pro (cyan) and/or pro4MB (magenta) as well as 0pro (blue) and/or pro21MB (red). Lane labeled with ULR contains an Ultra Low Range ladder Gene Ruler from Thermo Fisher. Samples were prepared with template strand at 1.5x in 1x assembly buffer (described in Materials and Methods), heated to 95°C and annealed at 4°C for 30 min. 500 fmol of each sample was loaded on the gel, except for the coding strand alone lanes to which 1500 fmol was added (to try to increase visibility). 3% agarose, 1x TAE, 80 V, 40 mins, pre-stained with SYBR Gold. In every case, addition of a coding strand to the template results in an upward mobility shift, indicating successful hybridization.

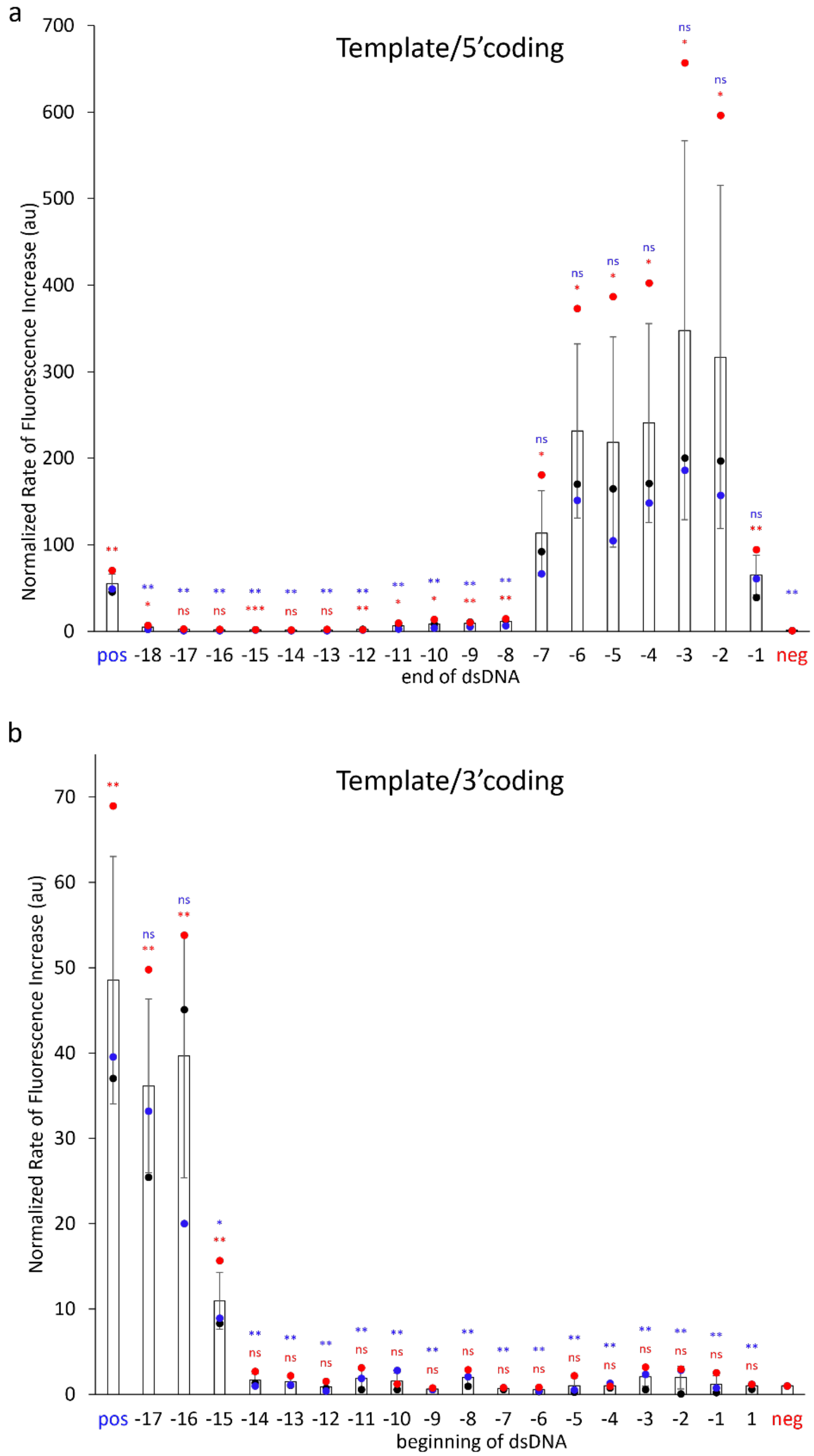

Figure S46: Fluorescence increase during the linear phase of FAM molecular beacon fluorescence intensity transcription tracking of structures with the template strand hybridized to a) the 5' coding strand alone, double-stranded from position -40 to the position indicated, or b) the 3' coding strand alone, double-stranded from the position indicated to position +28, normalized to the 17 nt gap negative control (neg), n=3 (black, blue, red dots), bar graphs are mean, error bars are s.d. Significance marks show one tailed homoscedastic T-tests in relation to the negative control, highlighted red, or the fully double stranded promoter positive control (pos), highlighted blue. ; ns: p>.05, *: p<.05, **: p<.01, ***: p<.001. Linear phase is denoted by red dashed lines in Figure S47. Schematics of structures are shown in Figures S48 and S49.

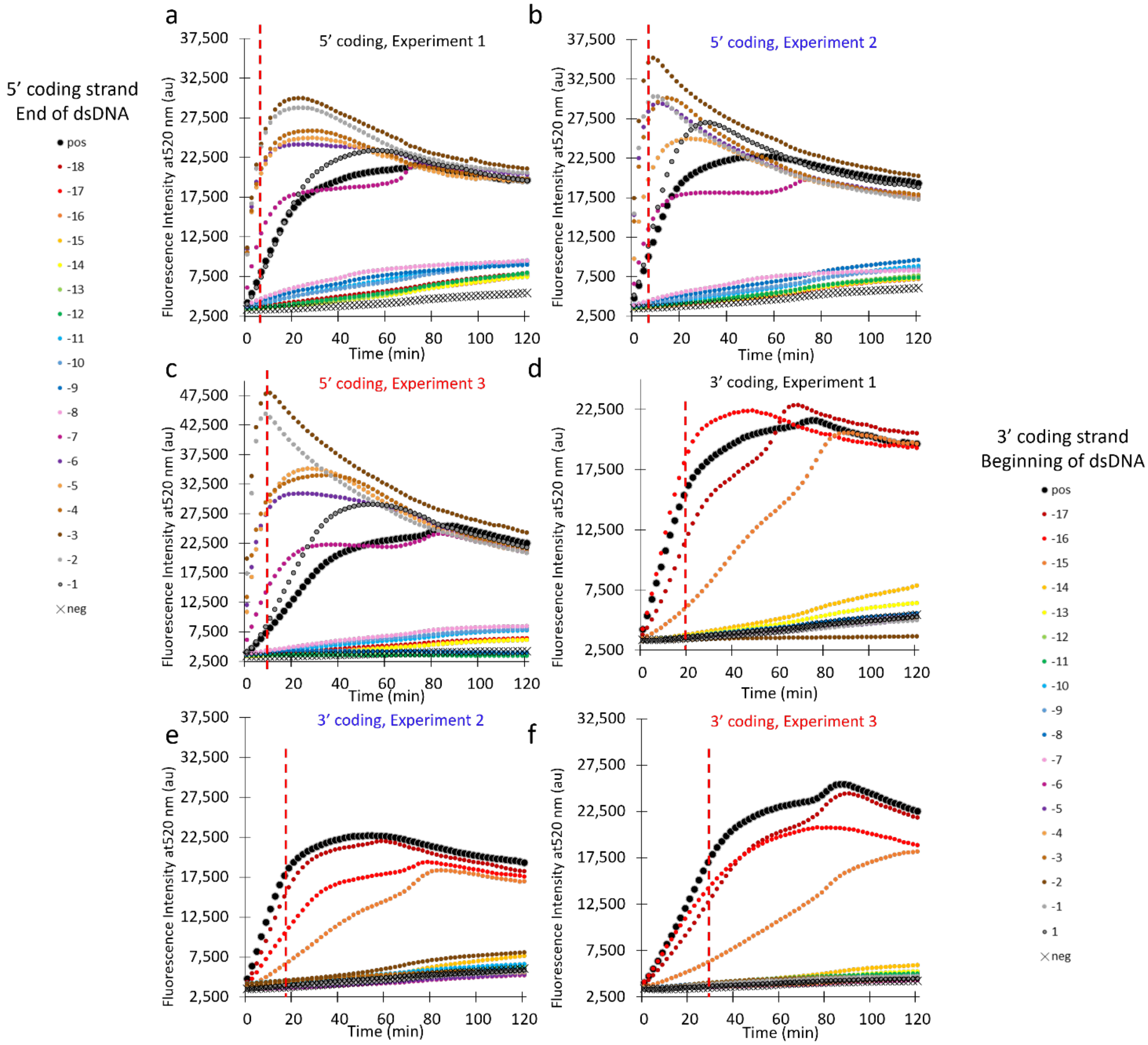

Figure S47: Raw data of FAM molecular beacon fluorescence intensity transcription tracking of structures with the template strand hybridized to the 5’ coding strand alone a) first, b) second, and c) third experimental replicates of the data shown in Figure S46a, structures (Figure S48) are double stranded from position -40 to that indicated by the legend to left and the 3’ coding strand alone d) first, b) second, and f) third experimental replicates of the data shown in Figure S46b, structures (Figure S49) are double stranded from position indicated by the legend to right to position +28. Red dashed lines show where the linear phase ends for the graphs in Figure S46. Positive control (pos) is fully double-stranded and the negative control (neg) has a 17 nt gap in the promoter.

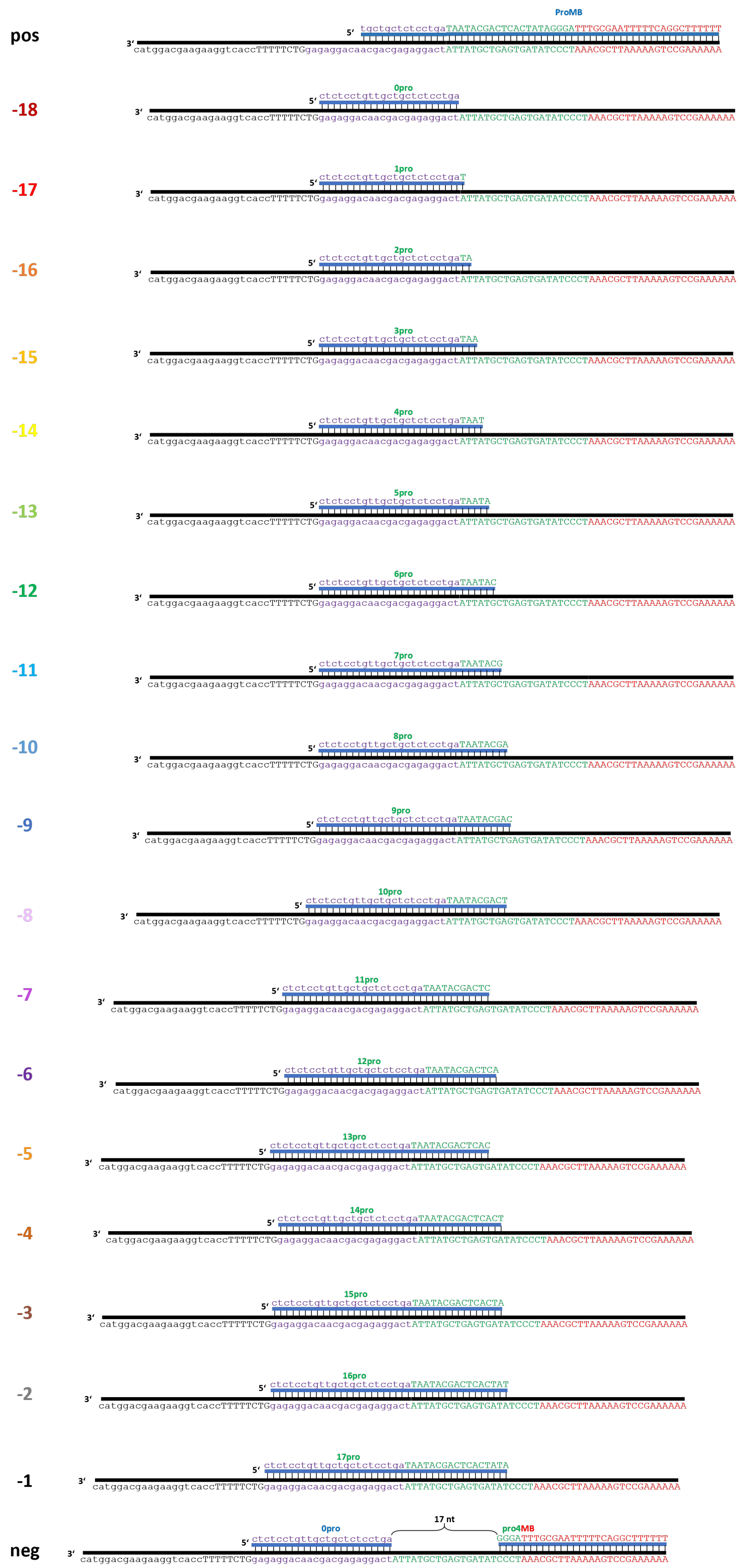

Figure S48: Schematic for structures used in Figures 40a, S46a and S47a-c. Template strands are black, coding strands are blue, and sequence text is colored based on functional region, purple = handle, green = T7 promoter, red = MB target region. Positions indicated on left are colored to correlate with the curve in raw data graphs (Figure S47a-c) that correspond to that structure.

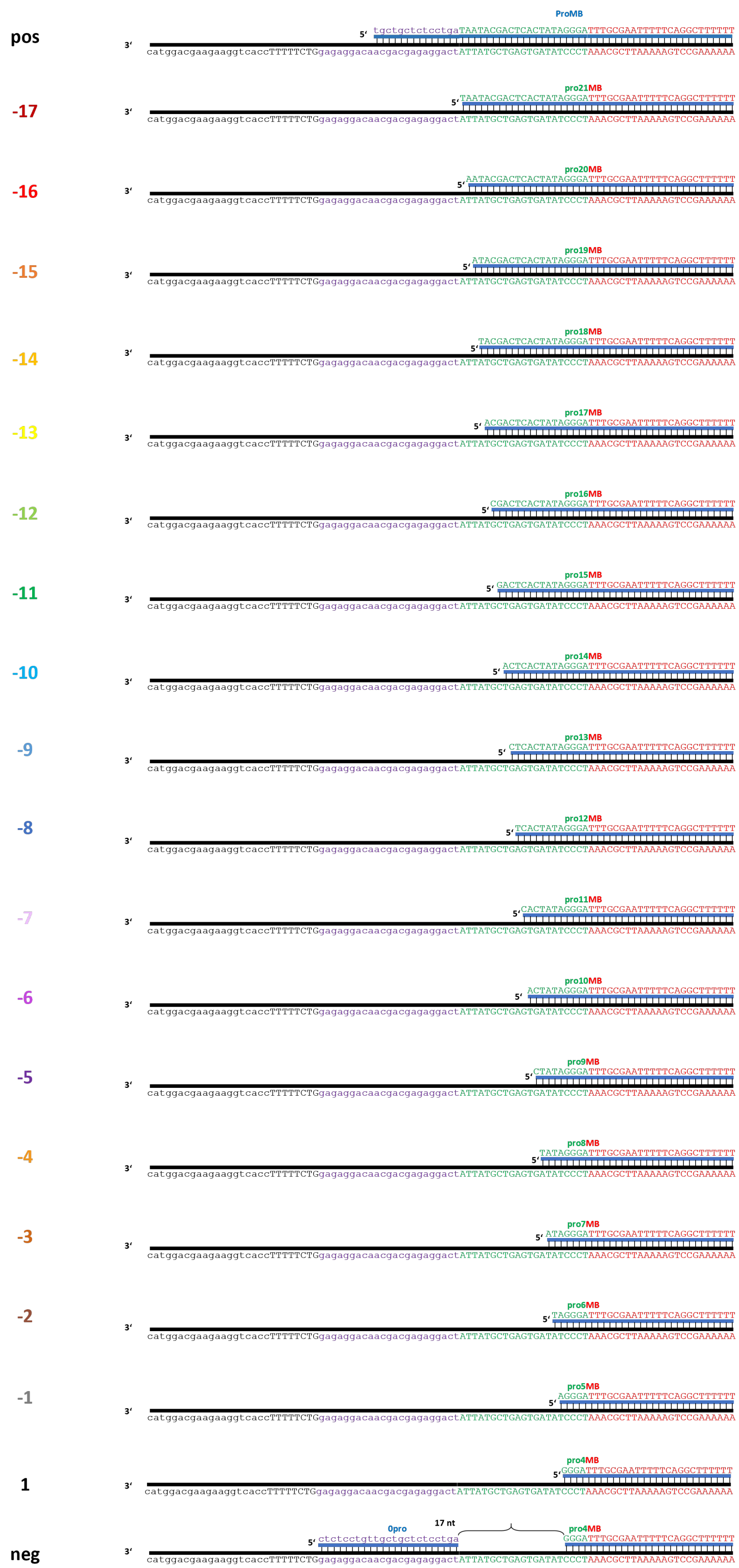

Figure S49: Schematic for structures used in Figures 40b, S46b and S47d-f. Template strands are black, coding strands are blue, and sequence text is colored based on functional region, purple = handle, green = T7 promoter, red = MB target region. Positions indicated on left are colored to correlate with the curve in raw data graphs (Figure S47d-f) that correspond to that structure.

Supplementary Text ST5: on pull-down binding assays:

One explanation for why T7RNAP transcribes certain substrates more efficiently is differences in binding affinity. To test this, we performed pull-down assays in which T7RNAP (immobilized on Ni–NTA beads via its His_6-tag) was incubated with various DNA substrates (Fig. S50), namely a fully double-stranded DNA (DS), DS with a scrambled promoter (DS_{scram}), a 9 bp promoter with an 8 nt gap (9pro/8gap), a 5 bp promoter with a 0 nt gap (5pro/0gap), a 6 bp promoter with a 0 nt gap (6pro/0gap), the single-stranded template strand alone (temp), and temp with a scrambled promoter ($temp_{scram}$). After washing, bound polymerase–DNA complexes were eluted with imidazole, separated by native PAGE, and quantified relative to a DNA ladder to approximate relative binding strengths (Figs. S50-S53). As expected, DS bound T7RNAP most strongly while DS_{scram} showed the weakest band intensity. All other substrates exhibited between 25% (9pro/8gap) and 45% (6pro/0gap) the DS band intensity.

To assess whether binding affinity correlated with transcriptional activity, the same substrates were tested under transcription conditions, with rates plotted on the secondary axis of Figure S50 (yellow crosses; raw data in Fig. S53). Consistent with prior experiments, DS exhibited the strongest transcription, whereas those with DS_{scram}, temp, $temp_{scram}$, and 5pro/0gap were not transcribed. Again, 9pro/8gap transcribed almost as efficiently as DS, though its signal plateaued earlier (Figs. S53a-c). Lastly, 6pro/0gap showed only minimal activity. These results demonstrate that binding affinity does not correlate with transcriptional output for substrates with gaps in the NT strand.

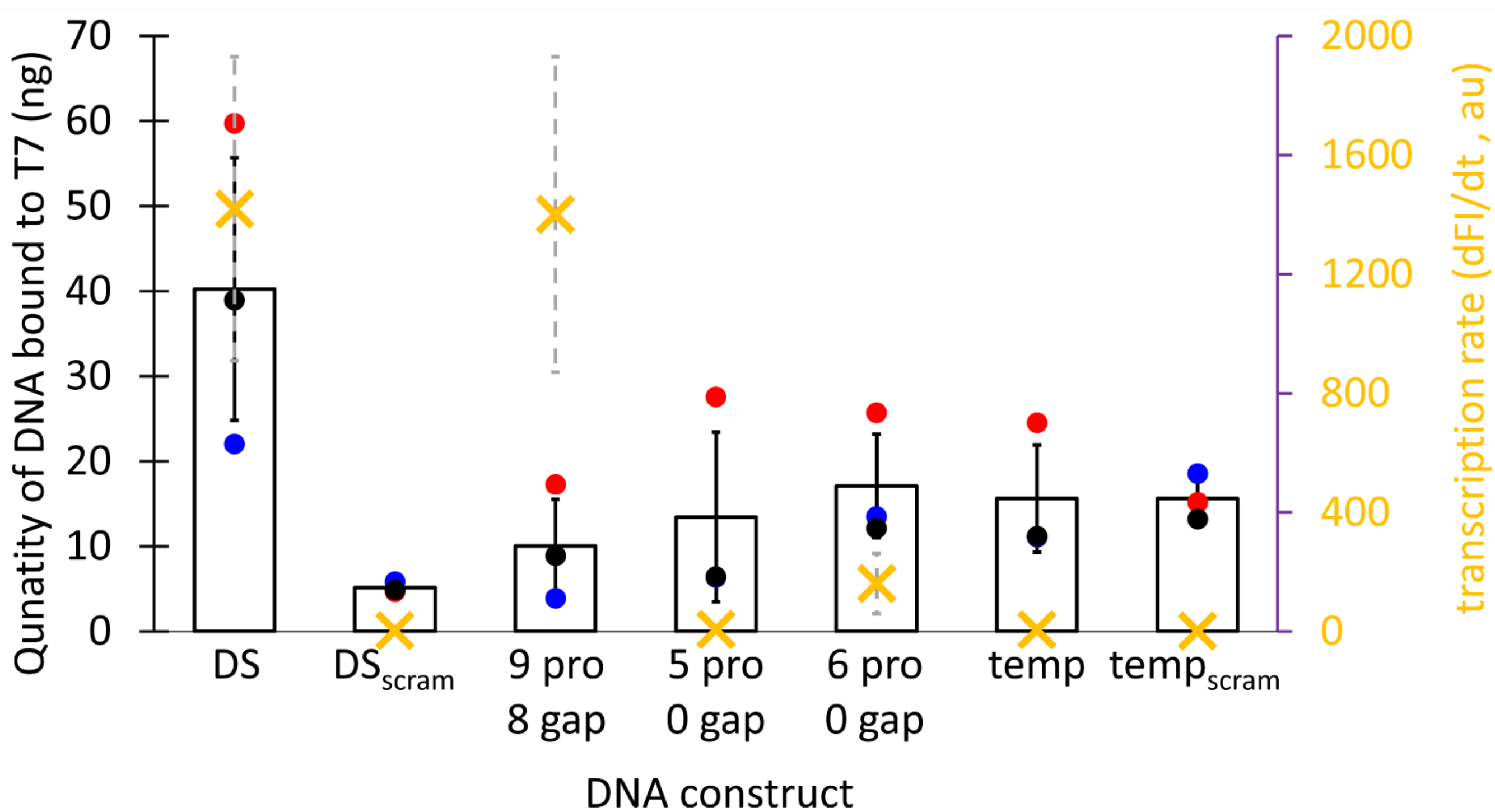

Figure S50: Pull down results showing quantity of DNA bound to T7RNAP (left axis, bar graphs, solid black error bars) versus the rate of transcription (right axis, yellow crosses, dashed grey error bars) for double-stranded (DS) substrates, DS with a scrambled promoter (DS_{scram}), a 9 bp promoter with an 8 nt gap (9pro/8gap), a 5 bp promoter with a 0 nt gap (5pro/0gap), a 6 bp promoter with a 0 nt gap (6pro/0gap), the template strand only (temp), and temp with a scrambled promoter ($temp_{scram}$) . n=3 (black, blue, red dots are individual repeats for left axis), bar graphs and yellow crosses are mean, error bars are s.d.

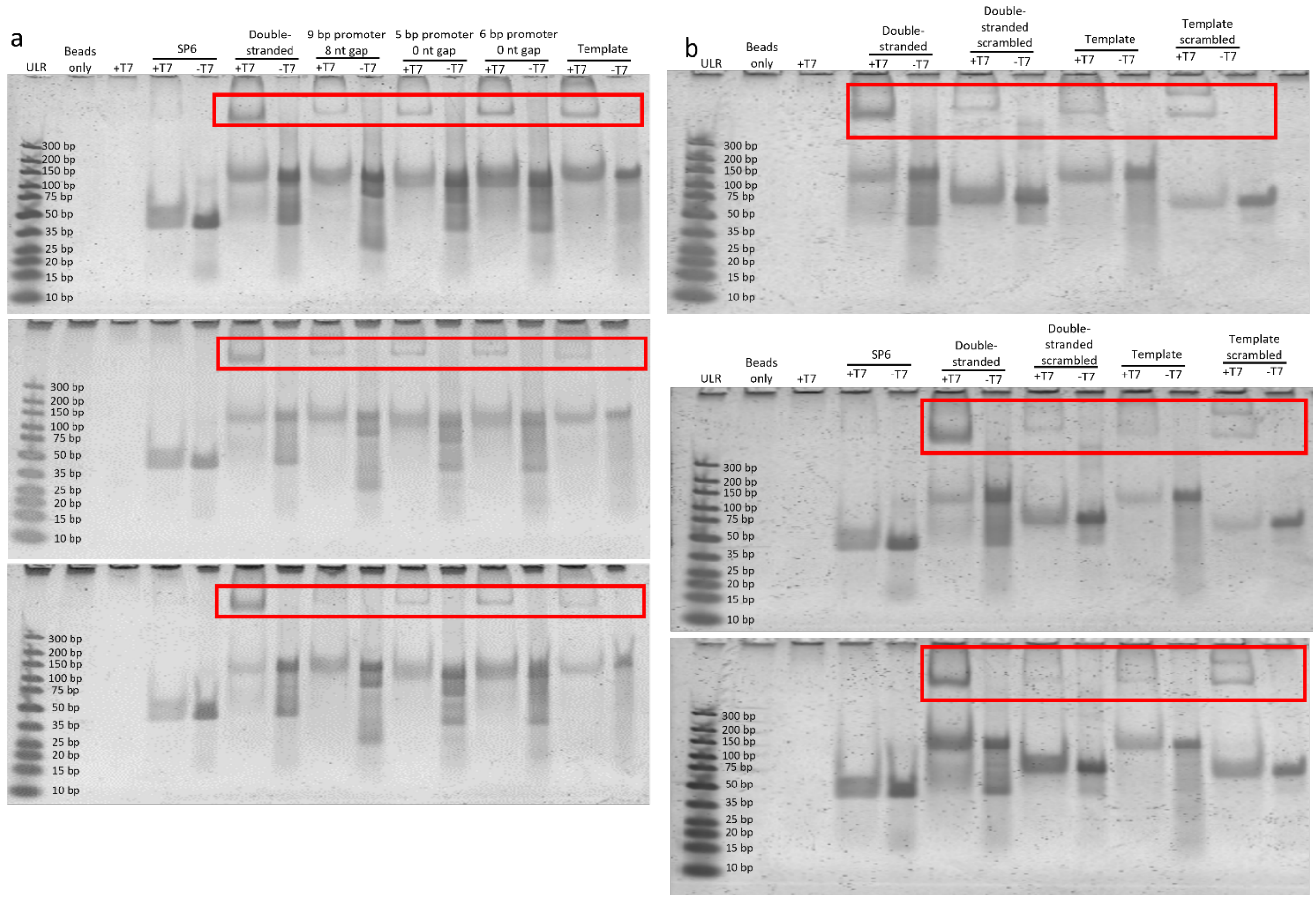

Figure S51: Original native PAGE gels showing each replicate of three experiments each for DNA substrates bound to T7RNAP after magnetic bead pull-down assay for a) non-scrambled and b) scrambled promoters. These gels approximate binding strength, as the stronger the T7RNAP binds to the DNA substrate, the more DNA will be present in the slower running band (red box), presumed to run slower since it is bound to T7RNAP. The lanes labeled SP6 contained DNA with the T7 promoter replaced with the SP6 promoter. The bands in the red box were compared against the ladder (ULR) for quantification, shown in Figure S50. Interestingly, the scrambled template strands seem to result in a double band likely due to secondary structures, both bands were used for quantification. ULR is an ultra-low range ladder from Thermo Fischer. 8% PAGE, 100 V, 35 min, SYBR gold stained.

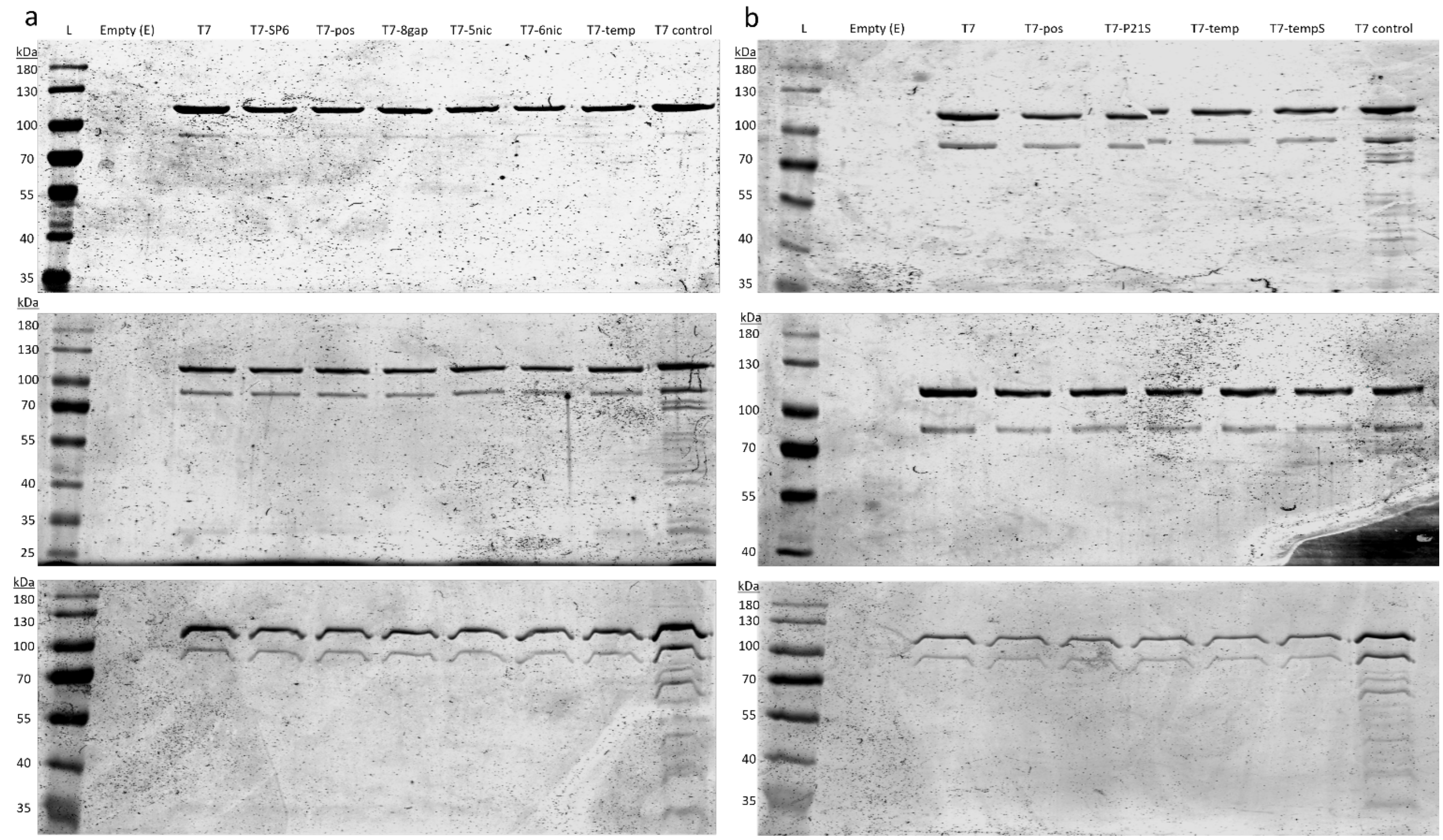

Figure S52: Original Coomassie Brilliant Blue-stained SDS-PAGE gels showing each replicate of three experiments each for T7RNAP presence in eluted samples after magnetic bead pull-down assay for a) non-scrambled and b) scrambled promoters. Loaded 10 µL of each eluted sample. T7 control is 2.5 µM of stock T7 as reference. L is a PageRuler prestained protein ladder from Thermo Fischer. 8% SDS PAGE, 200 V, 45 min.

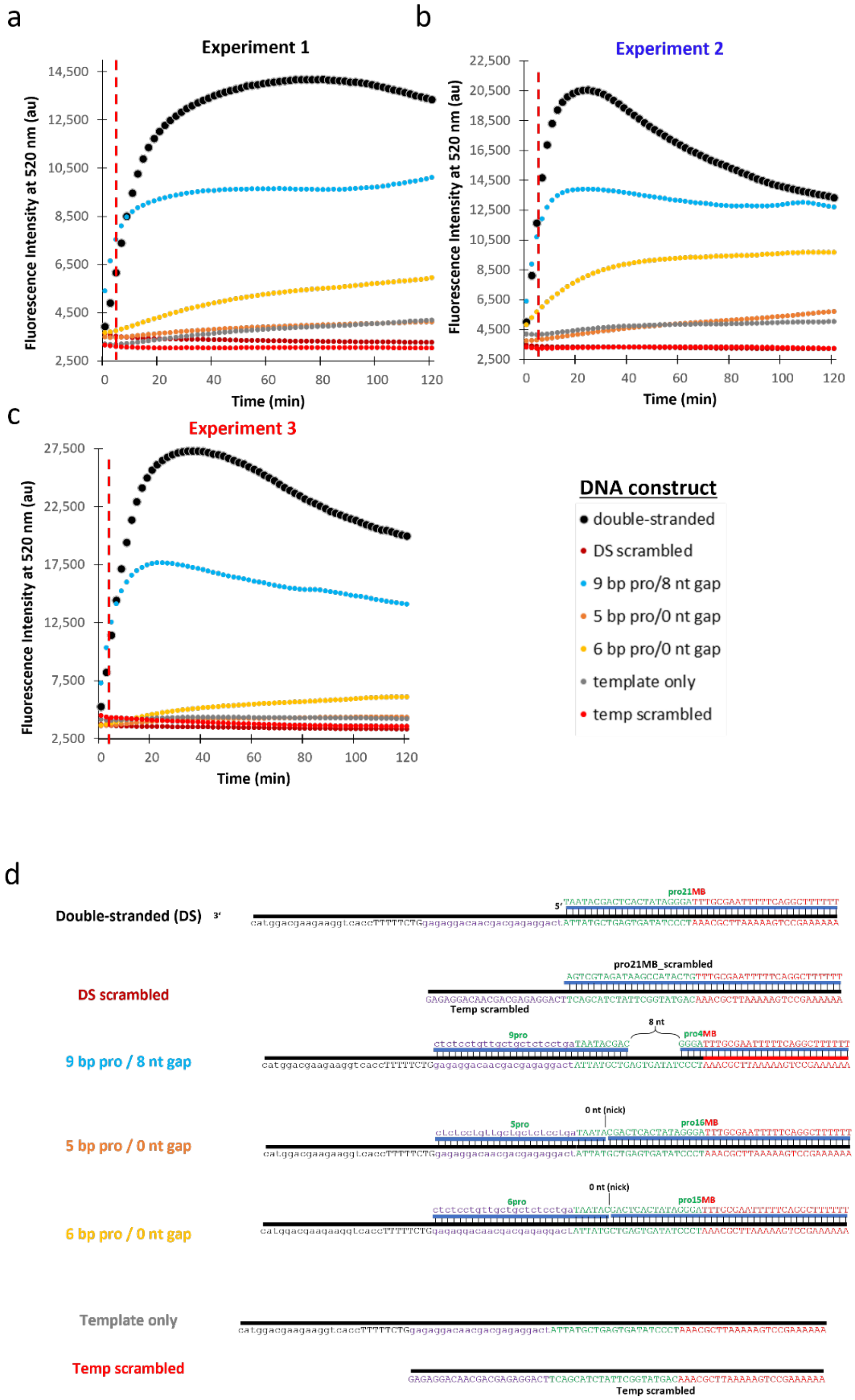

Figure S53: FAM molecular beacon fluorescence intensity transcription tracking for structures used in the binding assays. a) raw data for the first experimental repeat, b) raw data for the second experimental repeat, and c) raw data for the third experimental repeat. Red dashed lines show linear phase for calculating transcription rate in secondary axis of Figure S50. Legend shows what color line in a-c correlates to what structure shown in d) schematics for the structures used in this experiment.

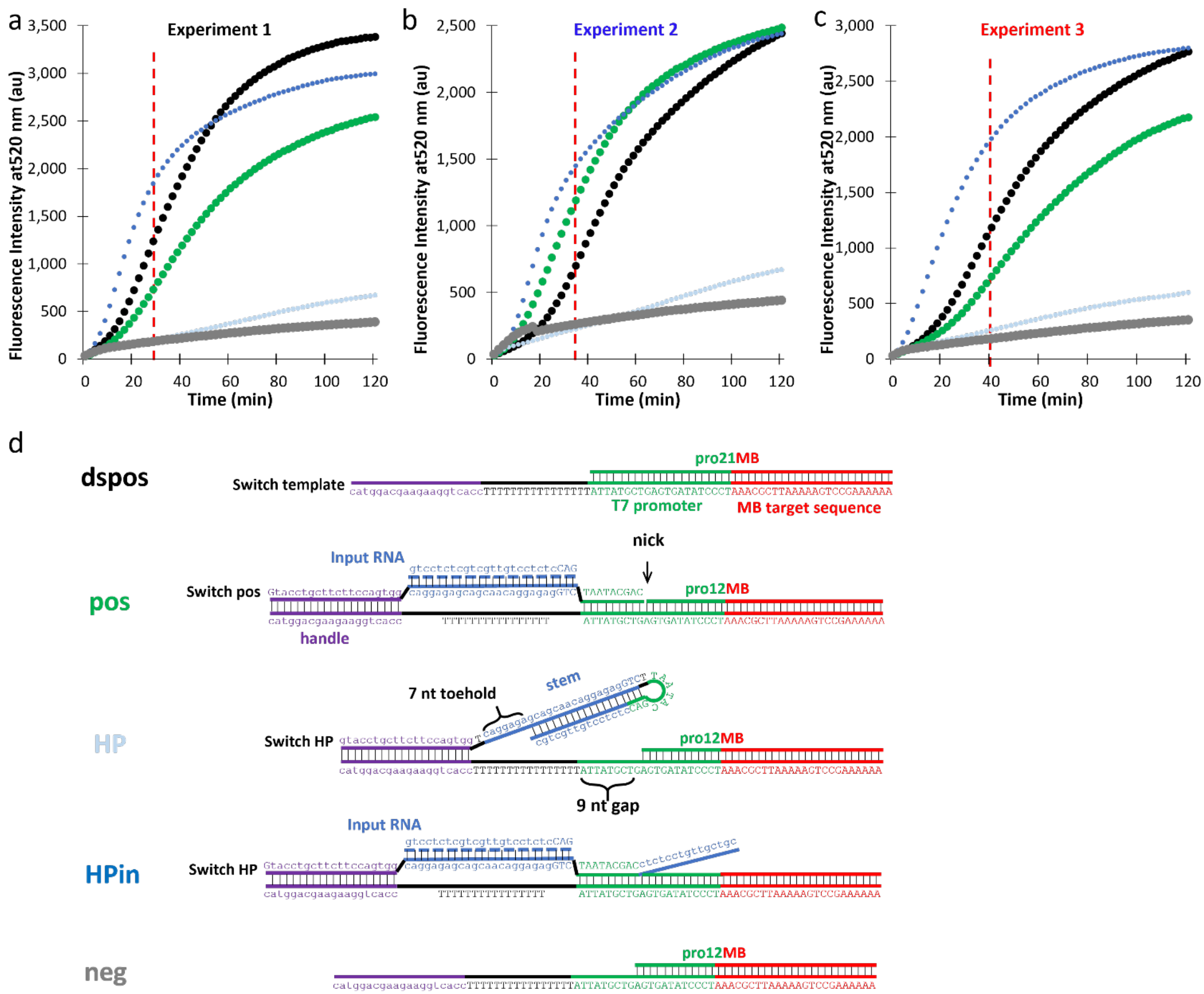

Figure S54: Raw data for FAM transcription fluorescence intensity transcription tracking for a) the first experimental repeat (black dots in Figure 3b), b) the second experimental repeat (blue dots in Figure 3b), c) for the third experimental repeat (red dots in Figure 3b) for the hairpin switch demonstrating control over transcription. Red dashed lines show where the linear phase ends for rate calculation. Curve colors correspond to the legend in d) schematics for structures used in test. Positive control (dspos) has a fully double-stranded promoter and the negative control (neg) has a promoter with positions -17 to -9 single-stranded. Additional control (pos) has the same base-pairing context upstream of the promoter region as the switch with input strand attached, but is unable to form a hairpin thereby serving as an always on positive control. HP is the hairpin switch without input RNA (dashed line), HPin is the hairpin switch with the input RNA added at 10x excess. Input RNA is also added to pos and neg at 10x excess.

REFERENCES

[1] J. N. Zadeh, C. D. Steenberg, J. S. Bois, B. R. Wolfe, M. B. Pierce, A. R. Khan, R. M. Dirks, N. A. Pierce, "NUPACK: Analysis and design of nucleic acid systems" *Journal of Computational Chemistry* **2011**, *32*, 170-173.

[2] R. M. Dirks, J. S. Bois, J. M. Schaeffer, E. Winfree, N. A. Pierce, "Thermodynamic analysis of interacting nucleic acid strands" *Siam Review* **2007**, *49*, 65-88.

[3] M. E. Fornace, N. J. Porubsky, N. A. Pierce, "A Unified Dynamic Programming Framework for the Analysis of Interacting Nucleic Acid Strands: Enhanced Models, Scalability, and Speed" *ACS Synthetic Biology* **2020**, *9*, 2665-2678.

[4] J. F. Milligan, D. R. Groebe, G. W. Witherell, O. C. Uhlenbeck, "Oligoribonucleotide Synthesis Using T7 Rna-Polymerase and Synthetic DNA Templates" *Nucleic Acids Research* **1987**, *15*, 8783-8798.

[5] S. W. Schaffter, E. Kengmana, J. Fern, S. R. Byrne, R. Schulman, "Strategies to Reduce Promoter-Independent Transcription of DNA Nanostructures and Strand Displacement Complexes" *ACS Synthetic Biology* **2024**, *13*, 1964-1977.

[6] B. B. Yu, Y. F. Chen, Y. Yan, X. L. Lu, B. Zhu, "DNA-terminus-dependent transcription by T7 RNA polymerase and its C-helix mutants" *Nucleic Acids Research* **2024**, *52*, 8443-8453.

[7] M. Maslak, C. T. Martin, "Kinetic-Analysis of T7-Rna Polymerase Transcription Initiation from Promoters Containing Single-Stranded Regions" *Biochemistry* **1993**, *32*, 4281-4285.

[8] R. Padmanabhan, S. N. Sarcar, D. L. Miller, "Promoter Length Affects the Initiation of T7 RNA Polymerase In Vitro New Insights into Promoter/Polymerase Co-evolution" *Journal of Molecular Evolution* **2020**, *88*, 179-193.

[9] Q. Guo, R. Sousa, "Multiple roles for the T7 promoter nontemplate strand during transcription initiation and polymerase release" *Journal of Biological Chemistry* **2005**, *280*, 3474-3482.
